# Testing for the Effects and Consequences of Mid Paleogene Climate Change on Insect Herbivory

**DOI:** 10.1371/journal.pone.0040744

**Published:** 2012-07-18

**Authors:** Torsten Wappler, Conrad C. Labandeira, Jes Rust, Herbert Frankenhäuser, Volker Wilde

**Affiliations:** 1 Steinmann Institute, University of Bonn, Bonn, Germany; 2 Department of Paleobiology, National Museum of Natural History, Smithsonian Institution, Washington, District of Columbia, United States of America; 3 Department of Entomology and BEES Program, University of Maryland, College Park, Maryland, United States of America; 4 Mainz Natural History Museum/State Collection for Natural History of Rhineland-Palatine, Mainz, Germany; 5 Senckenberg Forschungsinstitut und Naturmuseum, Paläobotanik, Frankfurt am Main, Germany; Raymond M. Alf Museum of Paleontology, United States of America

## Abstract

**Background:**

The Eocene, a time of fluctuating environmental change and biome evolution, was generally driven by exceptionally warm temperatures. The Messel (47.8 Ma) and Eckfeld (44.3 Ma) deposits offer a rare opportunity to take a census of two, deep-time ecosystems occurring during a greenhouse system. An understanding of the long-term consequences of extreme warming and cooling events during this interval, particularly on angiosperms and insects that dominate terrestrial biodiversity, can provide insights into the biotic consequences of current global climatic warming.

**Methodology/Principal Findings:**

We compare insect-feeding damage within two middle Eocene fossil floras, Messel and Eckfeld, in Germany. From these small lake deposits, we studied 16,082 angiosperm leaves and scored each specimen for the presence or absence of 89 distinctive and diagnosable insect damage types (DTs), each of which was allocated to a major functional feeding group, including four varieties of external foliage feeding, piercing- and-sucking, leaf mining, galling, seed predation, and oviposition. Methods used for treatment of presence–absence data included general linear models and standard univariate, bivariate and multivariate statistical techniques.

**Conclusions/Significance:**

Our results show an unexpectedly high diversity and level of insect feeding than comparable, penecontemporaneous floras from North and South America. In addition, we found a higher level of herbivory on evergreen, rather than deciduous taxa at Messel. This pattern is explained by a ca. 2.5-fold increase in atmospheric CO_2_ that overwhelmed evergreen antiherbivore defenses, subsequently lessened during the more ameliorated levels of Eckfeld times. These patterns reveal important, previously undocumented features of plant-host and insect-herbivore diversification during the European mid Eocene.

## Introduction

**Figure 1:**
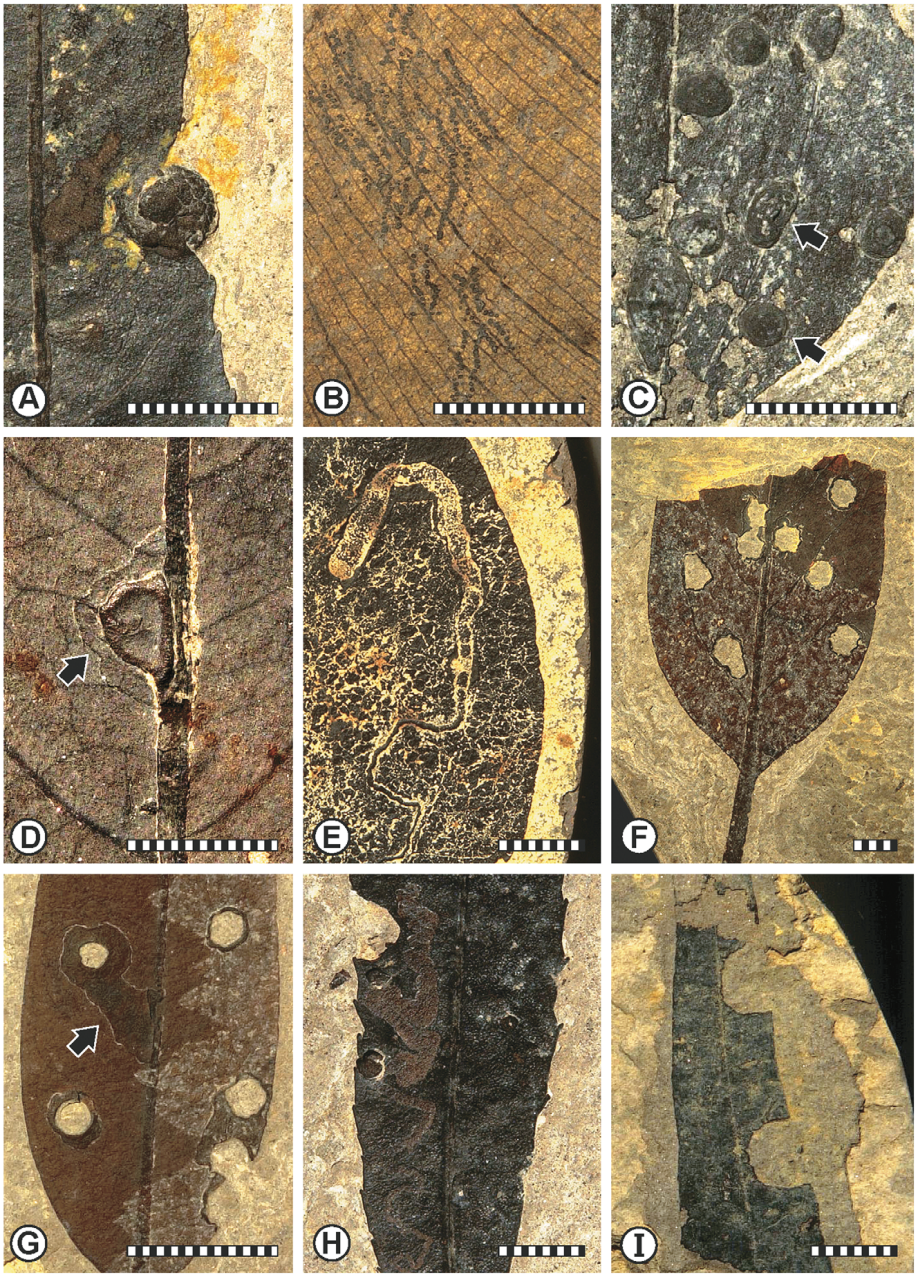
Examples of plant damage representing a broad spectrum of insect herbivory from the Messel and Eckfeld maar lake localities. A. Well preserved gall with delineation of concentric growth striae [DT163] (SMF Me 3591). B. Paired mandible chew marks on *Araciphyllites tertiarius* [DT219] (SMF Me 1396). C. Elongate and round scales of scale insects preserved in situ on an leaf blade (arrows) [DT191] (PB 2005-140, LS, NHMM) [40]. D. A broad zone of gall necroses on surrounding plant tissue [DT163] (SMF Me 3198). E. Mine with a distinctively quadrangular terminal chamber [DT171] (SMF Me 3582). F. Gall attachment scars on a lauraceous leaf fragment [DT206] (SMF Me 21180). G. Circular holes characterized by a broad flange of reaction tissue (arrow) [DT206] (SMF Me 21184). H. A strongly undulatory, serpentine mine consisting of modest width increases and containing particulate frass, on a walnut leaf (Juglandaceae) [DT92] (SMF Me 13228). I. An unidentified dicot exhibiting typical margin excisions, most likely produced by megachilid bee [DT82] (PB 1990-527, LS, NHMM) [101]. Scale bar = 1 cm.

For plants, the Eocene appears to have been one of the most biologically diverse intervals in Earth history [1]–[3], and is associated with an extensive migration of the subtropical rainforest biome into mid-latitudinal regions [4], [5]. This trend began during the Paleocene–Eocene Thermal Maximum (PETM) at 55.8 Ma, an event defined by a dramatic carbon isotope excursion, as recorded by proxy data of benthic foraminifera [6] associated with increased atmospheric CO_2_ and a global spike in elevated temperature. The discovery of other, smaller magnitude, rapid greenhouse warming events (hyperthermals) at several millions of years following the PETM provides additional opportunities to examine the response of organisms to rapid global climate change in the terrestrial realm [7]–[9]. These hyperthermals are of great interest, as they provide potential analogs for a future greenhouse world. While transitional climate regimes across the Paleocene–Eocene boundary and their impact on insect herbivory previously have been documented at a high level of resolution [10]–[12], little is known about the trophic stability of later, middle Eocene ecosystems [13]. Nonetheless, a few studies evaluating insect herbivory are available from the middle Eocene (Lutetian, 49–41.3 Ma) [14], [15]. These and other studies reveal that the inventory of insect damage on plant hosts constitutes an impressive spectrum of plant–insect associations [16]–[18] (Fig. 1), illustrating important, previously unknown aspects of plant-host and insect-herbivore diversification for the European Eocene.

The maar deposits of Messel and Eckfeld Maar are well known for a broad spectrum of fossils ranging from organic molecules, micro-organisms, aquatic invertebrates, plants and insects and their varied associations, to a wide range of vertebrates including articulated mammals exhibiting soft tissue preservation and gut contents [19]–[22]. Newly available paleoclimatic evidence from these localities indicate that the regional Eocene climate dynamics were characterized by a warm-temperate to subtropical regime [23], [24] that allowed for warm temperatures even above the Arctic Circle ([25] and references therein), and throughout the duration of the Eocene when global mean temperatures became generally cooler [13], [26]. Here, we propose a framework to evaluate how terrestrial food webs evolved approximately 48–44 million years ago, a time of considerably elevated atmospheric CO_2_ concentration [27], coupled with thermal maxima and the mammalian species-diversity climax of the earlier middle Eocene [22], [28]. Additionally, our material allows a test of whether changes in temperature, atmospheric CO_2_, and floral diversity as observed across Eocene global warming events correlate with changes in insect damage frequency, diversity, and composition. These data and the present study of insect herbivory on fossil leaves provide crucial information on the ecology of feeding associations and relationships between plants and their insect herbivores that are impossible to obtain separately from the macrofossil record of plant and insect body fossils. The European middle Eocene previously has not been subject to such detailed analyses of plant–insect associations; as well, we present a significant and new opportunity for understanding Eocene regional community ecology and climate dynamics and how these factors differ from those of the present day. In particular, the deposits of Messel and Eckfeld offer a rare opportunity for a census of deep-time ecosystems during a protracted interval of greenhouse climate.

## Results

### Floristic Composition and Richness

**Figure 2:**
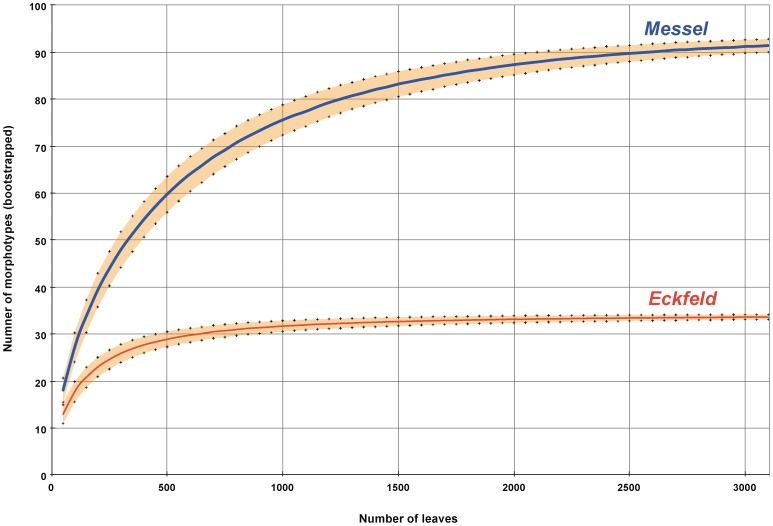
Rarefaction curves comparing the number of angiosperm leaf morphotypes at Messel and Eckfeld. The shaded area represents the standard error of the rarefaction calculated after [102]. The horizontal scale is reduced to 3000 for greater detail.

The Messel and Eckfeld sites are considerably different based on their representation and diversity of leaf taxa. Eckfeld appears significantly poorer in leaf diversity, consisting of 33 leaf morphotypes compared to 93 recorded from Messel. The data suggest that Messel had a high plant diversity, comparable to that of the modern subtropics [29] or the mid-latitudinal Eocene macrofloral sites at Laguna del Hunco in southern Patagonian Argentina [30], [31]. In order to examine plant richness standardized for sample size, rarefaction curves were plotted for Messel and Eckfeld (Fig. 2). Differences in leaf diversity between the two localities were statistically significant (*p*<0.001), tested on the basis of a binomial generalized linear model. Even though detailed taxonomic assignments to lowest ranks often were not feasible, there is unequivocal recognition of plant families, and all distinct leaf morphotypes are known for both localities [32], [33].

Typical floral elements from both localities consist of woody, evergreen trees and shrubs, of which the Juglandaceae and Lauraceae constitute 65% of leaves at Messel and ca. 40% of the leaves at Eckfeld. Over two-thirds of the plant morphotypes are represented by less than five percent of the leaf taphocoenoses. Consequently, there is minimal difference in evenness between Messel and Eckfeld at the locality level (Table 1). Evenness is the relative abundance with which each morphotype or DT is represented in a sample. Simpson’s diversity index D is equal to 0.94 at Messel and 0.86 at Eckfeld, indicating that 94 and 86 pairs, respectively, out of 100 taken at random are composed of different morphotypes. The analogous Pielous’ J is at Messel 0.76 and 0.74 at Eckfeld. Other plant families of mostly modern tropical-subtropical provenance were present at Messel, principally Fabaceae (∼ 6%), Ulmaceae (4%), and Moraceae (∼ 4%). In addition to many leaf morphotypes of uncertain affinity at Eckfeld, there are entire-margined leaves (“laurophyll”), spiny leaves (e.g. *Pungiphyllum waltheri* (∼ 9%) [34], and leaves of Ulmaceae (8%), Theaceae (“*Ternstroemites*” [∼9%]) and Fabaceae (4%), all of which are important as elements of a zonal vegetation surrounding the isolated maar lakes [33]. In both localities the immediately surrounding forest clearly was dominated by multiple species of Juglandaceae, Lauraceae, legumes, Vitaceae and other lineages whose living relatives have climbing, creeping, or entwining habits [33], [35]. Estimated leaf mass per area (*M_A_*) values for all species–locality pairs with at least 25 censused leaves and two measurable fossils at each locality, using the method of Royer et al. [36], are generally higher for Messel (123, [+15/−13 g/m^2^]) than for Eckfeld (111, [+21/−17 g/m^2^]), for a mean and 95% prediction interval, which is asymmetrical, based on log relationships. However, there are no significant among-locality differences in *M_A_* (an ANOVA of *M_A_* by localities yielded an *F* value of 0.51 and *p* = 0.48, df = 1, 19).

**Table 1:** Site summaries and diversity parameters analyzed for plant-species assemblages from the two study localities (Messel, Eckfeld).

*Locality*	*Age (Ma)*	*MAT (°C)*	*MAP (mm)*	*N*	*S*	*Diversity1000 morphotypes* §	*Simpson’s Evenness*	*Pielou’s Evenness J*
**Messel**	47.8 [^86^]	22.4 [^24^]	1671 [^24^]	9334	93	75.61±3.23	0.94	0.76
**Eckfeld**	44.3 [^93^]	17.3 #	na	6748	33	31.71±1.16	0.86	0.74

Abbreviations for indices: MAT, Mean annual temperature; MAP, Mean annual precipitation; N, Number of leaves in the census; S, total number of morphotypes.

# *Leaf margin analysis was used for the Eckfeld sample (Wappler unpubl. data).*

§ *Diversity was rarefied to 1000 leaves using analytical rarefaction and the error represents Heck’s standard error* [*102*] *.*

### Damage Diversity

**Figure 3:**
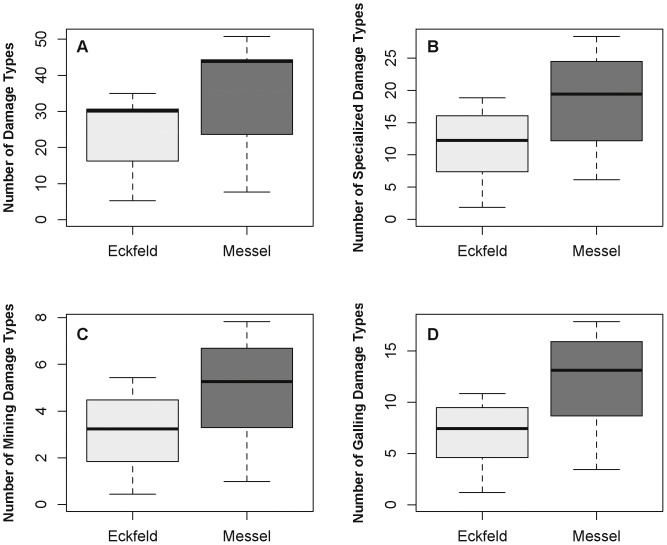
Mean diversity of damage types (DTs) on the bulk floras rarified to 800 leaves. A. Mean diversity of damage types (DTs) on the bulk floras. B. Specialized DTs. C. Mining DTs. D. Galling DTs. The horizontal line is the median, the top and bottom horizontal margins of the box are respectively the upper and lower quartiles, and the vertical lines are the full range of values from the data.

Damage diversity for the bulk leaf floras at Messel and Eckfeld, as well as mine and gall damage-type (DT) diversity of the specialized feeders, are shown in Fig. 3. Measures of diversity are rarified to 800 fossil specimens and a t-test showed that Messel had a higher mean damage diversity than Eckfeld (t = 3.4981, *p* = 0.006). Based on the results of chi-square tests, we conclude that for each functional feeding group there is a highly significant departure from the null expectation indicated between the localities (χ^2^ = 53.4, df = 4, *p*≪0.001). At least 89 distinctive DTs could be recognized from Messel and 68 DTs from Eckfeld. To underscore this diversity, we note that the highest insect herbivore damage diversity from warm-interval PETM and Early Eocene Climatic Optimum (EECO) leaf floras are known from the USA and South America, and yield a considerably lower amount of damage diversity, ranging from 36.6 to 40.1, respectively, for the PETM and EECO North American sites [10], and 35.0 for the EECO South American site [10], [37], when sample-standardized damage diversities were rarified to 800 specimens. These standardized diversities are equivalent to 50.8±3.9 DTs recognized at Messel (Fig. 3A). The results indicate a 25% increase in DT values for European mid-latitude plant–insect associations, particularly for those highly specialized interactions such as mining (χ^2^_Mines_ = 3.9, df = 2, *p* = 0.04) (Fig. 1E, H) and galling (χ^2^_Galls_ = 28.5, df = 2, *p*<0.001) (Fig. 1A, D). This may be explained by the observation that high host specificities overwhelmingly characterize most known lineages of extant gallers and miners. A current worldwide pattern, as reported by previous studies, documents a relationship between high gall species diversity and sclerophyllous, evergreen vegetation [38], [39]. The same type of vegetation dominated much of the central European, mid-latitudinal region during the middle Eocene.

Most damaged specimens showed evidence of margin feeding at both localities, the most common functional feeding group with 857 occurrences at Messel and 391 at Eckfeld. After surface feeding, galling is the third most common type of damage, with 516 occurrences (29 DTs) at Messel but only 160 occurrences (19 DTs) at Eckfeld. Mining is the fourth most common type present, with 285 occurrences at Messel representing 23 DTs, and 72 occurrences at Eckfeld amounting to 18 DTs. Thus, the relative incidence of both mining and galling at Messel is about fifty percent higher than in Eckfeld. The lowest frequency of occurrence (16 at Messel and 9 at Eckfeld) can be found in the case of piercing-and-sucking damage [40]. We also noted that, in the relatively narrow 3 million-year time interval between deposition of Messel and Eckfeld, there is a change in the composition of the herbivore fauna, recognizable by a decreasing prevalence of DTs (Table 2). If only specialized DTs are counted, 45% of the DTs present at Messel are only recorded also from the Eckfeld sample, supporting the hypothesis that higher herbivore diversity additionally may be promoted if plant–herbivore interactions are specialized. This trend is attributable to finely partitioned plant resources that facilitate species coexistence [41], [42]. In addition, such highly specialized associations are consistent with the Janzen-Connell model for increased plant-host and insect herbivore diversity when applied to the post-seedling stage, indicating that host-specialists are responsible for most of the damage to tropical plants [43]–[46].

**Table 2:** Occurrence of damage types (DTs) recorded from Messel and Eckfeld and DTs that are already known from other European floras.

*Locality*	*DTs*						
	*HF*	*MF*	*SF*	*G*	*M*	*PS*	*OV*
**Messel**	14	11	12	29	23	5	4
***known from other European floras*** §	14	9	5	16	10	2	2
**Eckfeld**	13	11	8	19	18	2	1
***known from other European floras*** §	12	9	3	15	10	2	1

Abbreviations are: HF, hole feeding; MF, margin feeding; SF, surface feeding; G, galling; M, leaf mining; PS, piercing and sucking, and OV, oviposition. Fifty-five new DTs, including seed predation and skeletonitazion but not shown in the table, are recognized from both localities.

§ *Damage type diversity data taken from (*[*16]*, [25], [40], [48], [49], [101], [103], *[104]* *, Wappler unpubl. data).*

### Damage Frequency

**Figure 4:**
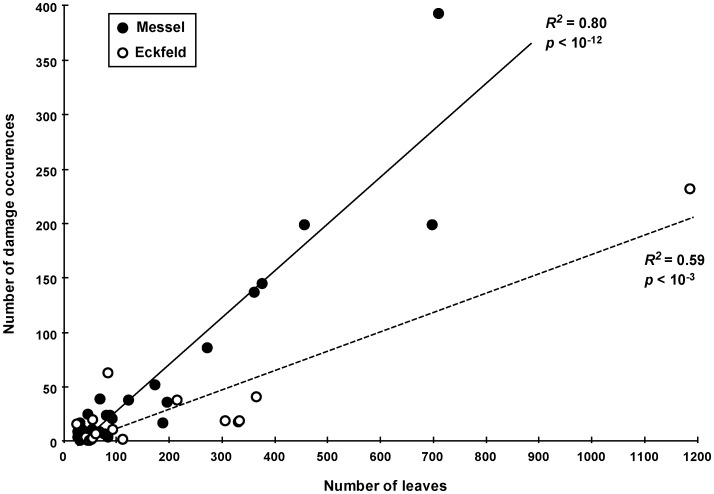
A comparison of damage frequencies between the Messel and Eckfeld floras. Regression lines are from a linear model, and R^2^ values are shown on the plots. Error bars were omitted to reduce clutter.

Damage frequency in fossil floras has been significantly lower than analogous values in modern studies [47]. Nevertheless, the frequency is significantly higher at Messel than at Eckfeld (20.7% vs. 10.9% of leaves damaged). Linear regressions were used to determine the significance of correlation between damage frequency and dicot diversity (Fig. 4). The explained variance (*R* ^2^) of the regression analysis for Messel (R^2^ = 0.80, *p*<10^−12^) and for Eckfeld (R^2^ = 0.59, *p*<10^−3^) is high. Mean damage frequency [48], [49] among plant morphotypes that are represented by >25 specimens constitutes 25% of the leaves at Messel and 16% at Eckfeld, with the range greater in Eckfeld than in Messel. Most of the damaged leaves (86.4% at Messel and 83.1% at Eckfeld) bear only a single type of damage, followed at both localities by ∼ 11% damaged leaves with two types of damage, followed by 2.2% at Messel and 4.4% at Eckfeld that have three types of damage, and less than 1% of leaves exhibiting four types. Neither flora exhibited more than six DTs on a single leaf.

At Messel the six most abundant host-plant taxa accounted for approximately half of the fossil leaves, which were affected by about 51.3% of insect damage occurrences (Table 3). Juglandaceae sp. 1 is the most abundant taxon (12.9%) at Messel and also one of the most highly damaged species (55.4%). The corresponding diversity of 53 DTs is the highest in the entire assemblage. Second in rank order is, *Laurophyllum lanigeroides* (12.7%) representing a considerably lower proportion of damaged leaves (28.5%) with a diversity of 35 DTs. Three other similar taxa, Lauraceae sp. 1 (8.3%), Juglandaceae FK1 (6.8%), and *Laurophyllum* sp. indet. (6.6%), exhibited a similar proportion of damaged leaves (43.6%, 38.6%, and 38%, respectively). However, *Rhodomyrtophyllum sinuatum* (Myrtaceae), representing only 1.3% of the total plant taphocoenosis, had the highest damage frequency, amounting to 56.5% of the damaged leaves and a diversity of 24 DTs. Of the specialized DTs, we evaluated mining and galling. Mining occurs on 3.0% of leaves, which is more than three times the frequency within the Eckfeld sample. Most of these mines are morphologically similar to those made by microlepidopterans and represent minimally the presence of five different miner groups. Galling is most abundant on Lauraceae and Juglandaceae, where 10.1% and 13.5% of the leaves are attacked, respectively. By contrast, the galls are structurally similar to those made by Cecidomyiidae (gall midges) [16], [50].

**Table 3:** Dicot species or morphotypes with at least 50 specimens in the Messel Maar floral community, their assignments to “evergreen” (E), “deciduous ” (D), or unassigned (U) categories for analysis, and their bases of assignment (L), leaf texture and/or other foliar features; (R), phenology of all or most living relatives.

*Species*	*Plant Group*	*Habit*	*Basis*	*# Leaves*	*% dam.*	*# DTs*	*DTs at 50*	*SpecDTs* *at 50*	*MineDTs* *at 50*	*GallDTs* *at 50*
**Juglandaceae sp. 1**	Juglandaceae	E	L,R	710	55.35±1.87	53	12.03±2.50	5.49±1.79	1.24±0.99	3.98±1.50
***Laurophyllum*** ***lanigeroides***	Lauraceae	E	L	698	28.51±1.72	35	8.71±2.18	3.24±1.39	0.55±0.69	3.16±1.24
**Lauraceae sp. 1**	Lauraceae	E	L,R	456	43.64±2.32	40	11.06±2.40	3.91±1.83	0.99±0.85	4.03±1.61
**Juglandaceae FK 1**	Juglandaceae	E	L,R	376	38.56±2.52	33	11.01±2.36	6.76±1.88	1.18±0.88	5.49±1.68
***Laurophyllum*** ** sp.**	Lauraceae	E	L	361	37.95±2.55	30	10.94±2.28	3.51±1.57	0.85±0.77	3.74±1.54
**Leguminosae sp. 5**	Fabaceae	E	L,R	272	31.62±2.83	25	7.89±1.99	2.58±1.33	0.88±0.82	2.29±0.91
**“** ***Ficus*** **” sp.**	Moraceae	E	L,R	196	18.37±2.74	19	7.07±2.18	2.94±1.48	0.95±0.78	2.41±1.18
***Daphnogene crebrigranosa***	Lauraceae	E	L	188	9.04±2.09	7	3.15±1.27	0.79±0.76	0.53±0.62	–
***Daphnogene*** ** sp. 1**	Lauraceae	E	L	173	30.06±3.48	16	7.71±1.63	2.13±1.15	–	2.13±1.14
**Juglandaceae FK 2**	Juglandaceae	D	L,R	123	30.89±4.17	17	9.50±2.00	4.78±1.49	0.81±0.69	2.90±1.09
**Ulmaceae sp.**	Ulmaceae	D	R	92	22.83±4.39	8	5.78±1.11	1.64±0.87	–	–
**Vitaceae sp.**	Vitaceae	U		88	27.27±4.73	19	11.83±2.01	6.75±1.66	3.34±1.22	2.29±0.97
**Dicot. sp. GM 2**	unknown	U		84	4.76±2.38	4	2.37±1.20	1.19±0.69	–	1.19±0.68
***Daphnogene*** ** sp. 1**	Lauraceae	E	L	84	5.95±2.59	5	2.96±1.60	1.19±0.69	0.60±0.49	0.59±0.49
***Tremophyllum*** ** sp.**	Ulmaceae	D	L,R	81	29.63±5.09	14	10.06±1.51	3.92±1.11	1.47±0.60	1.87±0.83
***Laurophyllum hirsutum***	Lauraceae	E	L	77	9.09±3.26	6	4.14±1.15	1.31±0.67	–	0.66±0.47
***Rhodomyrtophyllum sinuatum***	Myrtaceae	E	L,R	69	56.52±5.96	24	18.51±2.06	10.39±1.60	2.18±0.76	5.52±1.00
**Myricaceae sp.**	Myricaceae	U		68	13.24±4.08	9	6.60±1.41	3.63±1.17	2.19±1.00	0.73±0.45
**Ulmoideae FK 1**	Ulmoidea	D	R	54	5.56±3.23	3	2.77±0.59	1.85±0.52	–	0.92±0.27
***Toddalia ovata***	Rutaceae	E	L,R	54	14.81±4.86	6	5.62±0.68	3.78±0.44	0.99±0.05	1.84±0.38
**Nymphaeaceae sp. 1**	Nymphaeaceae	D	L,R	54	20.37±5.44	2	1.93±0.26	1.92±0.26	–	1.93±0.25

Abbreviations: (DT), Damage type; (SpecDT), Specialized Damage type; (MineDT), Mining Damage type; (GallDT), Galling Damage type; all categories are rarefied to 50 specimens.

At Eckfeld, the most frequently damaged taxon belongs to a member of the laurel family (Lauraceae sp. 1), which remarkably has a damage frequency of 75%, corresponding to a diversity of 28 DTs. Lauraceae sp. 1 represents the taxon with the second highest frequency in the assemblage. The next six most abundant host-plant taxa, with more than 100 specimens, account for three-fourths of the fossil leaves, and are affected by approximately 60.4% of insect damage occurrences. Juglandaceae sp. 1 is the most abundant taxon (31.1%) at Eckfeld, of which only 19.9% are damaged but it also has the greatest diversity of damage types (43 DTs). Galling is particularly prominent on Juglandaceae sp. 1, which hosts ten gall DTs. Lauraceae sp. 1 stands out for its high number of mine and gall DTs, after having been standardized by the number of leaves (Table 4). Fabaceae sp. 1 and Dicot sp.-salicoid both have the highest percentage of damaged leaves in the sample, accounting 36.4%, and 17.7%, respectively. These taxa are followed by Dicot. sp., vitioid (11.8%) and a species of the dogbane family (Apocynaceae indet.) with 11.2% damaged leaves. Among abundant plant taxa, Cercidiphyllaceae sp. 1 exhibited only one type of damage (DT2), representing the lowest level (3.8%) of damage diversity in the flora.

Plant species in both localities demonstrate a wide range of damage frequencies, probably reflecting their differing investments in antiherbivore defenses [43], [51], [52]. Using the fossil record from the middle Eocene of Utah, Wilf et al. [15] observed more herbivory on leaves inferred to be short-lived (deciduous plants) than those that are thought to have been long-lived (evergreen plants). Interestingly, evergreen hosts – dominant at both Messel and Eckfeld – bear significantly higher damage percentages than co-occurring deciduous plants; indicated by pairwise Wilcoxon signed rank test results where *p*<0.002 for all two parameters.

### Damage Type Distribution

**Figure 5:**
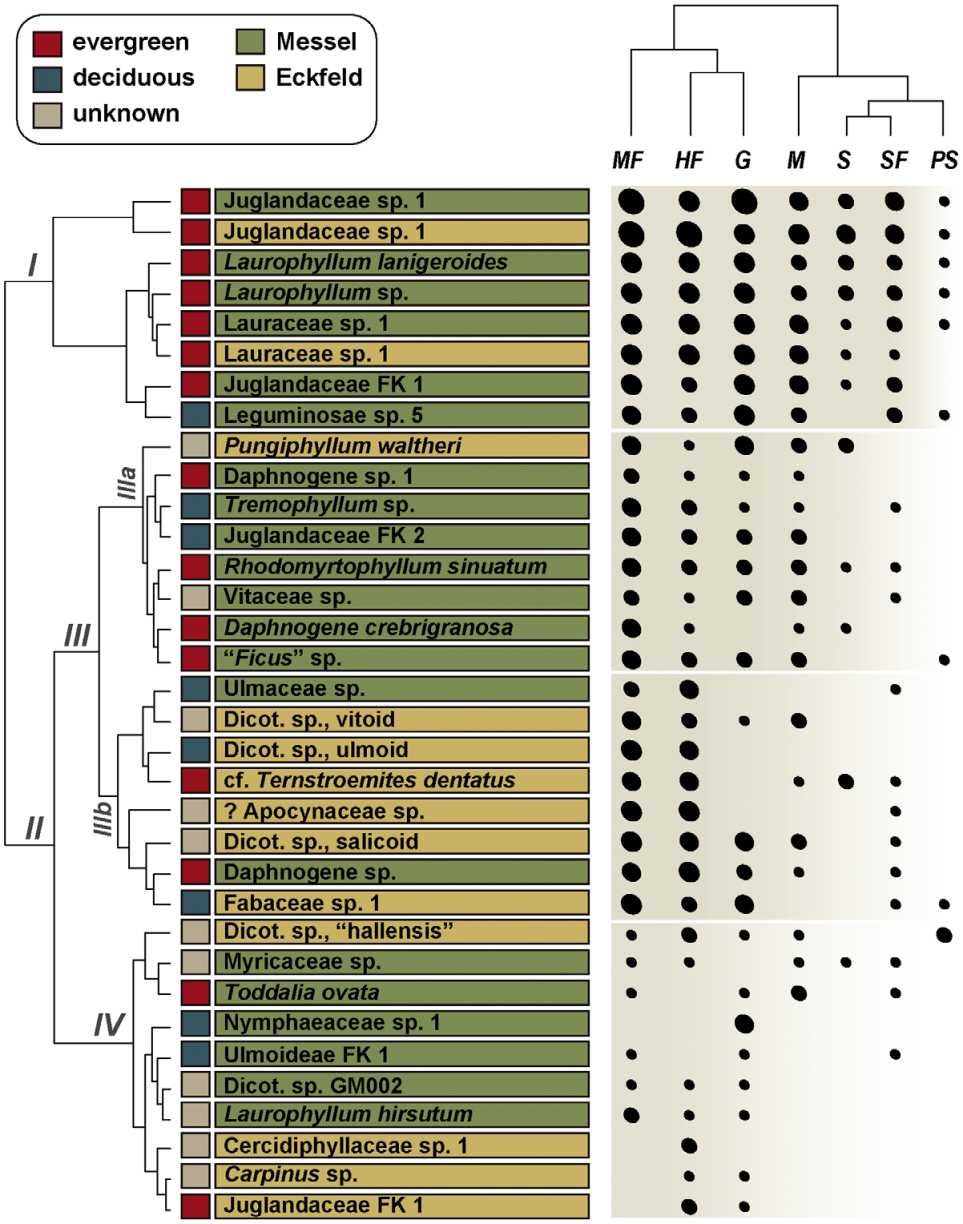
Two-way cluster analysis of insect damage on species–locality pairs, based on relative abundances of the seven functional feeding groups. Each plant from Messel and Eckfeld with at least 50 specimens was included in the analysis. Black circles are scaled according to the relative abundance of each functional feeding group on each plant host. The two-way cluster analysis was performed using the protocol of [11]. The significance of the clusters in Roman numerals is explained in the text. Abbreviations are: MF, margin feeding; HF, hole feeding; G, galling; M, leaf mining; S, skeletonization; SF, surface feeding; and PS, piercing and sucking.

We examined changes in damage-type composition and distribution by performing a two-way cluster analysis on: (1), the 34 plant species–locality pairs with greater than 50 specimens, and (2), the relative abundances of the seven functional feeding groups measured by DT frequencies (Fig. 5). The agglomerative coefficient for the clustering of plant species is 0.96 and for the clustering of functional feeding groups 0.72. The abundances of the more specialized functional feeding groups principally are driven by the high diversity of plant species, and probably several herbivore-related plant attributes based on modern studies of host-specialist insects. These important factors include leaf nutritional quality, leaf toughness, and the presence of a wide variety of secondary metabolites – features associated with elevated herbivore defense in tropical plants [53]. Conspicuously, the number of functional feeding groups present and their relative abundances decrease between Cluster I (at top) and Cluster IV (at bottom). The first split separates the most abundant evergreen taxa that form Cluster 1 from the other clusters. This cluster is associated with all seven functional feeding groups, especially those which are distinguished by high levels of specialized damage. The remaining Cluster II divides further into two major subclusters, labeled as Cluster III and Cluster IV. The majority of host plants in Cluster III are comprised of taxa that show a more or less intermediate abundance of functional feeding groups, but are characterized by a diminution of specialized damage resulting in another split. Cluster IIIa species–locality pairs are dominated by a mixture of Messel taxa showing a moderate abundance of hole feeding, margin feeding, mining, and galling, but also are characterized by poor representation of external foliage feeding groups such as skeletonization or surface feeding. Cluster IIIb is dominated by Eckfeld taxa with abundant hole feeding, margin feeding, and surface feeding, but little skeletonization, and moderate levels of galling on two evergreen hosts (*Daphnogene* sp., Fabaceae sp. 1) and one deciduous host (Dicot. sp.-salicoid). Cluster IV contains taxa that have less damage overall and minimal specialized damage. Notably, two Cluster IV species–locality pairs show the only occurrence of a single DT. Nymphaeaceae sp. 1 is characterized by the presence of a single gall type (DT49), and Cercidiphyllaceae sp. 1 exhibits only a single type of hole feeding (DT02).

**Table 4:** Dicot species or morphotypes with at least 50 specimens in the Eckfeld Maar floral community, their assignments to “evergreen” (E), “deciduous” (D), or unassigned (U) categories for analysis, and their bases of assignment (L), leaf texture and/or other foliar features; (R), phenology of all or most living relatives.

*Species*	*Plant Group*	*Habit*	*Basis*	*# Leaves*	*% dam.*	*# DTs*	*DTs at 50*	*SpecDTs* *at 50*	*MineDTs* *at 50*	*GallDTs* *at 50*
**Juglandaceae sp. 1**	Juglandaceae	E	L,R	1186	19.56±1.15	43	6.56±2.28	2.10±1.29	0.42±0.61	1.31±1.03
**?Apocynaceae sp.**	unknown	U		365	11.23±1.64	10	3.60±1.42	0.27±0.48	–	–
**cf. ** ***Ternstroemites dentatus***	Theaceae	E	L,R	333	5.71±1.30	9	2.39±1.22	0.42±0.57	0.15±0.35	–
***Pungiphyllum waltheri***	unknown	U		331	5.44±1.20	7	2.36±1.25	1.11±0.86	0.28±0.45	0.68±0.65
**Dicot. sp., ulmoid**	unknown	D	L	306	6.21±1.36	7	2.24±1.16	0.31±0.46	–	–
**Dicot. sp., salicoid**	unknown	U		215	17.67±2.62	20	6.30±2.52	2.54±1.44	0.46±0.59	1.59±1.05
***Carpinus*** ** sp.**	Betulaceae	U		112	1.79±1.32	2	0.88±0.99	0.46±0.49	–	0.46±0.49
**Dicot. sp., vitioid**	unknown	U		93	11.83±3.37	9	5.18±2.39	2.08±1.58	1.61±1.10	0.52±0.49
**Lauraceae sp. 1**	Lauraceae	E	L,R	84	75.00±4.72	28	20.09±2.25	9.51±1.61	2.97±1.05	7.07±1.31
**Dicot. sp., “** ***hallensis*** **”**	unknown	U		61	11.48±4.01	5	4.40±0.72	1.65±0.53	0.82±0.38	0.83±0.37
**Juglandaceae FK 1**	Juglandaceae	E	L,R	55	5.45±2.94	3	2.72±0.87	0.90±0.29	–	0.90±0.29
**Fabaceae sp. 1**	Fabaceae	D	L	55	36.36±6.47	10	9.42±0.69	2.80±0.41	–	2.81±0.42
**Cercidiphyllaceae sp. 1**	Cercidiphyllaceae	U		53	3.77±2.69	1	0.99±0.05	–	–	–

(DT), Damage type; (SpecDT), Specialized Damage type; (MineDT), Mining Damage type; (GallDT), Galling Damage type; all categories arerarefied to 50 specimens.

## Discussion

Our and previous work on the European floras from Messel and Eckfeld demonstrate an unexpected high richness of plant–insect associations consisting of varied external foliage feeding, piercing-and-sucking, leaf mining, galling, seed predation and oviposition [54], and incidental interactions from the third trophic level of predators and parasitoids [18]. Collectively, the richness of these associations exceeds that from other greenhouse peaks, such as the PETM or EECO. Previously, similar studies investigating the diversity and intensity of associations of Paleogene plants and plant–insect associations pointed to the strong influence that climate change, in particular transitional intervals of rapid greenhouse warming, had on insect herbivory [10]–[12]. Given the results of these earlier, high-resolution examinations, our new data from Messel and Eckfeld offer an opportunity to analyze censuses and offer comparisons of these two, younger deposits to other sites covering a Paleogene greenhouse climate. Given this context, we note two important plant–insect associational trends.

### Plant–insect Associational Intensity Follows Decreased pCO_2_ and Temperatures

Based on proxy data from deep-sea cores, the early middle Eocene interval, approximately overlapping with the Lutetian Stage (43.3–49 Ma), was an exceptionally warm, ice-free period during the Cenozoic. This period was characterized by greenhouse conditions with atmospheric carbon dioxide levels (*p*CO_2_) ranging from two to three (i.e., *p*CO_2_>800 ppm) times those of Holocene values [24], [55]. In general, the effects of this increase in *p*CO_2_ is the decrease of foliar nitrogen (N) concentration, thus elevating the carbon to nitrogen (C:N) ratio and thereby stimulating increased insect feeding which is necessary to maintain metabolic homeostasis [56]–[59]. Significantly, the herbivory pattern for Messel and Eckfeld demonstrates that overall diversity [60] and intensity [61]of insect herbivore damage parallels that of *p*CO_2_ levels. However, this trend is more evident for Messel than for Eckfeld.

Data for temperature reconstructions from oxygen isotopes in marine foraminifera [6], [62], as well as paleobotanical estimations of terrestrial temperatures for the Cenozoic of Central Europe [13], [63], [64], exhibit a general cooling trend for the Eocene. The estimated seasonal range of temperatures of approximately 13°C for the middle Eocene is within the range of recent values for mid-latitude, near-ocean sites. The Eocene cooling trend was mostly attributable to decreasing winter temperatures whereas at the same time summer temperatures remained rather stable [65]. Temperature fluctuations likely had an impact on plant and insect diversity, which is supported by several studies of extant tropical and subtropical sites showing strong positive correlations between the diversity of plants and those of herbivorous insects [29], [42], [53], [66]–[68]. Therefore, one might expect more varied types of damage, particularly mines and other specialized damage, on a more diverse bulk flora. Our results reinforce these modern observations that plant and insect-herbivore diversity are positively linked and underline the elevated richness of plant–insect associations within the European Eocene ecosystem. Also important for climate reconstructions is the paleogeographic context of localities such as Messel and Eckfeld. Both sites represent isolated, volcanogenic lakes which were surrounded by zonal vegetation. This may have had a greater impact on vegetation composition and diversity than differences in climate [33].

Explanations other than cooling temperatures could account for the decline in the diversity and intensity of plant–insect associations when Messel is compared to Eckfeld. Differences of the plant taphocoenoses in both localities and the obvious decline in interactions among plants and insect herbivores from Messel to Eckfeld could have been caused by soil differences affecting the zonal vegetation. Mature, Lower Devonian clastic sediments surrounded the Eckfeld Maar [69], whereas immature crystalline material and its immediate derivatives of Rotliegend sediments is the country rock adjacent the Messel pit [70]. It is commonly accepted that insect herbivore performance often depends on plant nutrient supply that is immediately related to substrate quality [71]–[73].

Another explanation for the decrease in diversity and intensity of herbivory between the time of deposition at the two localities could involve differences in paleoelevation. Such an assumption is complicated, as altitude constitutes a significant component of climate. Estimates of elevation place Messel approximately at sea level whereas Eckfeld is placed between 355 and 430 m [21]. This suggests some difference in climate between the two sites, if all other environmental variables were held constant. At a regional scale, arthropod species diversity is negatively correlated with altitude [74], [75], observable in both herbivorous arthropods such as caterpillars [76], and in nonherbivorous species such as Scarabaeoidea [77]. All together, the few studies of insect herbivory along altitudinal gradients have so far shown a decrease in herbivore activity with increasing altitude [78].

### Evergreen Hosts are More Intensely Herbivorized than Co-occurring Deciduous Hosts

We have documented a general relationship of atmospheric *p*CO_2_ occurring at ca. 2.5 times the current levels during Messel times [23] that subsequently fell to ca. 2 times of the current levels during Eckfeld times [79], which parallels a decreasing trend of plant–insect associational intensity as documented in Figures 3 and 4. We are aware that a high resolution record of carbon dioxide concentrations throughout the Eocene does not yet exist, making it impossible to quantitatively correlate the partial pressure of atmospheric *p*CO_2_ and herbivory, or to decouple the effects of temperature and *p*CO_2_. The insect-herbivore damage examined by [10] records terrestrial ecosystem response to both temperature and *p*CO_2_, which are coupled in natural systems over long timescales. However, there is a general drop in *p*CO_2_ by Eckfeld time and distinct patterns emerge from our data.

The result we obtained fits the significant predominance of intensely herbivorized evergreen plant hosts forming seven-eighths of the most intensely herbivorized Cluster I, principally represented by Messel plant morphotypes (Fig. 5; Table 5). This trend of elevated herbivory on evergreen taxa rather than on the deciduous component at Messel, and to a lesser extent at Eckfeld, appears to be opposite to a pattern of herbivory that favor deciduous over evergreen taxa with rising temperatures and *p*CO_2_ levels, as previously demonstrated for deposits of the Greater Green River Basin in the United States [10], [12], [15]. At the Greater Green River Basin sites, evergreen taxa have lower levels of herbivory but were attacked by a greater percentage of specialized herbivores [10], [15].

**Table 5:** Generalized versus specialized herbivory for Messel and Eckfeld evergreen and deciduous plant taxa.

Messel				
*generalized*		*specialized*		
873 occurences	(58.94%)	414 occurences	(27.95%)	evergreen plant taxa
137 occurences	(9.25%)	57 occurences	(3.84%)	deciduous plant taxa
**Eckfeld**				
***generalized***		***specialized***		
240 occurences	(61.38%)	92 occurences	(23.52%)	evergreen plant taxa
46 occurences	(12.53%)	10 occurences	(2.55%)	deciduous plant taxa

Although seemingly anomalous, there is significant current evidence that indicate under conditions of rising and elevated *p*CO_2_, the robust, mesophyll-rich leaf anatomy of sclerophyllous evergreen plants allow for more efficient photosynthesis and water use than do deciduous plants possessing a looser arrangement of mesophyll and other foliar tissues involved CO_2_ diffusion [80], [81]. This pattern is particularly buttressed, if a whole-plant approach is taken that encompasses all organs [82], such that greater growth rates and production of photosynthate are accounted for. A response by evergreens under conditions of decreasing *p*CO_2_ levels, such as during the Messel to Eckfeld interval, would have acquired a more diverse and challenging component community of insect herbivores to take advantage of greater efficiencies in evergreen over deciduous plant primary productivity. This appears to be a general pattern, especially for specialized insect lineages with modifiable life-history strategies that exploit plant hosts with particular physiognomies, distinct environmental preferences, and augmented photosynthetic efficiencies [60], [61].

An alternative hypothesis accounting for the pattern of preferential herbivory on Messel and Eckfeld evergreen taxa would be that such arborescent taxa are more abundant and conspicuous, and therefore would be more apparent, to herbivore consumption [83]. The apparency hypothesis, initially developed for woody dicotyledonous angiosperms [83], was also supported by herbivory patterns on herbaceous angiosperms [84], and may be a general phenomenon explaining herbivory intensities [68]. However, for the Messel and Eckfeld floras, a more likely explanation favors increased herbivory based on the leaf anatomy of sclerophyllous taxa [80], [82], suggesting that particular physiological traits of plant hosts are being tracked rather than plant conspicuousness.

In summary, our results show an unexpected high diversity of insect feeding and targeting of evergreen over deciduous plant hosts at Messel and Eckfeld, compared to analogous, contemporaneous floras from North and South America. While a cooling trend, soil type variation, or differences in elevation may explain the significant lessening of herbivory type and intensity at Eckfeld compared to that at Messel, we suspect that a more compelling reason involves an important, previously unknown component of the plant-host and insect-herbivore diversification event during the European Eocene. Additionally, modern plant–insect associational evidence indicates that sclerophyllous evergreen plants are highly herbivorized under elevated *p*CO_2_ conditions. Nevertheless, only a few studies of middle Eocene variation in insect herbivory are available that provide a window into to the impact of changes surrounding the last major greenhouse interval of the Cenozoic. Consequently a comparison of Messel and Eckfeld with other subtropical rainforest biomes in mid-latitudinal regions offers an opportunity to better understand the long-term consequences of climate warming and cooling on ecosystems.

## Materials and Methods

### Geological Overview

The Fossillagerstätten of Messel and Eckfeld are deposits of maar lakes which were formed during the first half of the middle Eocene. Both basins were initially formed by volcanic explosions, resulting in deep, depressions on top of diatremes that were soon occupied by lakes. Following early stages with succeeding volcanoclastic and predominantly siliciclastic sedimentation, the lakes became meromictic and the finely laminated bituminous claystone (“oilshale”) was formed in the quiet anoxic bottom layer of the lake. The oilshale contains remains of microorganisms, aquatic invertebrates, aquatic and terrestrial vertebrates, but the taphocoenosis is dominated by land plants and insects [19]–[22].

Messel is located on the eastern side of the Rhine Rift Valley, about 8 km northeast of Darmstadt. The sediments of the Messel Formation have been biostratigraphically dated as lower middle Eocene (lower Geiseltalian or MP 11 of the European Land Mammal Age chronology (ELMA)) [85], and a radiometric age (Ar^40/39^) of 47.8±0.2 Ma has been obtained from the volcaniclastic sediments [86]. The crater structure containing Lake Messel had a diameter of about 1.5 km and initially a depth of 300–400 m [70]. It was therefore considerably larger than Eckfeld Maar. The fossils represent a diverse biota of exceptionally preserved micro-organisms, leaves, insects, fishes, amphibians, reptiles, birds, and mammals [19], [32], [87], inferred to represent a paratropical Eocene rain forest.

The crater structure at Eckfeld Maar near Manderscheid, in Eifel, Germany, originally had a diameter of 900 m and a depth of about 170 m. The depth of the maar lake initially exceeded 110 m and might have reached 150 m [88]. Rapid sedimentation over a 250.000 year period combined with anoxic alkaline conditions resulted in the absence of bioturbation and explains the perfect preservation of fossils within the oil-shale laminae [89]. Presently more than 30.000 macrofossils have been excavated, all of which document a highly diverse terrestrial flora and fauna representing an ecosystem towards the end of the middle Eocene [21], [90], [91]. Eckfeld is about 3 million years younger than Messel and represents the middle Eocene Mammal Paleogene reference level MP13 (late Geiseltalian) of the European Land Mammal Age chronology (ELMA), which is equivalent to the Middle Lutetian [92]. Argon^40/39^ dating of basalt from the diatreme breccia underlying the lake sediments resulted in an age of 44.3±0.4 Ma at Eckfeld [93].

### Insect Damage Data

In this study, we compare insect-feeding damage on the two middle Eocene floras from Messel and Eckfeld. All specimens of leaves, or leaflets in the case of compound leaves, thought to be from woody, non-monocotyledonous (dicotyledonous) angiosperms were identified to species when possible or alternatively to well-defined morphotypes when taxonomy was ambiguous. Nondicotyledonous plants were not included in the analysis. Specimens from the Messel oil shale are housed at the Senckenberg Forschungsinstitut und Naturmuseum, at Frankfurt am Main, Germany, and the Hessisches Landesmuseum, at Darmstadt, Germany, and are numbered with the prefixes “SMB Me” and “HLMD Me”. All material from Eckfeld is housed in the collections of the Naturhistorisches Museum Mainz/Landessammlung für Naturkunde Rheinland-Pfalz in Mainz, Germany, and numbered with the prefix “PB NHMM.” The individual oil-shale specimens are stored in glycerol to prevent damage resulting from desiccation.

### Data Collection

We analyzed 16.082 angiosperm leaves in total (Table 1) and scored each specimen for the presence or absence of 89 distinctive and diagnosable insect DTs found in the total data set. Both sites represent deposition in similar meromictic lake environments, and the leaf assemblages are autochthonous to parautochthonous. Fossil leaves and their insect damage were quantitatively censused from single stratigraphic horizons at each site (microstratigraphical excavation method) [19], [21], [94]. During the excavations the stratigraphic positions of all fossils were determined as distances to the nearest by marker horizon. In doing the quantitative evaluation, we were well aware of a bias caused by taphonomical processes. At Messel, shallow lake-margin shores were covered by a herbaceous vegetation [35], acting as an effective taphonomic baffle for entrapment. By contrast, Eckfeld was characterized by comparatively steeper and unstable slopes [94]. Consequently more incomplete plants generally were preserved in the Eckfeld material. To underscore a potential criticism of inflated richness in the European sites, we note that the richest EECO lacustrine leaf flora known from South America is the early Eocene Laguna del Hunco flora [30]. When sampled using very similar collecting methods, Laguna del Hunco yielded an equal number of 75 dicot-leaf species from approximately 1000 specimens, equivalent to 75.61 species when rarefied to the sample size of Messel and a much lower total of 31.71 dicot-leaf species in Eckfeld.

The damage types (DTs) have been assigned to five main functional feeding groups and subgroups of external foliage feeding, galling, mining, oviposition, and piercing-and-sucking [54]. At both localities the spectrum of interactions included a diverse repertoire of scale-leaf scars indicating piercing-and-sucking, midrib and foliar galls, and serpentine to blotch mines. Additionally, leaf-margin excisions, hole feeding, skeletonization, bud feeding, and ovipositional damage were recorded (Fig. 1 and File S3). Proxy criteria were used to infer whether well sampled plant species more likely bore short- or long-lived leaves. The categorizations were based on the combined, available evidence from leaf texture [33], [35], leaf-margin state, inference from living relatives, and estimated leaf mass per area, using the method of Royer et al. [36]. The procedure for collecting and evaluating plant–insect associational data is derived from an explicit classification of insect-mediated damage [95]. This spectrum of damage types, or DTs [95], [96], is based on the response of insect feeding on live plant tissues, using four diagnostic criteria [97]. The presence or absence and type of DT data are tabulated for each leaf per locality, together with host-plant identification and relevant commentary (see Files S1 and S2).

### Quantitative Analyses

Quantitative analyses of insect damage occurrences and diversity were performed in R version 2.10.0 (R Development Core Team, Vienna, Austria). The differences among the proportions of occurrence of individual functional feeding groups (FFG) were analyzed by χ^2^ test. The remaining analyses were done by using the generalized linear models (GLM) of the binomial family of distributions. The percentage of explained variability was computed by means of Nagelkerke pseudo-R^2^ measure as implemented in the Design 2.3-0 R-package [98], [99]. Where necessary, overdispersion was treated by refitting to the quasibinomial family of GLM and subsequent use of F tests at appropriate places [100]. In general, insect folivory was examined using three damage-type metrics: frequency, diversity, and distribution. Damage diversity, or the number of DTs present at both localities or on host species, was normalized for the number of leaves sampled as in previous studies [12], [48], [49].

## Supporting Information

File S1 **Complete Messel insect damage dataset.**
(PDF)

File S2 **Complete Eckfeld insect damage dataset.**
(PDF)

File S3 **New damage types (DTs).**
(PDF)
