# Supplementary material for: Testing for the Effects and Consequences of Mid Paleogene Climate Change on Insect Herbivory
Source: PLoS One. 2012 Jul 18;7(7):e40744. doi: 10.1371/journal.pone.0040744 (PMC3399891; doi:10.1371/journal.pone.0040744)
Supplement: File S1 — Complete Messel insect damage dataset. (PDF) [file pone.0040744.s001.pdf]

Testing for the Effects and Consequences of Mid Paleogene Climate Change on Insect Herbivory

Torsten Wappler[1*], Conrad C. Labandeira[2,3], Jes Rust[1], Herbert Frankenhäuser[4], Volker Wilde[5]

1 Steinmann Institute, University of Bonn, 53115 Bonn, Germany; **2** Department of Paleobiology, National Museum of Natural History, Smithsonian Institution, Washington, DC 20013, USA; **3** Department of Entomology and BEES Program, University of Maryland, College Park, MD 20742, USA; **4** Mainz Natural History Museum / State Collection for Natural History of Rhineland-Palatine, Mainz, Germany; **5** Senckenberg Forschungsinstitut und Naturmuseum, Paläobotanik, Frankfurt am Main, Germany.

***** Author for correspondence.

ELECTRONIC SUPPLEMENTARY MATERIAL

FILE S1

Dataset 1. Summary data for the Messel maar locality. The number of occurrences of each damage type (DT) was recorded.

Collection number	Leaf species or morphotype	Damage type summary	DT 1	DT 2	DT 3	DT 4	DT 5
HLMD_Be_26	Lauraceae sp.	2 12	0	1	0	0	0
HLMD_Be_27	Dicot. sp. indet.	15	0	0	0	0	0
HLMD_Be_30	Dicot. sp. indet.	12 29	0	0	0	0	0
HLMD_Be_37	Lauraceae sp.	176	0	0	0	0	0
HLMD_Be_41	Juglandaceae sp.	2	0	1	0	0	0
HLMD_Be_45	Dicot. sp. indet.	3	0	0	1	0	0
HLMD_Be_47	Dicot. sp. indet.	201 12	0	0	0	0	0
HLMD_Be_52	Lauraceae sp.	30 2	0	1	0	0	0
HLMD_Be_54	Lauraceae sp.	30	0	0	0	0	0
HLMD_Be_58	Leguminosae sp. 5	147	0	0	0	0	0
[illegible]	Leguminosae sp. 5	30	0	0	0	0	0
HLMD_Be_61	Lauraceae sp.	33	0	0	0	0	0
HLMD_Be_65	Lauraceae sp.	109	0	0	0	0	0
HLMD_Me_10170	Lauraceae sp.	54	0	0	0	0	0
HLMD_Me_10173	Vitaceae sp.	3	0	0	1	0	0
HLMD_Me_10192	Juglandaceae Formenkreis 1	223	0	0	0	0	0
HLMD_Me_10199	Lauraceae sp.	12	0	0	0	0	0
HLMD_Me_10221	Dicot. sp. indet.	216	0	0	0	0	0
HLMD_Me_10228	Juglandaceae Formenkreis 4	220	0	0	0	0	0
HLMD_Me_10233	Juglandaceae sp.	163	0	0	0	0	0
HLMD_Me_10243	Juglandaceae sp.	189	0	0	0	0	0
HLMD_Me_10244	Dicot. sp. GM015	3	0	0	1	0	0
HLMD_Me_10250	Vitaceae sp.	15	0	0	0	0	0
HLMD_Me_10252	Dicot. sp. indet.	43	0	0	0	0	0
HLMD_Me_10403	Juglandaceae sp.	1	1	0	0	0	0
HLMD_Me_10604	Toddalia ovata	12 15	0	0	0	0	0
HLMD_Me_10623	Dicot. sp. indet.	2	0	1	0	0	0
HLMD_Me_10668	Dicot. sp. indet.	36	0	0	0	0	0
HLMD_Me_10707	Lauraceae sp.	206	0	0	0	0	0
HLMD_Me_10709	Lauraceae sp.	12	0	0	0	0	0
HLMD_Me_10721	Juglandaceae sp.	201	0	0	0	0	0
HLMD_Me_10727	Daphnogene sp. 2	54 12 30	0	0	0	0	0
HLMD_Me_10737	Laurophyllum sp.	198	0	0	0	0	0
HLMD_Me_10743	Juglandaceae Formenkreis 1	14	0	0	0	0	0
HLMD_Me_10776	Dicot. sp. indet.	207 198	0	0	0	0	0
HLMD_Me_10791	Daphnogene sp.	12	0	0	0	0	0
HLMD_Me_10795	Juglandaceae sp.	3	0	0	1	0	0
HLMD_Me_10808	Lauraceae sp.	12	0	0	0	0	0
HLMD_Me_10854	Tremophyllum sp.	146	0	0	0	0	0
HLMD_Me_10855	Dicot. sp. indet.	208	0	0	0	0	0
HLMD_Me_10856	Dicot. sp. indet.	8 222	0	0	0	0	0
HLMD_Me_10859	Juglandaceae Formenkreis 1	12	0	0	0	0	0
HLMD_Me_10865	Dicot. sp. indet.	12	0	0	0	0	0
HLMD_Me_10870	Laurophyllum sp.	30	0	0	0	0	0
HLMD_Me_10907	Dicot. sp. indet.	145	0	0	0	0	0
HLMD_Me_10911	Leguminosae sp. 5	208	0	0	0	0	0
HLMD_Me_10981	Laurophyllum sp.	171	0	0	0	0	0
HLMD_Me_12035	Vitaceae sp.	32	0	0	0	0	0
HLMD_Me_12040	Juglandaceae sp.	146 12	0	0	0	0	0
HLMD_Me_12083	? Anonaceae sp.	16 12 13	0	0	0	0	0
HLMD_Me_12140	Dicot. sp. indet.	142	0	0	0	0	0
HLMD_Me_12147	Tremophyllum sp.	142	0	0	0	0	0
HLMD_Me_12148	Dicot. sp. GM014	216 5 12	0	0	0	0	1
HLMD_Me_12152	Laurophyllum sp.	17 2	0	1	0	0	0
HLMD_Me_12156	Juglandaceae sp.	62	0	0	0	0	0
HLMD_Me_12158	Dicot. sp. indet.	195	0	0	0	0	0
HLMD_Me_12173	Acer sp.	12	0	0	0	0	0
HLMD_Me_12243	Laurophyllum lanigeroides	206	0	0	0	0	0
HLMD_Me_12255	Juglandaceae Formenkreis 1	163	0	0	0	0	0
HLMD_Me_12265	Ternstroemites sp.	19 15	0	0	0	0	0
HLMD_Me_12271	Ternstroemites sp.	109 3	0	0	1	0	0
HLMD_Me_12272	Laurophyllum lanigeroides	206	0	0	0	0	0
HLMD_Me_12273	Laurophyllum lanigeroides	3	0	0	1	0	0
HLMD_Me_12281	? Ulmoideae Formenkreis 1	163 25	0	0	0	0	0
HLMD_Me_12459	Leguminosae sp. 5	3	0	0	1	0	0
HLMD_Me_12580	Lauraceae sp.	2	0	1	0	0	0
HLMD_Me_13341	Juglandaceae Formenkreis 2	171	0	0	0	0	0
HLMD_Me_13342	Vitaceae sp.	2	0	1	0	0	0
HLMD_Me_13617	? Illiciaceae sp.	222 12	0	0	0	0	0
HLMD_Me_13623	Dicot. sp. GM003	208	0	0	0	0	0
HLMD_Me_13624	Daphnogene crebrigranosa	22	0	0	0	0	0
HLMD_Me_13625	Juglandaceae sp.	17	0	0	0	0	0
HLMD_Me_13626	Daphnogene sp.	2 12 14	0	1	0	0	0
HLMD_Me_13627	Juglandaceae sp.	81	0	0	0	0	0
HLMD_Me_13628	Dicot. sp. GM014	12	0	0	0	0	0
HLMD_Me_13629	Dicot. sp. indet.	189	0	0	0	0	0
HLMD_Me_13630	Vitaceae sp.	30	0	0	0	0	0
HLMD_Me_13631	Laurophyllum sp.	206	0	0	0	0	0
HLMD_Me_13633	Dicot. sp. indet.	38	0	0	0	0	0
HLMD_Me_13634	Dicot. sp. indet.	210	0	0	0	0	0
HLMD_Me_13635	cf. Apocynophyllum helveticum	8	0	0	0	0	0
HLMD_Me_13636	Dicot. sp. indet.	30	0	0	0	0	0
HLMD_Me_13637	Daphnogene sp.	62	0	0	0	0	0
HLMD_Me_13639	Laurophyllum lanigeroides	12	0	0	0	0	0
HLMD_Me_14606	Laurophyllum lanigeroides	210 2	0	1	0	0	0
HLMD_Me_14847	cf. Apocynophyllum helveticum	171 12	0	0	0	0	0
HLMD_Me_14850	? Fagaceae / ?Rhamnaceae sp.	68	0	0	0	0	0
HLMD_Me_14860	Dicot. sp. indet.	210	0	0	0	0	0
HLMD_Me_14871	Ficus sp.	12 2	0	1	0	0	0
HLMD_Me_14883	Juglandaceae sp.	187	0	0	0	0	0
HLMD_Me_14884	Dicot. sp. indet.	2	0	1	0	0	0
HLMD_Me_14895	Dicot. sp. indet.	15 3	0	0	1	0	0
HLMD_Me_14904	Ficus sp.	7	0	0	0	0	0
HLMD_Me_14911	Dicot. sp. indet.	171 41	0	0	0	0	0
HLMD_Me_14916	Juglandaceae Formenkreis 1	208	0	0	0	0	0
HLMD_Me_15523	? Anonaceae sp.	80 211	0	0	0	0	0
HLMD_Me_15792	Dicot. sp. indet.	188	0	0	0	0	0
HLMD_Me_15897	Leguminosae sp. 5	12 29	0	0	0	0	0
HLMD_Me_15929	Lauraceae sp.	12	0	0	0	0	0
HLMD_Me_15930	Dicot. sp. GM014	2	0	1	0	0	0
HLMD_Me_15932	Laurophyllum lanigeroides	12 214	0	0	0	0	0
HLMD_Me_15940	Juglandaceae Formenkreis 4	17	0	0	0	0	0
HLMD_Me_15942	Dicot. sp. indet.	208	0	0	0	0	0
HLMD_Me_16135	Dicot. sp. indet.	12	0	0	0	0	0
HLMD_Me_16174	Dicot. sp. indet.	22	0	0	0	0	0
HLMD_Me_16183	Lauraceae sp.	14	0	0	0	0	0
HLMD_Me_16186	Dicot. sp. indet.	30	0	0	0	0	0
HLMD_Me_16191	Dicot. sp. indet.	2	0	1	0	0	0
HLMD_Me_16195	Juglandaceae Formenkreis 1	117 14	0	0	0	0	0
HLMD_Me_16198	Dicot. sp. indet.	76	0	0	0	0	0
HLMD_Me_16256	Nymphaeaceae sp.	190	0	0	0	0	0
HLMD_Me_16270	Dicot. sp. indet.	8	0	0	0	0	0
HLMD_Me_16342	Dicot. sp. indet.	36	0	0	0	0	0
HLMD_Me_16346	Dicot. sp. indet.	68	0	0	0	0	0
HLMD_Me_16357	Juglandaceae sp.	1	1	0	0	0	0
HLMD_Me_16390	Dicot. sp. indet.	15	0	0	0	0	0
HLMD_Me_16393	Lauraceae sp.	1	1	0	0	0	0
HLMD_Me_16401	Dicot. sp. indet.	12	0	0	0	0	0
HLMD_Me_16403	Dicot. sp. GM014	12 1	1	0	0	0	0
HLMD_Me_16404	Laurophyllum lanigeroides	13	0	0	0	0	0
HLMD_Me_16411	Juglandaceae sp.	149 12	0	0	0	0	0
HLMD_Me_16422	Laurophyllum sp.	12	0	0	0	0	0
HLMD_Me_16502	Ternstroemites engelhardtii	80	0	0	0	0	0
HLMD_Me_16508	Lauraceae sp.	213	0	0	0	0	0
HLMD_Me_16555	Dicot. sp. indet.	2	0	1	0	0	0
HLMD_Me_16570	Lauraceae sp.	148 12	0	0	0	0	0
HLMD_Me_16650	Dicot. sp. indet.	54	0	0	0	0	0
HLMD_Me_1686	Leguminosae sp. 5	208	0	0	0	0	0
HLMD_Me_16978	Laurophyllum lanigeroides	150	0	0	0	0	0
HLMD_Me_1777	Dicot. sp. indet.	12	0	0	0	0	0
HLMD_Me_181	Dicot. sp. indet.	109 2	0	1	0	0	0
HLMD_Me_1948	Dicot. sp. indet.	2 12	0	1	0	0	0
HLMD_Me_2008	Daphnogene sp.	3	0	0	1	0	0
HLMD_Me_2049	Celastraceae sp.	17 2 203	0	1	0	0	0
HLMD_Me_2076	Dicot. sp. indet.	12	0	0	0	0	0
HLMD_Me_3484	cf. Carya bilinica	6	0	0	0	0	0
HLMD_Me_353	Lauraceae sp.	80	0	0	0	0	0
HLMD_Me_354	Laurophyllum sp.	30	0	0	0	0	0
HLMD_Me_355	Dicot. sp. indet.	8	0	0	0	0	0
HLMD_Me_3554	Acer sp.	185	0	0	0	0	0
HLMD_Me_3612	Juglandaceae Formenkreis 1	223	0	0	0	0	0
HLMD_Me_3615	Dicot. sp. indet.	36	0	0	0	0	0
HLMD_Me_3618	Dicot. sp. indet.	2	0	1	0	0	0
HLMD_Me_3619	Dicot. sp. indet.	2	0	1	0	0	0
HLMD_Me_3628	Dicot. sp. indet.	3	0	0	1	0	0
HLMD_Me_3648	Dicot. sp. indet.	3	0	0	1	0	0
HLMD_Me_3650	Laurophyllum sp.	206 12	0	0	0	0	0
HLMD_Me_3677	Laurophyllum sp.	206	0	0	0	0	0
HLMD_Me_415	Dicot. sp. indet.	36 2	0	1	0	0	0
HLMD_Me_448	Dicot. sp. indet.	36	0	0	0	0	0
HLMD_Me_5008	Laurophyllum sp.	30	0	0	0	0	0
HLMD_Me_5463	Lauraceae sp.	45	0	0	0	0	0
HLMD_Me_7197	Dicot. sp. indet.	2	0	1	0	0	0
HLMD_Me_7239	Dicot. sp. indet.	216	0	0	0	0	0
HLMD_Me_7248	Juglandaceae Formenkreis 4	216	0	0	0	0	0
HLMD_Me_7264	Dicot. sp. indet.	12	0	0	0	0	0
HLMD_Me_7265	Dicot. sp. indet.	2	0	1	0	0	0
HLMD_Me_7282	Juglandaceae Formenkreis 2	3	0	0	1	0	0
HLMD_Me_7311	Dicot. sp. indet.	2	0	1	0	0	0
HLMD_Me_7361	Juglandaceae Formenkreis 1	2	0	1	0	0	0
HLMD_Me_7369	Laurophyllum sp.	216	0	0	0	0	0
HLMD_Me_7374	Dicot. sp. indet.	210	0	0	0	0	0
HLMD_Me_7385	Laurophyllum sp.	36	0	0	0	0	0
HLMD_Me_7408	Ficus sp.	208	0	0	0	0	0
HLMD_Me_7531	Laurophyllum hirsutum	2	0	1	0	0	0
HLMD_Me_8009	Dicot. sp. indet.	54	0	0	0	0	0
HLMD_Me_8243	Dicot. sp. indet.	30	0	0	0	0	0
HLMD_Me_8267	Laurophyllum sp.	81	0	0	0	0	0
HLMD_Me_8278	Leguminosae sp. 5	171	0	0	0	0	0
HLMD_Me_8280	Dicot. sp. indet.	220 8	0	0	0	0	0
HLMD_Me_8405	Dicot. sp. indet.	2	0	1	0	0	0
HLMD_Me_8412	Dicot. sp. indet.	12	0	0	0	0	0
HLMD_Me_8588	Laurophyllum sp.	206	0	0	0	0	0
HLMD_Me_8591	Nymphaeaceae sp.	49	0	0	0	0	0
HLMD_Me_8598	Laurophyllum sp.	216	0	0	0	0	0
HLMD_Me_8774	Dicot. sp. indet.	2	0	1	0	0	0
HLMD_Me_9028	Dicot. sp. indet.	2	0	1	0	0	0
HLMD_Me_9149	Dicot. sp. GM003	208	0	0	0	0	0
SM.B_Me_1	Dicot. sp. indet.	3	0	0	1	0	0
SM.B_Me_10010	? Illiciaceae sp.	12	0	0	0	0	0
SM.B_Me_10011	Juglandaceae Formenkreis 2	12	0	0	0	0	0
SM.B_Me_10025	Laurophyllum sp.	12	0	0	0	0	0
SM.B_Me_10027	Juglandaceae Formenkreis 1	12	0	0	0	0	0
SM.B_Me_10035	cf. Apocynophyllum helveticum	208	0	0	0	0	0
SM.B_Me_10045	Vitaceae sp.	14	0	0	0	0	0
SM.B_Me_10046	Rhodomyrtophyllum sinuatum	208	0	0	0	0	0
SM.B_Me_10054	Daphnogene sp.	208	0	0	0	0	0
SM.B_Me_10057	Dicot. sp. indet.	12	0	0	0	0	0
SM.B_Me_10065	Lauraceae sp.	3	0	0	1	0	0
SM.B_Me_10068	Dicot. sp. GM003	195 14 2	0	1	0	0	0
SM.B_Me_10069	Lauraceae sp.	216	0	0	0	0	0
SM.B_Me_10077	Lauraceae sp.	14	0	0	0	0	0
SM.B_Me_10080	Lauraceae sp.	3	0	0	1	0	0
SM.B_Me_10081	Laurophyllum sp.	14	0	0	0	0	0
SM.B_Me_10090	Lauraceae sp.	3	0	0	1	0	0
SM.B_Me_10093	Juglandaceae Formenkreis 1	211 195	0	0	0	0	0
SM.B_Me_10095	Laurophyllum lanigeroides	12	0	0	0	0	0
SM.B_Me_10105	Dicot. sp. GM001	32	0	0	0	0	0
SM.B_Me_10119	Leguminosae sp. 5	12	0	0	0	0	0
SM.B_Me_10132	Juglandaceae Formenkreis 1	145	0	0	0	0	0
SM.B_Me_10144	Dicot. sp. GM001	32	0	0	0	0	0
SM.B_Me_10147	Lauraceae sp.	3	0	0	1	0	0
SM.B_Me_10149	Lauraceae sp.	13	0	0	0	0	0
SM.B_Me_10151	Dicot. sp. indet.	195	0	0	0	0	0
SM.B_Me_10165	Juglandaceae sp.	3 30 12	0	0	1	0	0
SM.B_Me_10166	Dicot. sp. indet.	12	0	0	0	0	0
SM.B_Me_10167	Dicot. sp. indet.	212	0	0	0	0	0
SM.B_Me_10171	Dicot. sp. GM003	208	0	0	0	0	0
SM.B_Me_10172	Dicot. sp. GM003	208	0	0	0	0	0
SM.B_Me_10185	Dicot. sp. indet.	43	0	0	0	0	0
SM.B_Me_10187	Dicot. sp. indet.	12	0	0	0	0	0
SM.B_Me_10198	Ternstroemites sp.	208	0	0	0	0	0
SM.B_Me_10210	Juglandaceae Formenkreis 2	76	0	0	0	0	0
SM.B_Me_10215	Laurophyllum sp.	12	0	0	0	0	0
SM.B_Me_10219	Lauraceae sp.	163	0	0	0	0	0
SM.B_Me_10220	Dicot. sp. indet.	12	0	0	0	0	0
SM.B_Me_10226	Lauraceae sp.	3	0	0	1	0	0
SM.B_Me_10235	Dicot. sp. indet.	208 36	0	0	0	0	0
SM.B_Me_10236	Daphnogene sp.	13	0	0	0	0	0
SM.B_Me_10244	Dicot. sp. indet.	3	0	0	1	0	0
SM.B_Me_10245	Lauraceae sp.	3	0	0	1	0	0
SM.B_Me_10248	Daphnogene sp. 1	209 12 4	0	0	0	1	0
SM.B_Me_10261	Lauraceae sp.	3	0	0	1	0	0
SM.B_Me_10263	? Illiciaceae sp.	12	0	0	0	0	0
SM.B_Me_10265	Juglandaceae sp.	3	0	0	1	0	0
SM.B_Me_10341	Nymphaeaceae sp.	49	0	0	0	0	0
SM.B_Me_10345	Dicot. sp. GM003	208	0	0	0	0	0
SM.B_Me_10355	Vitaceae sp.	34	0	0	0	0	0
SM.B_Me_10359	Dicot. sp. indet.	3	0	0	1	0	0
SM.B_Me_10374	Dicot. sp. indet.	208	0	0	0	0	0
SM.B_Me_10405	Daphnogene sp. 1	171	0	0	0	0	0
SM.B_Me_10415	Daphnogene crebrigranosa	185	0	0	0	0	0
SM.B_Me_10699	Daphnogene crebrigranosa	12	0	0	0	0	0
SM.B_Me_107	Daphnogene sp.	32	0	0	0	0	0
SM.B_Me_10713	Laurophyllum sp.	3	0	0	1	0	0
SM.B_Me_10719	Juglandaceae Formenkreis 2	14	0	0	0	0	0
SM.B_Me_10726	Juglandaceae sp.	12	0	0	0	0	0
SM.B_Me_10765	Myricaceae sp.	208	0	0	0	0	0
SM.B_Me_10770	Juglandaceae Formenkreis 1	2	0	1	0	0	0
SM.B_Me_10774	Dicot. sp. indet.	12	0	0	0	0	0
SM.B_Me_10784	Dicot. sp. indet.	19	0	0	0	0	0
SM.B_Me_10787	Dicot. sp. indet.	13	0	0	0	0	0
SM.B_Me_10788	Dicot. sp. indet.	2	0	1	0	0	0
SM.B_Me_108	Laurophyllum sp.	12	0	0	0	0	0
SM.B_Me_10800	Dicot. sp. indet.	12	0	0	0	0	0
SM.B_Me_10803	Rhodomyrtophyllum sinuatum	12	0	0	0	0	0
SM.B_Me_10807	Juglandaceae Formenkreis 2	12	0	0	0	0	0
SM.B_Me_10817	Juglandaceae Formenkreis 1	189	0	0	0	0	0
SM.B_Me_10822	Laurophyllum sp.	12	0	0	0	0	0
SM.B_Me_10842	Laurophyllum sp.	6	0	0	0	0	0
SM.B_Me_10860	Lauraceae sp.	145	0	0	0	0	0
SM.B_Me_10877	Juglandaceae Formenkreis 1	223	0	0	0	0	0
SM.B_Me_10888	Lauraceae sp.	13	0	0	0	0	0
SM.B_Me_11023	Dicot. sp. indet.	171	0	0	0	0	0
SM.B_Me_11039	Dicot. sp. indet.	14	0	0	0	0	0
SM.B_Me_11046	Dicot. sp. indet.	12	0	0	0	0	0
SM.B_Me_11051	Dicot. sp. indet.	2	0	1	0	0	0
SM.B_Me_11053	Lauraceae sp.	145	0	0	0	0	0
SM.B_Me_11058	Dicot. sp. indet.	3	0	0	1	0	0
SM.B_Me_11062	Dicot. sp. indet.	3	0	0	1	0	0
SM.B_Me_11065	Juglandaceae sp.	189	0	0	0	0	0
SM.B_Me_11067	Dicot. sp. indet.	12	0	0	0	0	0
SM.B_Me_11074	Dicot. sp. indet.	210	0	0	0	0	0
SM.B_Me_11082	Juglandaceae sp.	2	0	1	0	0	0
SM.B_Me_11095	Leguminosae sp. 5	2	0	1	0	0	0
SM.B_Me_11100	Laurophyllum sp.	3 12	0	0	1	0	0
SM.B_Me_11113	Rhodomyrtophyllum sinuatum	2	0	1	0	0	0
SM.B_Me_11115	Dicot. sp. indet.	2	0	1	0	0	0
SM.B_Me_11131	Laurophyllum sp.	12	0	0	0	0	0
SM.B_Me_11334	Dicot. sp. GM003	171	0	0	0	0	0
SM.B_Me_11343	Daphnogene sp.	12	0	0	0	0	0
SM.B_Me_11344	Juglandaceae sp.	2 12	0	1	0	0	0
SM.B_Me_11353	Laurophyllum lanigeroides	3 12	0	0	1	0	0
SM.B_Me_11356	Dicot. sp. indet.	2	0	1	0	0	0
SM.B_Me_11357	Ulmaceae sp.	68	0	0	0	0	0
SM.B_Me_11361	Laurophyllum lanigeroides	2	0	1	0	0	0
SM.B_Me_11362	Dicot. sp. indet.	2	0	1	0	0	0
SM.B_Me_11368	Juglandaceae sp.	13	0	0	0	0	0
SM.B_Me_11381	Juglandaceae Formenkreis 2	36	0	0	0	0	0
SM.B_Me_11385	Juglandaceae Formenkreis 2	163	0	0	0	0	0
SM.B_Me_11387	Dicot. sp. indet.	2	0	1	0	0	0
SM.B_Me_11403	Vitaceae sp.	57	0	0	0	0	0
SM.B_Me_11414	Dicot. sp. indet.	200	0	0	0	0	0
SM.B_Me_11416	Leguminosae sp. 5	208 12	0	0	0	0	0
SM.B_Me_11418	Juglandaceae Formenkreis 1	190 36	0	0	0	0	0
SM.B_Me_11430	Juglandaceae sp.	208	0	0	0	0	0
SM.B_Me_11432	Juglandaceae Formenkreis 1	14	0	0	0	0	0
SM.B_Me_11433	Juglandaceae sp.	2	0	1	0	0	0
SM.B_Me_11436	Juglandaceae Formenkreis 1	12	0	0	0	0	0
SM.B_Me_11440	Dicot. sp. GM005	32	0	0	0	0	0
SM.B_Me_11444	Dicot. sp. indet.	12	0	0	0	0	0
SM.B_Me_11471	Dicot. sp. indet.	171	0	0	0	0	0
SM.B_Me_11475	Juglandaceae sp.	12	0	0	0	0	0
SM.B_Me_11483	Daphnogene sp.	12	0	0	0	0	0
SM.B_Me_11488	Laurophyllum sp.	12	0	0	0	0	0
SM.B_Me_11490	Daphnogene sp.	2	0	1	0	0	0
SM.B_Me_11498	Juglandaceae Formenkreis 4	76	0	0	0	0	0
SM.B_Me_11499	Laurophyllum sp.	146	0	0	0	0	0
SM.B_Me_11500	Laurophyllum sp.	3	0	0	1	0	0
SM.B_Me_11502	Dicot. sp. indet.	195	0	0	0	0	0
SM.B_Me_11518	Laurophyllum sp.	12	0	0	0	0	0
SM.B_Me_11520	Juglandaceae sp.	163	0	0	0	0	0
SM.B_Me_11526	Leguminosae sp. 5	147	0	0	0	0	0
SM.B_Me_11545	Laurophyllum sp.	146	0	0	0	0	0
SM.B_Me_11550	Laurophyllum lanigeroides	145	0	0	0	0	0
SM.B_Me_11558	Myricaceae sp.	19	0	0	0	0	0
SM.B_Me_11569	Dicot. sp. indet.	12	0	0	0	0	0
SM.B_Me_11578	Rutaceae sp.	12	0	0	0	0	0
SM.B_Me_11581	Dicot. sp. indet.	187 208	0	0	0	0	0
SM.B_Me_11584	Laurophyllum sp.	146	0	0	0	0	0
SM.B_Me_11593	Juglandaceae sp.	12	0	0	0	0	0
SM.B_Me_11595	Leguminosae sp. 5	147	0	0	0	0	0
SM.B_Me_11597	Dicot. sp. indet.	12	0	0	0	0	0
SM.B_Me_11598	Laurophyllum sp.	17	0	0	0	0	0
SM.B_Me_11602	Ulmaceae sp.	2	0	1	0	0	0
SM.B_Me_11610	Juglandaceae sp.	17 2	0	1	0	0	0
SM.B_Me_11612	Dicot. sp. indet.	12	0	0	0	0	0
SM.B_Me_11618	Juglandaceae sp.	163	0	0	0	0	0
SM.B_Me_11621	Juglandaceae sp.	195	0	0	0	0	0
SM.B_Me_11644	Juglandaceae sp.	12	0	0	0	0	0
SM.B_Me_11651	Juglandaceae sp.	201	0	0	0	0	0
SM.B_Me_11652	Toddalia ovata	171	0	0	0	0	0
SM.B_Me_11660	Dicot. sp. indet.	80	0	0	0	0	0
SM.B_Me_11661	Juglandaceae sp.	12	0	0	0	0	0
SM.B_Me_11671	Juglandaceae sp.	187	0	0	0	0	0
SM.B_Me_11675	Laurophyllum sp.	3	0	0	1	0	0
SM.B_Me_11683	Dicot. sp. indet.	188	0	0	0	0	0
SM.B_Me_11696	Dicot. sp. indet.	187	0	0	0	0	0
SM.B_Me_11708	Dicot. sp. indet.	195	0	0	0	0	0
SM.B_Me_11717	Leguminosae sp. 5	147	0	0	0	0	0
SM.B_Me_11793	Dicot. sp. GM009	19	0	0	0	0	0
SM.B_Me_11794	Dicot. sp. indet.	12	0	0	0	0	0
SM.B_Me_11795	Vitaceae sp.	12	0	0	0	0	0
SM.B_Me_11796	Laurophyllum sp.	3	0	0	1	0	0
SM.B_Me_11797	Vitaceae sp.	36	0	0	0	0	0
SM.B_Me_11809	Laurophyllum sp.	3	0	0	1	0	0
SM.B_Me_11818	Dicot. sp. indet.	190	0	0	0	0	0
SM.B_Me_11829	Laurophyllum sp.	3	0	0	1	0	0
SM.B_Me_11832	Dicot. sp. indet.	2 6	0	1	0	0	0
SM.B_Me_11840	Juglandaceae sp.	12	0	0	0	0	0
SM.B_Me_11841	Laurophyllum sp.	3	0	0	1	0	0
SM.B_Me_11848	Juglandaceae sp.	12	0	0	0	0	0
SM.B_Me_11851	Laurophyllum sp.	145	0	0	0	0	0
SM.B_Me_11855	Daphnogene sp.	12	0	0	0	0	0
SM.B_Me_11859	Ficus sp.	171 6	0	0	0	0	0
SM.B_Me_11860	Juglandaceae sp.	12	0	0	0	0	0
SM.B_Me_11861	Laurophyllum sp.	145	0	0	0	0	0
SM.B_Me_11869	Laurophyllum sp.	12	0	0	0	0	0
SM.B_Me_11871	Dicot. sp. indet.	195 6	0	0	0	0	0
SM.B_Me_11872	Juglandaceae sp.	13 76	0	0	0	0	0
SM.B_Me_11873	Juglandaceae sp.	12	0	0	0	0	0
SM.B_Me_11876	Juglandaceae sp.	12	0	0	0	0	0
SM.B_Me_11881	Dicot. sp. indet.	19	0	0	0	0	0
SM.B_Me_11884	Leguminosae sp. 5	147	0	0	0	0	0
SM.B_Me_11885	Juglandaceae sp.	187	0	0	0	0	0
SM.B_Me_11886	Laurophyllum sp.	3	0	0	1	0	0
SM.B_Me_11888	Dicot. sp. indet.	12	0	0	0	0	0
SM.B_Me_11898	Ulmaceae sp.	2	0	1	0	0	0
SM.B_Me_11899	Lauraceae sp.	12	0	0	0	0	0
SM.B_Me_11906	Juglandaceae sp.	14	0	0	0	0	0
SM.B_Me_11917	Juglandaceae sp.	14	0	0	0	0	0
SM.B_Me_11929	Lauraceae sp.	12	0	0	0	0	0
SM.B_Me_11938	Juglandaceae sp.	163	0	0	0	0	0
SM.B_Me_11940	Lauraceae sp.	12	0	0	0	0	0
SM.B_Me_11952	Dicot. sp. indet.	2	0	1	0	0	0
SM.B_Me_11968	Juglandaceae Formenkreis 1	188	0	0	0	0	0
SM.B_Me_11971	Leguminosae sp. 5	147	0	0	0	0	0

Wappler et al. SOM File S1

SM.B_Me_11986	Juglandaceae sp.	2			0	1	0	0	[illegible]
SM.B_Me_11994	Juglandaceae sp.	14			0	0	0	0	[illegible]
SM.B_Me_11997	Juglandaceae sp.	14			0	0	0	0	[illegible]
SM.B_Me_11998	Juglandaceae Formenkreis 1	188			0	0	0	0	[illegible]
SM.B_Me_12026	Juglandaceae sp.	163			0	0	0	0	[illegible]
SM.B_Me_12028	Juglandaceae sp.	12			0	0	0	0	[illegible]
SM.B_Me_12029	Juglandaceae sp.	163	12		0	0	0	0	[illegible]
SM.B_Me_12030	Juglandaceae sp.	3			0	0	1	0	[illegible]
SM.B_Me_12032	Juglandaceae sp.	163			0	0	0	0	[illegible]
SM.B_Me_12039	Juglandaceae sp.	14			0	0	0	0	[illegible]
SM.B_Me_12040	Dicot. sp. indet.	12			0	0	0	0	[illegible]
SM.B_Me_12043	Tremophyllum sp.	14			0	0	0	0	[illegible]
[illegible]	Laurophyllum sp.	12			0	0	0	0	[illegible]
SM.B_Me_12070	Juglandaceae sp.	163			0	0	0	0	[illegible]
SM.B_Me_12076	Lauraceae sp.	14			0	0	0	0	[illegible]
SM.B_Me_12079	Lauraceae sp.	12			0	0	0	0	[illegible]
SM.B_Me_12134	Juglandaceae sp.	163			0	0	0	0	[illegible]
SM.B_Me_12137	Juglandaceae sp.	14			0	0	0	0	[illegible]
SM.B_Me_12147	Ulmaceae sp.	143			0	0	0	0	[illegible]
SM.B_Me_12401	Lauraceae sp.	12			0	0	0	0	[illegible]
SM.B_Me_12406	Laurophyllum sp.	3			0	0	1	0	[illegible]
SM.B_Me_12417	Juglandaceae sp.	163			0	0	0	0	[illegible]
SM.B_Me_12425	Lauraceae sp.	12			0	0	0	0	[illegible]
SM.B_Me_12430	Dicot. sp. indet.	188			0	0	0	0	[illegible]
SM.B_Me_12441	Lauraceae sp.	12			0	0	0	0	[illegible]
SM.B_Me_12447	Dicot. sp. GM003	208			0	0	0	0	[illegible]
SM.B_Me_12449	Juglandaceae sp.	163			0	0	0	0	[illegible]
SM.B_Me_12450	Lauraceae sp.	12			0	0	0	0	[illegible]
SM.B_Me_12465	Laurophyllum lanigeroides	145			0	0	0	0	[illegible]
SM.B_Me_12473	Juglandaceae sp.	12			0	0	0	0	[illegible]
SM.B_Me_12474	Juglandaceae sp.	12			0	0	0	0	[illegible]
SM.B_Me_12507	Dicot. sp. indet.	12			0	0	0	0	[illegible]
SM.B_Me_12511	Dicot. sp. indet.	85			0	0	0	0	[illegible]
SM.B_Me_12529	Laurophyllum lanigeroides	57			0	0	0	0	[illegible]
SM.B_Me_1253	Laurophyllum lanigeroides	206			0	0	0	0	[illegible]
SM.B_Me_12530	Dicot. sp. indet.	45			0	0	0	0	[illegible]
SM.B_Me_12535	Leguminosae sp. 5	147			0	0	0	0	[illegible]
SM.B_Me_12541	Leguminosae sp. 5	3			0	0	1	0	[illegible]
SM.B_Me_12543	Leguminosae sp. 5	147			0	0	0	0	[illegible]
SM.B_Me_12546	Dicot. sp. indet.	12			0	0	0	0	[illegible]
SM.B_Me_12568	Dicot. sp. indet.	195			0	0	0	0	[illegible]
SM.B_Me_12569	Laurophyllum lanigeroides	145			0	0	0	0	[illegible]
SM.B_Me_12571	Laurophyllum lanigeroides	145	12	3	0	0	1	0	[illegible]
SM.B_Me_12583	Dicot. sp. indet.	14			0	0	0	0	[illegible]
SM.B_Me_12586	Juglandaceae sp.	163			0	0	0	0	[illegible]
SM.B_Me_12592	Daphnogene sp.	12			0	0	0	0	[illegible]
SM.B_Me_12593	Leguminosae sp. 5	147			0	0	0	0	[illegible]
SM.B_Me_12595	Dicot. sp. indet.	43			0	0	0	0	[illegible]
SM.B_Me_12596	Dicot. sp. indet.	211	105		0	0	0	0	[illegible]
SM.B_Me_12597	Juglandaceae sp.	163	12		0	0	0	0	[illegible]
SM.B_Me_12604	Juglandaceae sp.	12			0	0	0	0	[illegible]
SM.B_Me_12605	Lauraceae sp.	12			0	0	0	0	[illegible]
SM.B_Me_12606	Dicot. sp. indet.	208			0	0	0	0	[illegible]
SM.B_Me_12607	Juglandaceae sp.	163			0	0	0	0	[illegible]
SM.B_Me_12613	Leguminosae sp. 5	2			0	1	0	0	[illegible]
SM.B_Me_12618	Dicot. sp. indet.	57			0	0	0	0	[illegible]
SM.B_Me_12626	Laurophyllum sp.	146			0	0	0	0	[illegible]
SM.B_Me_12636	Dicot. sp. indet.	37			0	0	0	0	[illegible]
SM.B_Me_12643	? Illiciaceae sp.	208			0	0	0	0	[illegible]
SM.B_Me_12659	Dicot. sp. indet.	14			0	0	0	0	[illegible]
SM.B_Me_12663	Dicot. sp. indet.	32			0	0	0	0	[illegible]
SM.B_Me_12664	Dicot. sp. GM003	208	19		0	0	0	0	[illegible]
SM.B_Me_12668	Laurophyllum lanigeroides	117			0	0	0	0	[illegible]
SM.B_Me_1267	Leguminosae sp. 5	147			0	0	0	0	[illegible]
SM.B_Me_12675	Juglandaceae Formenkreis 1	188			0	0	0	0	[illegible]
SM.B_Me_12680	Dicot. sp. indet.	2			0	1	0	0	[illegible]
SM.B_Me_12683	Laurophyllum lanigeroides	12			0	0	0	0	[illegible]
SM.B_Me_12694	Dicot. sp. indet.	197	2		0	1	0	0	[illegible]
SM.B_Me_12697	Juglandaceae sp.	2			0	1	0	0	[illegible]
SM.B_Me_12698	Dicot. sp. indet.	76			0	0	0	0	[illegible]
SM.B_Me_12704	Dicot. sp. indet.	2			0	1	0	0	[illegible]
SM.B_Me_12706	Laurophyllum lanigeroides	2			0	1	0	0	[illegible]
SM.B_Me_12722	Laurophyllum sp.	2			0	1	0	0	[illegible]
SM.B_Me_1273	Dicot. sp. indet.	12			0	0	0	0	[illegible]
SM.B_Me_12730	Dicot. sp. indet.	163			0	0	0	0	[illegible]
SM.B_Me_12749	Laurophyllum sp.	76	12		0	0	0	0	[illegible]
SM.B_Me_12758	Dicot. sp. indet.	12			0	0	0	0	[illegible]
SM.B_Me_12759	Dicot. sp. GM005	195			0	0	0	0	[illegible]
SM.B_Me_12763	Leguminosae sp. 5	30	31		0	0	0	0	[illegible]
SM.B_Me_1278	Dicot. sp. indet.	163	12		0	0	0	0	[illegible]
SM.B_Me_12789	Lauraceae sp.	3			0	0	1	0	[illegible]
SM.B_Me_1279	? Illiciaceae sp.	3			0	0	1	0	[illegible]
SM.B_Me_12794	Lauraceae sp.	145	12		0	0	0	0	[illegible]
SM.B_Me_12803	Nymphaeaceae sp.	49			0	0	0	0	[illegible]
SM.B_Me_12805	Leguminosae sp. 5	1			1	0	0	0	[illegible]
SM.B_Me_12828	Dicot. sp. indet.	208	36		0	0	0	0	[illegible]
SM.B_Me_12829	Dicot. sp. indet.	12			0	0	0	0	[illegible]
SM.B_Me_12834	Dicot. sp. indet.	12			0	0	0	0	[illegible]
SM.B_Me_12835	Dicot. sp. indet.	3			0	0	1	0	[illegible]
SM.B_Me_12837	Juglandaceae sp.	3			0	0	1	0	[illegible]
SM.B_Me_12838	Juglandaceae sp.	14			0	0	0	0	[illegible]
SM.B_Me_12841	Lauraceae sp.	3	12		0	0	1	0	[illegible]
SM.B_Me_12844	Juglandaceae sp.	12			0	0	0	0	[illegible]
SM.B_Me_12868	Dicot. sp. GM005	22	14	12	0	0	0	0	[illegible]
SM.B_Me_12869	Juglandaceae sp.	190			0	0	0	0	[illegible]
SM.B_Me_12874	Dicot. sp. indet.	12			0	0	0	0	[illegible]
SM.B_Me_1290	Dicot. sp. indet.	145			0	0	0	0	[illegible]
SM.B_Me_1292	Laurophyllum sp.	190			0	0	0	0	[illegible]
SM.B_Me_12930	Tremophyllum sp.	12			0	0	0	0	[illegible]
SM.B_Me_12954	Tremophyllum sp.	12			0	0	0	0	[illegible]
SM.B_Me_1296	Juglandaceae sp.	143	12	142	0	0	0	0	[illegible]
SM.B_Me_1298	Dicot. sp. indet.	2			0	1	0	0	[illegible]
SM.B_Me_13017	Juglandaceae sp.	2			0	1	0	0	[illegible]
SM.B_Me_13018	Juglandaceae sp.	163			0	0	0	0	[illegible]
SM.B_Me_13022	Tremophyllum sp.	12	204		0	0	0	0	[illegible]
SM.B_Me_13024	Dicot. sp. indet.	187			0	0	0	0	[illegible]
SM.B_Me_13077	Lauraceae sp.	145			0	0	0	0	[illegible]
SM.B_Me_13143	Juglandaceae sp.	208			0	0	0	0	[illegible]
SM.B_Me_13145	Dicot. sp. indet.	12			0	0	0	0	[illegible]
SM.B_Me_13157	Dicot. sp. indet.	30			0	0	0	0	[illegible]
SM.B_Me_13159	Dicot. sp. indet.	12			0	0	0	0	[illegible]
SM.B_Me_13162	Laurophyllum lanigeroides	17			0	0	0	0	[illegible]
SM.B_Me_13164	Dicot. sp. indet.	12			0	0	0	0	[illegible]
SM.B_Me_13166	Juglandaceae sp.	163			0	0	0	0	[illegible]
SM.B_Me_13203	Dicot. sp. indet.	200			0	0	0	0	[illegible]
SM.B_Me_13208	Juglandaceae sp.	79			0	0	0	0	[illegible]
SM.B_Me_13209	Juglandaceae sp.	81			0	0	0	0	[illegible]
SM.B_Me_13212	? Illiciaceae sp.	14			0	0	0	0	[illegible]
SM.B_Me_13214	Juglandaceae sp.	12	17		0	0	0	0	[illegible]
SM.B_Me_13216	Dicot. sp. indet.	12			0	0	0	0	[illegible]
SM.B_Me_13221	Ulmaceae sp.	2	12		0	1	0	0	[illegible]
SM.B_Me_13223	Dicot. sp. indet.	12			0	0	0	0	[illegible]
SM.B_Me_13228	Juglandaceae Formenkreis 1	208	80	12	0	0	0	0	[illegible]
SM.B_Me_13233	Dicot. sp. indet.	12			0	0	0	0	[illegible]
SM.B_Me_13235	Juglandaceae sp.	163			0	0	0	0	[illegible]
SM.B_Me_13243	Dicot. sp. GM011	208			0	0	0	0	[illegible]
SM.B_Me_13244	Dicot. sp. indet.	12			0	0	0	0	[illegible]
SM.B_Me_13249	Juglandaceae sp.	163			0	0	0	0	[illegible]
SM.B_Me_13254	Juglandaceae sp.	14			0	0	0	0	[illegible]
SM.B_Me_13255	Laurophyllum sp.	12			0	0	0	0	[illegible]
SM.B_Me_13257	Dicot. sp. indet.	195			0	0	0	0	[illegible]
SM.B_Me_13259	Rhodomyrtophyllum sinuatum	12			0	0	0	0	[illegible]
SM.B_Me_1326	Dicot. sp. indet.	12			0	0	0	0	[illegible]
SM.B_Me_13266	Juglandaceae Formenkreis 1	80			0	0	0	0	[illegible]
SM.B_Me_13272	Dicot. sp. indet.	195			0	0	0	0	[illegible]
SM.B_Me_1328	Juglandaceae Formenkreis 1	208			0	0	0	0	[illegible]
SM.B_Me_13319	Dicot. sp. indet.	12	13		0	0	0	0	[illegible]
SM.B_Me_1333	Juglandaceae Formenkreis 1	12			0	0	0	0	[illegible]
SM.B_Me_13332	Juglandaceae Formenkreis 1	80			0	0	0	0	[illegible]
SM.B_Me_13348	Rosaceae sp.	12			0	0	0	0	[illegible]
SM.B_Me_13349	Dicot. sp. indet.	142			0	0	0	0	[illegible]
SM.B_Me_13350	Juglandaceae sp.	163			0	0	0	0	[illegible]
SM.B_Me_13357	Juglandaceae sp.	206			0	0	0	0	[illegible]
SM.B_Me_13360	Dicot. sp. indet.	81	14		0	0	0	0	[illegible]
SM.B_Me_13368	Juglandaceae sp.	163			0	0	0	0	[illegible]
SM.B_Me_13369	Juglandaceae sp.	2			0	1	0	0	[illegible]
SM.B_Me_1337	Juglandaceae Formenkreis 1	12			0	0	0	0	[illegible]
SM.B_Me_13373	Laurophyllum sp.	2			0	1	0	0	[illegible]
SM.B_Me_13383	Dicot. sp. GM009	17			0	0	0	0	[illegible]
SM.B_Me_13384	Dicot. sp. indet.	163			0	0	0	0	[illegible]
SM.B_Me_13392	Lauraceae sp.	12			0	0	0	0	[illegible]
SM.B_Me_13419	Leguminosae sp. 5	147			0	0	0	0	[illegible]
SM.B_Me_13422	Juglandaceae sp.	163			0	0	0	0	[illegible]
SM.B_Me_13433	Juglandaceae sp.	12			0	0	0	0	[illegible]
SM.B_Me_13434	Dicot. sp. indet.	195			0	0	0	0	[illegible]
SM.B_Me_13437	Vitaceae sp.	163			0	0	0	0	[illegible]
SM.B_Me_13447	Juglandaceae sp.	206	80		0	0	0	0	[illegible]
SM.B_Me_13448	Juglandaceae Formenkreis 1	190			0	0	0	0	[illegible]
SM.B_Me_13454	Rhodomyrtophyllum sinuatum	195			0	0	0	0	[illegible]
SM.B_Me_13460	Juglandaceae sp.	163	12		0	0	0	0	[illegible]
SM.B_Me_1347	Dicot. sp. indet.	30	13		0	0	0	0	[illegible]
SM.B_Me_13471	Juglandaceae Formenkreis 1	12			0	0	0	0	[illegible]
SM.B_Me_13477	Juglandaceae sp.	12			0	0	0	0	[illegible]
SM.B_Me_1348	Laurophyllum lanigeroides	145			0	0	0	0	[illegible]
SM.B_Me_13480	Araliaceae sp.	12			0	0	0	0	[illegible]
SM.B_Me_13488	Laurophyllum lanigeroides	3			0	0	1	0	[illegible]
SM.B_Me_13490	Ficus sp.	12			0	0	0	0	[illegible]
SM.B_Me_13497	Laurophyllum lanigeroides	2			0	1	0	0	[illegible]
SM.B_Me_1350	Laurophyllum lanigeroides	206			0	0	0	0	[illegible]
SM.B_Me_13500	Juglandaceae sp.	12			0	0	0	0	[illegible]
SM.B_Me_13507	Laurophyllum lanigeroides	13			0	0	0	0	[illegible]
SM.B_Me_1351	Juglandaceae Formenkreis 1	12			0	0	0	0	[illegible]
SM.B_Me_13517	Dicot. sp. indet.	3			0	0	1	0	[illegible]
SM.B_Me_13521	Dicot. sp. indet.	12			0	0	0	0	[illegible]
SM.B_Me_13525	Rhodomyrtophyllum sinuatum	50			0	0	0	0	[illegible]
SM.B_Me_13531	Juglandaceae sp.	12			0	0	0	0	[illegible]
SM.B_Me_13535	Juglandaceae sp.	3			0	0	1	0	[illegible]
SM.B_Me_1354	Juglandaceae Formenkreis 1	12			0	0	0	0	[illegible]
SM.B_Me_13542	Dicot. sp. indet.	36			0	0	0	0	[illegible]
SM.B_Me_13552	Juglandaceae sp.	62			0	0	0	0	[illegible]
SM.B_Me_13556	Juglandaceae sp.	12			0	0	0	0	[illegible]
SM.B_Me_13558	Dicot. sp. indet.	14			0	0	0	0	[illegible]
SM.B_Me_1356	Juglandaceae Formenkreis 1	2	36		0	1	0	0	[illegible]
SM.B_Me_13560	Dicot. sp. indet.	12			0	0	0	0	[illegible]
SM.B_Me_13575	Dicot. sp. indet.	15	22		0	0	0	0	[illegible]
SM.B_Me_13581	Laurophyllum lanigeroides	12	13		0	0	0	0	[illegible]
SM.B_Me_13589	cf. Apocynophyllum helveticum	163			0	0	0	0	[illegible]
SM.B_Me_13590	Rutaceae sp.	12			0	0	0	0	[illegible]
SM.B_Me_13592	Juglandaceae Formenkreis 1	14			0	0	0	0	[illegible]
SM.B_Me_136	Dicot. sp. indet.	2			0	1	0	0	[illegible]
SM.B_Me_13604	Dicot. sp. indet.	12			0	0	0	0	[illegible]
SM.B_Me_13607	Juglandaceae sp.	14			0	0	0	0	[illegible]
SM.B_Me_13608	Juglandaceae Formenkreis 1	188			0	0	0	0	[illegible]
SM.B_Me_1361	Leguminosae sp. 5	14			0	0	0	0	[illegible]
SM.B_Me_13610	Dicot. sp. indet.	2			0	1	0	0	[illegible]
SM.B_Me_1362	Juglandaceae sp.	12			0	0	0	0	[illegible]
SM.B_Me_13623	Juglandaceae sp.	12			0	0	0	0	[illegible]
SM.B_Me_13627	Juglandaceae sp.	81			0	0	0	0	[illegible]
SM.B_Me_13635	Laurophyllum lanigeroides	145			0	0	0	0	[illegible]
SM.B_Me_13638	Juglandaceae sp.	189			0	0	0	0	[illegible]
SM.B_Me_13642	Leguminosae sp. 5	147			0	0	0	0	[illegible]
SM.B_Me_13644	Dicot. sp. indet.	2			0	1	0	0	[illegible]
SM.B_Me_13651	Juglandaceae sp.	3	12		0	0	1	0	[illegible]
SM.B_Me_13662	Laurophyllum lanigeroides	168			0	0	0	0	[illegible]
SM.B_Me_13664	Juglandaceae sp.	12			0	0	0	0	[illegible]
SM.B_Me_13668	Juglandaceae sp.	14			0	0	0	0	[illegible]
SM.B_Me_13680	Juglandaceae sp.	189			0	0	0	0	[illegible]
SM.B_Me_13684	Juglandaceae sp.	12			0	0	0	0	[illegible]
SM.B_Me_13686	Juglandaceae sp.	76			0	0	0	0	[illegible]
SM.B_Me_13690	Juglandaceae sp.	220			0	0	0	0	[illegible]
SM.B_Me_13695	Juglandaceae sp.	210	12		0	0	0	0	[illegible]
SM.B_Me_13697	Juglandaceae sp.	149			0	0	0	0	[illegible]
SM.B_Me_13698	Dicot. sp. indet.	14			0	0	0	0	[illegible]
SM.B_Me_13699	Laurophyllum lanigeroides	146	145		0	0	0	0	[illegible]
SM.B_Me_13706	Juglandaceae sp.	32			0	0	0	0	[illegible]
SM.B_Me_13707	Dicot. sp. indet.	12	80		0	0	0	0	[illegible]
SM.B_Me_13709	Dicot. sp. indet.	79			0	0	0	0	[illegible]
SM.B_Me_13720	Tremophyllum sp.	197			0	0	0	0	[illegible]
SM.B_Me_1373	Juglandaceae Formenkreis 2	2			0	1	0	0	[illegible]
SM.B_Me_1375	Juglandaceae Formenkreis 1	12			0	0	0	0	[illegible]
SM.B_Me_1376	Dicot. sp. indet.	62			0	0	0	0	[illegible]
SM.B_Me_13797	Juglandaceae sp.	213			0	0	0	0	[illegible]
SM.B_Me_13844	Leguminosae sp. 5	197			0	0	0	0	[illegible]
SM.B_Me_13880	Dicot. sp. indet.	12			0	0	0	0	[illegible]
SM.B_Me_13896	Juglandaceae sp.	12			0	0	0	0	[illegible]
SM.B_Me_13905	Leguminosae sp. 5	176			0	0	0	0	[illegible]
SM.B_Me_13914	Laurophyllum sp.	206	13	3	0	0	1	0	[illegible]
SM.B_Me_13925	Juglandaceae sp.	76			0	0	0	0	[illegible]
SM.B_Me_13933	Juglandaceae sp.	12			0	0	0	0	[illegible]
SM.B_Me_13936	Laurophyllum lanigeroides	163			0	0	0	0	[illegible]
SM.B_Me_1394	Laurophyllum lanigeroides	117	12		0	0	0	0	[illegible]
SM.B_Me_13961	Juglandaceae Formenkreis 1	213	32		0	0	0	0	[illegible]
SM.B_Me_13964	Tremophyllum sp.	30			0	0	0	0	[illegible]
SM.B_Me_1398	Laurophyllum lanigeroides	30			0	0	0	0	[illegible]
SM.B_Me_13994	Juglandaceae sp.	195			0	0	0	0	[illegible]
SM.B_Me_1400	Dicot. sp. indet.	76			0	0	0	0	[illegible]
SM.B_Me_1402	Vitaceae sp.	195			0	0	0	0	[illegible]
SM.B_Me_14024	Juglandaceae sp.	195			0	0	0	0	[illegible]
SM.B_Me_14026	Juglandaceae sp.	197			0	0	0	0	[illegible]
SM.B_Me_14027	Ulmaceae sp.	12			0	0	0	0	[illegible]
SM.B_Me_14031	Juglandaceae sp.	14			0	0	0	0	[illegible]
SM.B_Me_14037	Juglandaceae sp.	3			0	0	1	0	[illegible]
SM.B_Me_1405	Dicot. sp. indet.	15			0	0	0	0	[illegible]
SM.B_Me_1407	Laurophyllum lanigeroides	12			0	0	0	0	[illegible]
SM.B_Me_1408	Dicot. sp. indet.	80			0	0	0	0	[illegible]
SM.B_Me_1412	Dicot. sp. indet.	13			0	0	0	0	[illegible]
SM.B_Me_1413	Dicot. sp. indet.	14			0	0	0	0	[illegible]
SM.B_Me_14149	Juglandaceae sp.	12	34		0	0	0	0	[illegible]
SM.B_Me_14152	Dicot. sp. GM003	208			0	0	0	0	[illegible]
SM.B_Me_14153	Dicot. sp. indet.	12			0	0	0	0	[illegible]
SM.B_Me_14154	Dicot. sp. GM003	208			0	0	0	0	[illegible]
SM.B_Me_14155	Dicot. sp. indet.	2			0	1	0	0	[illegible]
SM.B_Me_14163	Daphnogene sp.	2			0	1	0	0	[illegible]
SM.B_Me_14164	Lauraceae sp.	3			0	0	1	0	[illegible]
SM.B_Me_1417	Daphnogene crebrigranosa	14	12	13	0	0	0	0	[illegible]
SM.B_Me_14175	Juglandaceae sp.	12			0	0	0	0	[illegible]
SM.B_Me_14194	Dicot. sp. indet.	206	142		0	0	0	0	[illegible]
SM.B_Me_14195	Juglandaceae Formenkreis 1	12			0	0	0	0	[illegible]
SM.B_Me_1420	Laurophyllum lanigeroides	206	1		1	0	0	0	[illegible]
SM.B_Me_14216	Rhodomyrtophyllum sinuatum	145			0	0	0	0	[illegible]
SM.B_Me_14219	Juglandaceae Formenkreis 1	12			0	0	0	0	[illegible]
SM.B_Me_14220	Myricaceae sp.	36			0	0	0	0	[illegible]
SM.B_Me_14225	Juglandaceae sp.	213			0	0	0	0	[illegible]
SM.B_Me_14250	Juglandaceae sp.	12			0	0	0	0	[illegible]
SM.B_Me_14274	Juglandaceae sp.	12			0	0	0	0	[illegible]
SM.B_Me_1428	Ficus sp.	169			0	0	0	0	[illegible]
SM.B_Me_14285	Lauraceae sp.	145			0	0	0	0	[illegible]
SM.B_Me_14290	Juglandaceae sp.	12			0	0	0	0	[illegible]
SM.B_Me_14307	Juglandaceae sp.	163			0	0	0	0	[illegible]
SM.B_Me_14308	Juglandaceae sp.	14			0	0	0	0	[illegible]
SM.B_Me_1433	Dicot. sp. indet.	208			0	0	0	0	[illegible]
SM.B_Me_14367	Juglandaceae sp.	34			0	0	0	0	[illegible]
SM.B_Me_14369	Juglandaceae sp.	12			0	0	0	0	[illegible]
SM.B_Me_14380	Juglandaceae sp.	12			0	0	0	0	[illegible]
SM.B_Me_14394	Juglandaceae sp.	76			0	0	0	0	[illegible]
SM.B_Me_14398	Juglandaceae sp.	12			0	0	0	0	[illegible]
SM.B_Me_14401	Juglandaceae sp.	12			0	0	0	0	[illegible]
SM.B_Me_14403	Dicot. sp. GM003	2			0	1	0	0	[illegible]
SM.B_Me_14404	Laurophyllum lanigeroides	109			0	0	0	0	[illegible]
SM.B_Me_14407	Juglandaceae sp.	163			0	0	0	0	[illegible]
SM.B_Me_14425	Juglandaceae sp.	163			0	0	0	0	[illegible]
SM.B_Me_14479	Daphnogene sp.	14			0	0	0	0	[illegible]
SM.B_Me_14482	Leguminosae sp. 5	147			0	0	0	0	[illegible]
SM.B_Me_1451	Rhodomyrtophyllum sinuatum	12			0	0	0	0	[illegible]
SM.B_Me_14535	Laurophyllum sp.	13			0	0	0	0	[illegible]
SM.B_Me_1454	Laurophyllum sp.	200			0	0	0	0	[illegible]
SM.B_Me_14546	Juglandaceae sp.	163			0	0	0	0	[illegible]
SM.B_Me_14547	Lauraceae sp.	12			0	0	0	0	[illegible]
SM.B_Me_14548	Laurophyllum lanigeroides	145			0	0	0	0	[illegible]
SM.B_Me_14550	Laurophyllum lanigeroides	17			0	0	0	0	[illegible]
SM.B_Me_14552	Laurophyllum lanigeroides	3			0	0	1	0	[illegible]
SM.B_Me_14555	Laurophyllum lanigeroides	3	36	12	0	0	1	0	[illegible]
SM.B_Me_14560	Dicot. sp. indet.	187			0	0	0	0	[illegible]
SM.B_Me_14568	Juglandaceae sp.	163			0	0	0	0	[illegible]
SM.B_Me_14571	Laurophyllum lanigeroides	3	36	12	0	0	1	0	[illegible]
SM.B_Me_14577	Juglandaceae Formenkreis 1	190			0	0	0	0	[illegible]
SM.B_Me_14578	Dicot. sp. indet.	195			0	0	0	0	[illegible]
SM.B_Me_14592	Dicot. sp. GM003	208			0	0	0	0	[illegible]
SM.B_Me_14594	Dicot. sp. indet.	12			0	0	0	0	[illegible]
SM.B_Me_14604	Juglandaceae Formenkreis 1	194	12		0	0	0	0	[illegible]
SM.B_Me_14612	Dicot. sp. indet.	214			0	0	0	0	[illegible]
SM.B_Me_14617	Ficus sp.	200			0	0	0	0	[illegible]
SM.B_Me_14619	Laurophyllum sp.	12			0	0	0	0	[illegible]
SM.B_Me_1462	Juglandaceae sp.	6			0	0	0	0	[illegible]
SM.B_Me_14622	Lauraceae sp.	163	188	36	0	0	0	0	[illegible]
SM.B_Me_14628	Vitaceae sp.	210			0	0	0	0	[illegible]
SM.B_Me_14637	Juglandaceae sp.	14			0	0	0	0	[illegible]
SM.B_Me_14644	Lauraceae sp.	12			0	0	0	0	[illegible]
SM.B_Me_14646	Lauraceae sp.	2			0	1	0	0	[illegible]
SM.B_Me_1465	Daphnogene crebrigranosa	12			0	0	0	0	[illegible]
SM.B_Me_14656	Lauraceae sp.	12			0	0	0	0	[illegible]
SM.B_Me_14681	Juglandaceae sp.	12			0	0	0	0	[illegible]
SM.B_Me_14682	Juglandaceae sp.	213			0	0	0	0	[illegible]
SM.B_Me_14692	Daphnogene sp.	2			0	1	0	0	[illegible]
SM.B_Me_14693	Toddalia ovata	208			0	0	0	0	[illegible]
SM.B_Me_14696	Juglandaceae sp.	12			0	0	0	0	[illegible]
SM.B_Me_14697	Laurophyllum lanigeroides	12			0	0	0	0	[illegible]
SM.B_Me_14709	Laurophyllum sp.	12			0	0	0	0	[illegible]
SM.B_Me_14711	Juglandaceae sp.	2			0	1	0	0	[illegible]
SM.B_Me_14715	Juglandaceae sp.	145			0	0	0	0	[illegible]
SM.B_Me_1472	Laurophyllum hirsutum	81	2		0	1	0	0	[illegible]
SM.B_Me_1486	Dicot. sp. indet.	2			0	1	0	0	[illegible]
SM.B_Me_14897	Tremophyllum sp.	143			0	0	0	0	[illegible]
SM.B_Me_14922	Juglandaceae sp.	12			0	0	0	0	[illegible]
SM.B_Me_1494	Nyssa sp.	195			0	0	0	0	[illegible]
SM.B_Me_14991	Ulmaceae sp.	143			0	0	0	0	[illegible]
SM.B_Me_14992	Ulmaceae sp.	143			0	0	0	0	[illegible]
SM.B_Me_15041	Ulmaceae sp.	12			0	0	0	0	[illegible]
SM.B_Me_15042	Dicot. sp. indet.	12			0	0	0	0	[illegible]
SM.B_Me_1509	Laurophyllum hirsutum	206			0	0	0	0	[illegible]
SM.B_Me_1511	Juglandaceae Formenkreis 1	12			0	0	0	0	[illegible]
SM.B_Me_15123	Laurophyllum sp.	12			0	0	0	0	[illegible]
SM.B_Me_15128	Juglandaceae sp.	12			0	0	0	0	[illegible]
SM.B_Me_15134	Juglandaceae sp.	12			0	0	0	0	[illegible]
SM.B_Me_15138	Dicot. sp. GM002	2	14		0	1	0	0	[illegible]
SM.B_Me_15139	Juglandaceae sp.	12			0	0	0	0	[illegible]
SM.B_Me_15149	Juglandaceae Formenkreis 1	12			0	0	0	0	[illegible]
SM.B_Me_15152	Dicot. sp. indet.	85			0	0	0	0	[illegible]
SM.B_Me_15159	Dicot. sp. indet.	146			0	0	0	0	[illegible]
SM.B_Me_1516	Leguminosae sp. 5	147			0	0	0	0	[illegible]
SM.B_Me_15160	Dicot. sp. GM003	7	62		0	0	0	0	[illegible]
SM.B_Me_15161	Juglandaceae sp.	19			0	0	0	0	[illegible]
SM.B_Me_15162	Dicot. sp. indet.	185			0	0	0	0	[illegible]
SM.B_Me_15170	Juglandaceae sp.	12			0	0	0	0	[illegible]
SM.B_Me_15172	Juglandaceae sp.	12			0	0	0	0	[illegible]
SM.B_Me_15175	Juglandaceae sp.	12			0	0	0	0	[illegible]
SM.B_Me_15182	Juglandaceae Formenkreis 1	12			0	0	0	0	[illegible]
SM.B_Me_15188	Dicot. sp. indet.	200			0	0	0	0	[illegible]
SM.B_Me_15194	Leguminosae sp. 5	147			0	0	0	0	[illegible]
SM.B_Me_15201	Dicot. sp. indet.	200			0	0	0	0	[illegible]
SM.B_Me_15204	Dicot. sp. indet.	12			0	0	0	0	[illegible]
SM.B_Me_1521	Dicot. sp. GM004	80	1		1	0	0	0	[illegible]
SM.B_Me_15214	Dicot. sp. indet.	13			0	0	0	0	[illegible]
SM.B_Me_15215	Laurophyllum sp.	12			0	0	0	0	[illegible]
SM.B_Me_15217	Dicot. sp. indet.	145			0	0	0	0	[illegible]
SM.B_Me_15218	Dicot. sp. GM004	80			0	0	0	0	[illegible]
SM.B_Me_15227	Dicot. sp. indet.	200			0	0	0	0	[illegible]
SM.B_Me_15228	Laurophyllum lanigeroides	145			0	0	0	0	[illegible]
SM.B_Me_15229	Juglandaceae sp.	12			0	0	0	0	[illegible]
SM.B_Me_15233	Lauraceae sp.	62			0	0	0	0	[illegible]
SM.B_Me_15234	Juglandaceae sp.	12			0	0	0	0	[illegible]
SM.B_Me_15235	Lauraceae sp.	14			0	0	0	0	[illegible]
SM.B_Me_15237	Juglandaceae sp.	12			0	0	0	0	[illegible]
SM.B_Me_15239	Juglandaceae sp.	12			0	0	0	0	[illegible]
SM.B_Me_15246	Juglandaceae sp.	12			0	0	0	0	[illegible]
SM.B_Me_15247	Lauraceae sp.	214	12		0	0	0	0	[illegible]
SM.B_Me_15248	Ulmaceae sp.	12			0	0	0	0	[illegible]
SM.B_Me_15263	Juglandaceae sp.	12			0	0	0	0	[illegible]
SM.B_Me_1527	Leguminosae sp. 5	147			0	0	0	0	[illegible]

SM.B_Me_15270	Juglandaceae sp.	190				0	0	0	0	0	[illegible]
SM.B_Me_15273	Juglandaceae sp.	12				0	0	0	0	0	[illegible]
SM.B_Me_15287	Leguminosae sp. 5	12				0	0	0	0	0	[illegible]
SM.B_Me_15288	Ulmaceae sp.	12	25			0	0	0	0	0	[illegible]
SM.B_Me_15290	Juglandaceae sp.	12				0	0	0	0	0	[illegible]
SM.B_Me_15293	Dicot. sp. indet.	15				0	0	0	0	0	[illegible]
SM.B_Me_15295	Juglandaceae sp.	12				0	0	0	0	0	[illegible]
SM.B_Me_15297	Lauraceae sp.	12				0	0	0	0	0	[illegible]
SM.B_Me_15304	Laurophyllum sp.	17	12			0	0	0	0	0	[illegible]
SM.B_Me_1531	Ficus sp.	168	188			0	0	0	0	0	[illegible]
SM.B_Me_15310	Lauraceae sp.	14				0	0	0	0	0	[illegible]
SM.B_Me_15315	Lauraceae sp.	17				0	0	0	0	0	[illegible]
[illegible]	Laurophyllum lanigeroides	3				0	0	1	0	0	[illegible]
SM.B_Me_1534	Dicot. sp. indet.	168				0	0	0	0	0	[illegible]
SM.B_Me_1537	Laurophyllum lanigeroides	189				0	0	0	0	0	[illegible]
SM.B_Me_15373	Lauraceae sp.	12				0	0	0	0	0	[illegible]
SM.B_Me_15375	Juglandaceae sp.	163				0	0	0	0	0	[illegible]
SM.B_Me_15378	Dicot. sp. indet.	12				0	0	0	0	0	[illegible]
SM.B_Me_15380	Lauraceae sp.	12				0	0	0	0	0	[illegible]
SM.B_Me_15381	Juglandaceae sp.	163	36	12		0	0	0	0	0	[illegible]
SM.B_Me_15385	Juglandaceae sp.	12				0	0	0	0	0	[illegible]
SM.B_Me_15388	Lauraceae sp.	169				0	0	0	0	0	[illegible]
SM.B_Me_15391	Lauraceae sp.	146				0	0	0	0	0	[illegible]
SM.B_Me_15397	Juglandaceae sp.	12				0	0	0	0	0	[illegible]
SM.B_Me_15400	Juglandaceae sp.	213				0	0	0	0	0	[illegible]
SM.B_Me_15406	Laurophyllum lanigeroides	145				0	0	0	0	0	[illegible]
SM.B_Me_15428	Laurophyllum lanigeroides	3				0	0	1	0	0	[illegible]
SM.B_Me_1545	Dicot. sp. indet.	53	19	171	105	0	0	0	0	0	[illegible]
SM.B_Me_1547	Dicot. sp. indet.	12				0	0	0	0	0	[illegible]
SM.B_Me_15482	Lauraceae sp.	12				0	0	0	0	0	[illegible]
SM.B_Me_15484	Daphnogene sp.	208				0	0	0	0	0	[illegible]
SM.B_Me_15488	Juglandaceae sp.	12				0	0	0	0	0	[illegible]
SM.B_Me_1549	Juglandaceae Formenkreis 1	2				0	1	0	0	0	[illegible]
SM.B_Me_155	Daphnogene sp.	206				0	0	0	0	0	[illegible]
SM.B_Me_15510	Dicot. sp. indet.	2				0	1	0	0	0	[illegible]
SM.B_Me_1563	Dicot. sp. indet.	195				0	0	0	0	0	[illegible]
SM.B_Me_15675	Juglandaceae sp.	163				0	0	0	0	0	[illegible]
SM.B_Me_15684	Dicot. sp. indet.	12				0	0	0	0	0	[illegible]
SM.B_Me_15687	Lauraceae sp.	214				0	0	0	0	0	[illegible]
SM.B_Me_15696	Juglandaceae sp.	163				0	0	0	0	0	[illegible]
SM.B_Me_15701	Juglandaceae sp.	12				0	0	0	0	0	[illegible]
SM.B_Me_15704	Rhodomyrtophyllum sinuatum	145				0	0	0	0	0	[illegible]
SM.B_Me_15705	Laurophyllum lanigeroides	3				0	0	1	0	0	[illegible]
SM.B_Me_15725	Laurophyllum lanigeroides	145	12			0	0	0	0	0	[illegible]
SM.B_Me_15732	Dicot. sp. indet.	208				0	0	0	0	0	[illegible]
SM.B_Me_15742	Leguminosae sp. 5	12				0	0	0	0	0	[illegible]
SM.B_Me_15743	Laurophyllum lanigeroides	36				0	0	0	0	0	[illegible]
SM.B_Me_15752	Laurophyllum lanigeroides	145				0	0	0	0	0	[illegible]
SM.B_Me_15759	Laurophyllum lanigeroides	117	16			0	0	0	0	0	[illegible]
SM.B_Me_1576	Dicot. sp. GM001	195				0	0	0	0	0	[illegible]
SM.B_Me_15761	Laurophyllum lanigeroides	3				0	0	1	0	0	[illegible]
SM.B_Me_15764	Dicot. sp. indet.	12				0	0	0	0	0	[illegible]
SM.B_Me_15765	Laurophyllum lanigeroides	3				0	0	1	0	0	[illegible]
SM.B_Me_15766	Laurophyllum lanigeroides	3	12			0	0	1	0	0	[illegible]
SM.B_Me_15768	Daphnogene sp.	220				0	0	0	0	0	[illegible]
SM.B_Me_15781	Lauraceae sp.	201				0	0	0	0	0	[illegible]
SM.B_Me_15783	Juglandaceae sp.	14				0	0	0	0	0	[illegible]
SM.B_Me_15788	Dicot. sp. indet.	200				0	0	0	0	0	[illegible]
SM.B_Me_15789	Juglandaceae Formenkreis 1	12				0	0	0	0	0	[illegible]
SM.B_Me_15799	Rhodomyrtophyllum sinuatum	62				0	0	0	0	0	[illegible]
SM.B_Me_15802	Dicot. sp. indet.	50	76			0	0	0	0	0	[illegible]
SM.B_Me_15803	Juglandaceae Formenkreis 1	12				0	0	0	0	0	[illegible]
SM.B_Me_15818	Juglandaceae sp.	4				0	0	0	1	0	[illegible]
SM.B_Me_15820	Nyssa sp.	2				0	1	0	0	0	[illegible]
SM.B_Me_1586	Dicot. sp. indet.	195				0	0	0	0	0	[illegible]
SM.B_Me_1592	Laurophyllum lanigeroides	145	13			0	0	0	0	0	[illegible]
SM.B_Me_15936	Lauraceae sp.	145				0	0	0	0	0	[illegible]
SM.B_Me_15944	Dicot. sp. indet.	19				0	0	0	0	0	[illegible]
SM.B_Me_1595	Dicot. sp. indet.	12				0	0	0	0	0	[illegible]
SM.B_Me_15956	Lauraceae sp.	146	3			0	0	1	0	0	[illegible]
SM.B_Me_15963	Dicot. sp. indet.	22				0	0	0	0	0	[illegible]
SM.B_Me_15964	Lauraceae sp.	13				0	0	0	0	0	[illegible]
SM.B_Me_15968	Dicot. sp. indet.	6				0	0	0	0	0	[illegible]
SM.B_Me_15980	Nymphaeaceae sp.	49				0	0	0	0	0	[illegible]
SM.B_Me_15981	Nymphaeaceae sp.	49				0	0	0	0	0	[illegible]
SM.B_Me_15994	Leguminosae sp. 5	147				0	0	0	0	0	[illegible]
SM.B_Me_15997	Juglandaceae sp.	163				0	0	0	0	0	[illegible]
SM.B_Me_16000	Juglandaceae sp.	163				0	0	0	0	0	[illegible]
SM.B_Me_16012	Dicot. sp. GM012	45				0	0	0	0	0	[illegible]
SM.B_Me_16015	Rhodomyrtophyllum sinuatum	12				0	0	0	0	0	[illegible]
SM.B_Me_16016	Juglandaceae sp.	2				0	1	0	0	0	[illegible]
SM.B_Me_16017	Juglandaceae sp.	12				0	0	0	0	0	[illegible]
SM.B_Me_16025	Juglandaceae Formenkreis 1	190				0	0	0	0	0	[illegible]
SM.B_Me_16029	Laurophyllum lanigeroides	146	2	14		0	1	0	0	0	[illegible]
SM.B_Me_16032	Lauraceae sp.	3				0	0	1	0	0	[illegible]
SM.B_Me_16040	Juglandaceae Formenkreis 1	213	4			0	0	0	1	0	[illegible]
SM.B_Me_1609	Leguminosae sp. 5	147				0	0	0	0	0	[illegible]
SM.B_Me_161	Dicot. sp. indet.	216				0	0	0	0	0	[illegible]
SM.B_Me_16122	Dicot. sp. indet.	12				0	0	0	0	0	[illegible]
SM.B_Me_16154	Lauraceae sp.	36				0	0	0	0	0	[illegible]
SM.B_Me_16157	Laurophyllum lanigeroides	145				0	0	0	0	0	[illegible]
SM.B_Me_16158	Dicot. sp. indet.	2	76			0	1	0	0	0	[illegible]
SM.B_Me_16160	Lauraceae sp.	85	3	12		0	0	1	0	0	[illegible]
SM.B_Me_16166	Juglandaceae Formenkreis 1	213				0	0	0	0	0	[illegible]
SM.B_Me_16171	Juglandaceae sp.	2				0	1	0	0	0	[illegible]
SM.B_Me_16173	Leguminosae sp. 5	147				0	0	0	0	0	[illegible]
SM.B_Me_16180	Daphnogene sp.	14				0	0	0	0	0	[illegible]
SM.B_Me_16187	Juglandaceae sp.	12				0	0	0	0	0	[illegible]
SM.B_Me_1622	? Illiciaceae sp.	17				0	0	0	0	0	[illegible]
SM.B_Me_1627	Juglandaceae Formenkreis 1	189				0	0	0	0	0	[illegible]
SM.B_Me_16272	Dicot. sp. indet.	195				0	0	0	0	0	[illegible]
SM.B_Me_16276	Dicot. sp. indet.	210				0	0	0	0	0	[illegible]
SM.B_Me_1628	Juglandaceae Formenkreis 1	190				0	0	0	0	0	[illegible]
SM.B_Me_16281	Juglandaceae Formenkreis 1	12				0	0	0	0	0	[illegible]
SM.B_Me_16283	Dicot. sp. indet.	12				0	0	0	0	0	[illegible]
SM.B_Me_16287	Dicot. sp. indet.	195				0	0	0	0	0	[illegible]
SM.B_Me_16292	Leguminosae sp. 5	36				0	0	0	0	0	[illegible]
SM.B_Me_163	Dicot. sp. indet.	32	2			0	1	0	0	0	[illegible]
SM.B_Me_16307	Juglandaceae sp.	12				0	0	0	0	0	[illegible]
SM.B_Me_16310	Dicot. sp. indet.	26				0	0	0	0	0	[illegible]
SM.B_Me_16311	Juglandaceae sp.	12				0	0	0	0	0	[illegible]
SM.B_Me_16312	Rutaceae sp.	189				0	0	0	0	0	[illegible]
SM.B_Me_1632	Juglandaceae Formenkreis 1	211				0	0	0	0	0	[illegible]
SM.B_Me_16322	Juglandaceae sp.	163				0	0	0	0	0	[illegible]
SM.B_Me_16324	Juglandaceae sp.	12				0	0	0	0	0	[illegible]
SM.B_Me_16326	Juglandaceae sp.	195				0	0	0	0	0	[illegible]
SM.B_Me_16332	Rutaceae sp.	2				0	1	0	0	0	[illegible]
SM.B_Me_16333	Dicot. sp. indet.	195				0	0	0	0	0	[illegible]
SM.B_Me_16334	Laurophyllum sp.	189	12	2		0	1	0	0	0	[illegible]
SM.B_Me_16336	Dicot. sp. indet.	195				0	0	0	0	0	[illegible]
SM.B_Me_16339	Lauraceae sp.	14				0	0	0	0	0	[illegible]
SM.B_Me_16344	Dicot. sp. indet.	207				0	0	0	0	0	[illegible]
SM.B_Me_1635	Dicot. sp. indet.	119				0	0	0	0	0	[illegible]
SM.B_Me_1639	Laurophyllum lanigeroides	2				0	1	0	0	0	[illegible]
SM.B_Me_1640	Ternstroemites engelhardtii	207				0	0	0	0	0	[illegible]
SM.B_Me_1644	Juglandaceae Formenkreis 1	76				0	0	0	0	0	[illegible]
SM.B_Me_16472	Leguminosae sp. 5	208	14	12		0	0	0	0	0	[illegible]
SM.B_Me_16515	Juglandaceae sp.	213				0	0	0	0	0	[illegible]
SM.B_Me_16516	Dicot. sp. indet.	207	206	14		0	0	0	0	0	[illegible]
SM.B_Me_16533	Dicot. sp. GM003	208				0	0	0	0	0	[illegible]
SM.B_Me_16534	Laurophyllum sp.	168				0	0	0	0	0	[illegible]
SM.B_Me_16544	Juglandaceae Formenkreis 1	190				0	0	0	0	0	[illegible]
SM.B_Me_16556	Daphnogene sp.	2				0	1	0	0	0	[illegible]
SM.B_Me_16557	Juglandaceae Formenkreis 1	190				0	0	0	0	0	[illegible]
SM.B_Me_1656	Toddalia ovata	207				0	0	0	0	0	[illegible]
SM.B_Me_16587	Juglandaceae sp.	26				0	0	0	0	0	[illegible]
SM.B_Me_16599	Juglandaceae sp.	163				0	0	0	0	0	[illegible]
SM.B_Me_1660	Juglandaceae Formenkreis 2	142				0	0	0	0	0	[illegible]
SM.B_Me_1661	Dicot. sp. indet.	106				0	0	0	0	0	[illegible]
SM.B_Me_16611	Dicot. sp. indet.	2	12			0	1	0	0	0	[illegible]
SM.B_Me_16617	Juglandaceae sp.	163				0	0	0	0	0	[illegible]
SM.B_Me_16619	Laurophyllum lanigeroides	145	76	12	17	0	0	0	0	0	[illegible]
SM.B_Me_16622	Juglandaceae Formenkreis 1	12				0	0	0	0	0	[illegible]
SM.B_Me_1663	Juglandaceae Formenkreis 1	142				0	0	0	0	0	[illegible]
SM.B_Me_16630	Laurophyllum lanigeroides	12				0	0	0	0	0	[illegible]
SM.B_Me_16634	Laurophyllum lanigeroides	187				0	0	0	0	0	[illegible]
SM.B_Me_16662	Juglandaceae Formenkreis 1	12				0	0	0	0	0	[illegible]
SM.B_Me_16666	Lauraceae sp.	168				0	0	0	0	0	[illegible]
SM.B_Me_16669	Juglandaceae Formenkreis 1	215				0	0	0	0	0	[illegible]
SM.B_Me_16694	Rhodomyrtophyllum sinuatum	14				0	0	0	0	0	[illegible]
SM.B_Me_1671	Juglandaceae Formenkreis 1	12				0	0	0	0	0	[illegible]
SM.B_Me_16713	Dicot. sp. GM002	188				0	0	0	0	0	[illegible]
SM.B_Me_16716	Daphnogene sp.	2				0	1	0	0	0	[illegible]
SM.B_Me_16735	Dicot. sp. indet.	188	145			0	0	0	0	0	[illegible]
SM.B_Me_1674	Juglandaceae Formenkreis 1	142				0	0	0	0	0	[illegible]
SM.B_Me_16741	Dicot. sp. indet.	208	145			0	0	0	0	0	[illegible]
SM.B_Me_16775	Lauraceae sp.	3				0	0	1	0	0	[illegible]
SM.B_Me_16777	Juglandaceae sp.	12				0	0	0	0	0	[illegible]
SM.B_Me_1679	Leguminosae sp. 1	147	12			0	0	0	0	0	[illegible]
SM.B_Me_16800	Dicot. sp. indet.	3				0	0	1	0	0	[illegible]
SM.B_Me_16802	Nymphaeaceae sp.	49				0	0	0	0	0	[illegible]
SM.B_Me_16804	Juglandaceae sp.	216				0	0	0	0	0	[illegible]
SM.B_Me_16806	Ficus sp.	45				0	0	0	0	0	[illegible]
SM.B_Me_16812	Dicot. sp. indet.	12				0	0	0	0	0	[illegible]
SM.B_Me_16865	Lauraceae sp.	12				0	0	0	0	0	[illegible]
SM.B_Me_16868	Juglandaceae sp.	12				0	0	0	0	0	[illegible]
SM.B_Me_16869	Lauraceae sp.	17	206			0	0	0	0	0	[illegible]
SM.B_Me_1687	Dicot. sp. GM003	22				0	0	0	0	0	[illegible]
SM.B_Me_16908	Juglandaceae sp.	163				0	0	0	0	0	[illegible]
SM.B_Me_16909	Lauraceae sp.	2				0	1	0	0	0	[illegible]
SM.B_Me_1691	Dicot. sp. indet.	196				0	0	0	0	0	[illegible]
SM.B_Me_16911	Juglandaceae sp.	12				0	0	0	0	0	[illegible]
SM.B_Me_16913	Juglandaceae sp.	12				0	0	0	0	0	[illegible]
SM.B_Me_16915	Leguminosae sp. 5	206				0	0	0	0	0	[illegible]
SM.B_Me_1693	Dicot. sp. indet.	196				0	0	0	0	0	[illegible]
SM.B_Me_16957	Juglandaceae sp.	163	12			0	0	0	0	0	[illegible]
SM.B_Me_1696	Dicot. sp. indet.	176	3			0	0	1	0	0	[illegible]
SM.B_Me_16964	Juglandaceae sp.	190				0	0	0	0	0	[illegible]
SM.B_Me_16984	Juglandaceae Formenkreis 1	19				0	0	0	0	0	[illegible]
SM.B_Me_16985	Juglandaceae Formenkreis 1	76				0	0	0	0	0	[illegible]
SM.B_Me_16987	Lauraceae sp.	3				0	0	1	0	0	[illegible]
SM.B_Me_16991	Juglandaceae Formenkreis 1	14				0	0	0	0	0	[illegible]
SM.B_Me_16992	Juglandaceae sp.	12				0	0	0	0	0	[illegible]
SM.B_Me_17022	Tremophyllum sp.	143				0	0	0	0	0	[illegible]
SM.B_Me_17025	Laurophyllum sp.	12				0	0	0	0	0	[illegible]
SM.B_Me_17026	Tremophyllum sp.	36				0	0	0	0	0	[illegible]
SM.B_Me_17029	Juglandaceae sp.	190				0	0	0	0	0	[illegible]
SM.B_Me_1705	Dicot. sp. indet.	34	12			0	0	0	0	0	[illegible]
SM.B_Me_1706	Dicot. sp. GM004	80				0	0	0	0	0	[illegible]
SM.B_Me_17064	Juglandaceae Formenkreis 1	12				0	0	0	0	0	[illegible]
SM.B_Me_17068	Dicot. sp. indet.	2				0	1	0	0	0	[illegible]
SM.B_Me_17081	Juglandaceae Formenkreis 1	213	12			0	0	0	0	0	[illegible]
SM.B_Me_17095	Dicot. sp. GM002	80				0	0	0	0	0	[illegible]
SM.B_Me_17096	Dicot. sp. indet.	14				0	0	0	0	0	[illegible]
SM.B_Me_17099	Myricaceae sp.	201				0	0	0	0	0	[illegible]
SM.B_Me_171	Dicot. sp. indet.	2				0	1	0	0	0	[illegible]
SM.B_Me_1710	Laurophyllum lanigeroides	142				0	0	0	0	0	[illegible]
SM.B_Me_17108	Juglandaceae sp.	12				0	0	0	0	0	[illegible]
SM.B_Me_17111	Toddalia ovata	163				0	0	0	0	0	[illegible]
SM.B_Me_1712	Dicot. sp. GM009	17				0	0	0	0	0	[illegible]
SM.B_Me_17146	Daphnogene sp.	2				0	1	0	0	0	[illegible]
SM.B_Me_17147	Lauraceae sp.	12				0	0	0	0	0	[illegible]
SM.B_Me_17149	Lauraceae sp.	5				0	0	0	0	1	[illegible]
SM.B_Me_17161	Laurophyllum lanigeroides	3				0	0	1	0	0	[illegible]
SM.B_Me_17162	Lauraceae sp.	5				0	0	0	0	1	[illegible]
SM.B_Me_17165	Daphnogene sp.	12				0	0	0	0	0	[illegible]
SM.B_Me_17167	Juglandaceae sp.	163				0	0	0	0	0	[illegible]
SM.B_Me_17168	Lauraceae sp.	12				0	0	0	0	0	[illegible]
SM.B_Me_17170	Juglandaceae sp.	12				0	0	0	0	0	[illegible]
SM.B_Me_17173	Lauraceae sp.	12				0	0	0	0	0	[illegible]
SM.B_Me_17174	Dicot. sp. GM003	85				0	0	0	0	0	[illegible]
SM.B_Me_17175	Lauraceae sp.	12	3			0	0	1	0	0	[illegible]
SM.B_Me_17199	Juglandaceae Formenkreis 1	14				0	0	0	0	0	[illegible]
SM.B_Me_172	Dicot. sp. indet.	32	12			0	0	0	0	0	[illegible]
SM.B_Me_17200	Dicot. sp. GM003	208				0	0	0	0	0	[illegible]
SM.B_Me_17201	Juglandaceae sp.	157				0	0	0	0	0	[illegible]
SM.B_Me_17206	Vitaceae sp.	209				0	0	0	0	0	[illegible]
SM.B_Me_17208	Laurophyllum lanigeroides	3	12			0	0	1	0	0	[illegible]
SM.B_Me_17211	Juglandaceae sp.	14				0	0	0	0	0	[illegible]
SM.B_Me_17212	Laurophyllum lanigeroides	14				0	0	0	0	0	[illegible]
SM.B_Me_17213	Juglandaceae sp.	12				0	0	0	0	0	[illegible]
SM.B_Me_17219	Juglandaceae sp.	85				0	0	0	0	0	[illegible]
SM.B_Me_17221	Lauraceae sp.	2				0	1	0	0	0	[illegible]
SM.B_Me_17228	Juglandaceae Formenkreis 1	190				0	0	0	0	0	[illegible]
SM.B_Me_17235	Lauraceae sp.	146				0	0	0	0	0	[illegible]
SM.B_Me_17236	Juglandaceae Formenkreis 1	190				0	0	0	0	0	[illegible]
SM.B_Me_17237	Juglandaceae Formenkreis 1	14				0	0	0	0	0	[illegible]
SM.B_Me_17239	Magnoliaceae sp.	146				0	0	0	0	0	[illegible]
SM.B_Me_17242	Lauraceae sp.	3				0	0	1	0	0	[illegible]
SM.B_Me_17243	Dicot. sp. indet.	195				0	0	0	0	0	[illegible]
SM.B_Me_17250	Juglandaceae sp.	12				0	0	0	0	0	[illegible]
SM.B_Me_17253	Leguminosae sp. 5	147				0	0	0	0	0	[illegible]
SM.B_Me_17256	Dicot. sp. indet.	3				0	0	1	0	0	[illegible]
SM.B_Me_17263	Juglandaceae sp.	12				0	0	0	0	0	[illegible]
SM.B_Me_17264	Juglandaceae sp.	163				0	0	0	0	0	[illegible]
SM.B_Me_17269	Lauraceae sp.	12				0	0	0	0	0	[illegible]
SM.B_Me_17276	Juglandaceae sp.	12				0	0	0	0	0	[illegible]
SM.B_Me_1731	Juglandaceae sp.	171				0	0	0	0	0	[illegible]
SM.B_Me_1734	? Ulmoideae Formenkreis 1	12				0	0	0	0	0	[illegible]
SM.B_Me_17349	Leguminosae sp. 5	12				0	0	0	0	0	[illegible]
SM.B_Me_17351	Juglandaceae sp.	12				0	0	0	0	0	[illegible]
SM.B_Me_17353	Juglandaceae Formenkreis 1	12				0	0	0	0	0	[illegible]
SM.B_Me_17362	Laurophyllum sp.	3				0	0	1	0	0	[illegible]
SM.B_Me_17385	Lauraceae sp.	2				0	1	0	0	0	[illegible]
SM.B_Me_17387	Lauraceae sp.	30				0	0	0	0	0	[illegible]
SM.B_Me_17389	Dicot. sp. indet.	12				0	0	0	0	0	[illegible]
SM.B_Me_1740	Dicot. sp. GM010	219				0	0	0	0	0	[illegible]
SM.B_Me_17410	Juglandaceae sp.	163				0	0	0	0	0	[illegible]
SM.B_Me_1744	Juglandaceae Formenkreis 1	142				0	0	0	0	0	[illegible]
SM.B_Me_17452	Juglandaceae sp.	14				0	0	0	0	0	[illegible]
SM.B_Me_1746	Tremophyllum sp.	12				0	0	0	0	0	[illegible]
SM.B_Me_17484	Dicot. sp. indet.	145				0	0	0	0	0	[illegible]
SM.B_Me_17502	Juglandaceae sp.	152				0	0	0	0	0	[illegible]
SM.B_Me_17503	Daphnogene sp.	2				0	1	0	0	0	[illegible]
SM.B_Me_1752	Dicot. sp. indet.	14				0	0	0	0	0	[illegible]
SM.B_Me_17559	Tremophyllum sp.	3	143			0	0	1	0	0	[illegible]
SM.B_Me_17562	Dicot. sp. indet.	80				0	0	0	0	0	[illegible]
SM.B_Me_17563	Juglandaceae Formenkreis 1	149				0	0	0	0	0	[illegible]
SM.B_Me_17566	Dicot. sp. indet.	195				0	0	0	0	0	[illegible]
SM.B_Me_17569	Juglandaceae sp.	12				0	0	0	0	0	[illegible]
SM.B_Me_1757	Toddalia ovata	171				0	0	0	0	0	[illegible]
SM.B_Me_17573	Juglandaceae Formenkreis 1	189				0	0	0	0	0	[illegible]
SM.B_Me_17576	Juglandaceae sp.	12				0	0	0	0	0	[illegible]
SM.B_Me_1758	Nyssa sp.	190	91			0	0	0	0	0	[illegible]
SM.B_Me_1761	Laurophyllum lanigeroides	19				0	0	0	0	0	[illegible]
SM.B_Me_17618	Lauraceae sp.	189				0	0	0	0	0	[illegible]
SM.B_Me_1763	Dicot. sp. indet.	208				0	0	0	0	0	[illegible]
SM.B_Me_17632	Laurophyllum lanigeroides	206				0	0	0	0	0	[illegible]
SM.B_Me_17635	Juglandaceae sp.	2				0	1	0	0	0	[illegible]
SM.B_Me_17638	Juglandaceae sp.	163				0	0	0	0	0	[illegible]
SM.B_Me_17639	Juglandaceae sp.	14				0	0	0	0	0	[illegible]
SM.B_Me_17641	Juglandaceae sp.	208				0	0	0	0	0	[illegible]
SM.B_Me_17647	Juglandaceae sp.	12				0	0	0	0	0	[illegible]
SM.B_Me_17670	Lauraceae sp.	12				0	0	0	0	0	[illegible]
SM.B_Me_17673	Dicot. sp. indet.	36	13			0	0	0	0	0	[illegible]
SM.B_Me_17675	Juglandaceae sp.	12				0	0	0	0	0	[illegible]
SM.B_Me_17676	Lauraceae sp.	12				0	0	0	0	0	[illegible]
SM.B_Me_17688	Juglandaceae Formenkreis 1	149				0	0	0	0	0	[illegible]
SM.B_Me_17755	Juglandaceae sp.	12				0	0	0	0	0	[illegible]
SM.B_Me_17763	Juglandaceae Formenkreis 1	190				0	0	0	0	0	[illegible]
SM.B_Me_17799	Juglandaceae Formenkreis 1	190				0	0	0	0	0	[illegible]
SM.B_Me_17803	Lauraceae sp.	12				0	0	0	0	0	[illegible]
SM.B_Me_17805	Lauraceae sp.	36				0	0	0	0	0	[illegible]
SM.B_Me_17810	Lauraceae sp.	3				0	0	1	0	0	[illegible]
SM.B_Me_17811	Lauraceae sp.	3				0	0	1	0	0	[illegible]
SM.B_Me_17818	Rhodomyrtophyllum sinuatum	189	36	12		0	0	0	0	0	[illegible]
SM.B_Me_1782	Dicot. sp. indet.	146				0	0	0	0	0	[illegible]
SM.B_Me_17867	Leguminosae sp. 5	12				0	0	0	0	0	[illegible]
SM.B_Me_1788	Dicot. sp. indet.	195				0	0	0	0	0	[illegible]
SM.B_Me_1789	Dicot. sp. indet.	190				0	0	0	0	0	[illegible]
SM.B_Me_17901	Daphnogene sp.	12				0	0	0	0	0	[illegible]
SM.B_Me_17906	Juglandaceae Formenkreis 2	12				0	0	0	0	0	[illegible]
SM.B_Me_17907	Dicot. sp. indet.	34				0	0	0	0	0	[illegible]
SM.B_Me_17915	Dicot. sp. indet.	210				0	0	0	0	0	[illegible]
SM.B_Me_17930	Juglandaceae sp.	2				0	1	0	0	0	[illegible]
SM.B_Me_17933	Laurophyllum sp.	189	12	13		0	0	0	0	0	[illegible]
SM.B_Me_17934	Juglandaceae Formenkreis 1	12	204			0	0	0	0	0	[illegible]
SM.B_Me_17940	Dicot. sp. indet.	195				0	0	0	0	0	[illegible]
SM.B_Me_17946	Acer sp.	12				0	0	0	0	0	[illegible]
SM.B_Me_17953	Juglandaceae Formenkreis 1	12				0	0	0	0	0	[illegible]
SM.B_Me_17955	Juglandaceae Formenkreis 2	208				0	0	0	0	0	[illegible]
SM.B_Me_17957	Juglandaceae Formenkreis 2	206	12			0	0	0	0	0	[illegible]
SM.B_Me_17961	Juglandaceae Formenkreis 2	208				0	0	0	0	0	[illegible]
SM.B_Me_17965	Dicot. sp. indet.	146				0	0	0	0	0	[illegible]
SM.B_Me_17970	Acer sp.	4				0	0	0	1	0	[illegible]
SM.B_Me_17974	Lauraceae sp.	3				0	0	1	0	0	[illegible]
SM.B_Me_17996	Leguminosae sp. 5	57				0	0	0	0	0	[illegible]
SM.B_Me_179a	Laurophyllum lanigeroides	216	2			0	1	0	0	0	[illegible]
SM.B_Me_180	Laurophyllum lanigeroides	206				0	0	0	0	0	[illegible]
SM.B_Me_18009	Juglandaceae sp.	22				0	0	0	0	0	[illegible]
SM.B_Me_18023	Dicot. sp. indet.	12				0	0	0	0	0	[illegible]
SM.B_Me_18025	Juglandaceae Formenkreis 2	12				0	0	0	0	0	[illegible]
SM.B_Me_18027	Juglandaceae Formenkreis 2	189				0	0	0	0	0	[illegible]
SM.B_Me_1805	Juglandaceae sp.	12				0	0	0	0	0	[illegible]
SM.B_Me_1806	Rhodomyrtophyllum sinuatum	12				0	0	0	0	0	[illegible]
SM.B_Me_18082	Dicot. sp. GM013	2				0	1	0	0	0	[illegible]
SM.B_Me_18086	Juglandaceae sp.	220	12			0	0	0	0	0	[illegible]
SM.B_Me_18102	Juglandaceae sp.	12				0	0	0	0	0	[illegible]
SM.B_Me_18119	Dicot. sp. indet.	76				0	0	0	0	0	[illegible]
SM.B_Me_1813	Dicot. sp. indet.	195				0	0	0	0	0	[illegible]
SM.B_Me_18140	Laurophyllum sp.	12				0	0	0	0	0	[illegible]
SM.B_Me_18152	Dicot. sp. indet.	210				0	0	0	0	0	[illegible]
SM.B_Me_18162	Juglandaceae sp.	149				0	0	0	0	0	[illegible]
SM.B_Me_18187	Dicot. sp. indet.	188	32			0	0	0	0	0	[illegible]
SM.B_Me_18190	Lauraceae sp.	119	76	12		0	0	0	0	0	[illegible]
SM.B_Me_18191	Lauraceae sp.	12				0	0	0	0	0	[illegible]
SM.B_Me_18192	Juglandaceae Formenkreis 2	12				0	0	0	0	0	[illegible]
SM.B_Me_18197	Lauraceae sp.	3				0	0	1	0	0	[illegible]
SM.B_Me_18200	Juglandaceae sp.	14				0	0	0	0	0	[illegible]
SM.B_Me_18201	Juglandaceae sp.	12				0	0	0	0	0	[illegible]
SM.B_Me_18208	Dicot. sp. indet.	12				0	0	0	0	0	[illegible]
SM.B_Me_18211	Laurophyllum lanigeroides	3				0	0	1	0	0	[illegible]
SM.B_Me_18215	Dicot. sp. indet.	80				0	0	0	0	0	[illegible]
SM.B_Me_18218	Lauraceae sp.	14				0	0	0	0	0	[illegible]
SM.B_Me_18220	Juglandaceae sp.	190				0	0	0	0	0	[illegible]
SM.B_Me_18221	Lauraceae sp.	62				0	0	0	0	0	[illegible]
SM.B_Me_18223	Juglandaceae sp.	163				0	0	0	0	0	[illegible]
SM.B_Me_18227	Lauraceae sp.	80				0	0	0	0	0	[illegible]
SM.B_Me_18239	Lauraceae sp.	146	3	12		0	0	1	0	0	[illegible]
SM.B_Me_18261	Dicot. sp. indet.	36				0	0	0	0	0	[illegible]
SM.B_Me_18269	Juglandaceae sp.	12				0	0	0	0	0	[illegible]
SM.B_Me_18309	Dicot. sp. indet.	189				0	0	0	0	0	[illegible]
SM.B_Me_1831	Laurophyllum sp.	12				0	0	0	0	0	[illegible]
SM.B_Me_18317	Juglandaceae sp.	12				0	0	0	0	0	[illegible]
SM.B_Me_18322	Laurophyllum lanigeroides	3				0	0	1	0	0	[illegible]
SM.B_Me_18324	Laurophyllum lanigeroides	12				0	0	0	0	0	[illegible]
SM.B_Me_18329	Lauraceae sp.	12				0	0	0	0	0	[illegible]
SM.B_Me_18331	Lauraceae sp.	3				0	0	1	0	0	[illegible]
SM.B_Me_18338	Lauraceae sp.	14				0	0	0	0	0	[illegible]
SM.B_Me_18342	Lauraceae sp.	3				0	0	1	0	0	[illegible]
SM.B_Me_18343	Lauraceae sp.	12				0	0	0	0	0	[illegible]
SM.B_Me_18350	Laurophyllum lanigeroides	146				0	0	0	0	0	[illegible]
SM.B_Me_18352	Laurophyllum sp.	12				0	0	0	0	0	[illegible]
SM.B_Me_18355	Daphnogene crebrigranosa	12				0	0	0	0	0	[illegible]
SM.B_Me_18356	Lauraceae sp.	12				0	0	0	0	0	[illegible]
SM.B_Me_1836	Juglandaceae Formenkreis 2	142	80			0	0	0	0	0	[illegible]
SM.B_Me_18365	Lauraceae sp.	145	12			0	0	0	0	0	[illegible]
SM.B_Me_18370	Ficus sp.	62	12			0	0	0	0	0	[illegible]
SM.B_Me_1841	Juglandaceae sp.	207				0	0	0	0	0	[illegible]
SM.B_Me_18418	Juglandaceae sp.	12				0	0	0	0	0	[illegible]
SM.B_Me_18432	Dicot. sp. indet.	145				0	0	0	0	0	[illegible]
SM.B_Me_18457	Dicot. sp. indet.	12				0	0	0	0	0	[illegible]
SM.B_Me_18459	Dicot. sp. indet.	57	2			0	1	0	0	0	[illegible]
SM.B_Me_18470	Dicot. sp. GM009	17				0	0	0	0	0	[illegible]
SM.B_Me_18472	Dicot. sp. indet.	195				0	0	0	0	0	[illegible]
SM.B_Me_18475	Dicot. sp. indet.	200	12			0	0	0	0	0	[illegible]
SM.B_Me_18477	Lauraceae sp.	3	33			0	0	1	0	0	[illegible]
SM.B_Me_18478	Lauraceae sp.	2				0	1	0	0	0	[illegible]
SM.B_Me_18483	Laurophyllum lanigeroides	163				0	0	0	0	0	[illegible]

Wappler et al SOM File S1

SM.B_Me_18487	Dicot. sp. indet.	195				0	0	0	0	0	[illegible]
SM.B_Me_18492	Dicot. sp. GM003	195				0	0	0	0	0	[illegible]
SM.B_Me_18500	Dicot. sp. indet.	16				0	0	0	0	0	[illegible]
SM.B_Me_18532	Tremophyllum sp.	195				0	0	0	0	0	[illegible]
SM.B_Me_18539	Tremophyllum sp.	5				0	0	0	0	1	[illegible]
SM.B_Me_1854	Dicot. sp. indet.	208				0	0	0	0	0	[illegible]
SM.B_Me_18545	Dicot. sp. indet.	36				0	0	0	0	0	[illegible]
SM.B_Me_1857	Juglandaceae sp.	2				0	1	0	0	0	[illegible]
SM.B_Me_18570	Laurophyllum lanigeroides	30				0	0	0	0	0	[illegible]
SM.B_Me_18576	Lauraceae sp.	3				0	0	1	0	0	[illegible]
SM.B_Me_18591	Dicot. sp. indet.	195				0	0	0	0	0	[illegible]
SM.B_Me_18619	Dicot. sp. indet.	12				0	0	0	0	0	[illegible]
[illegible]	Ficus sp.	2				0	1	0	0	0	[illegible]
SM.B_Me_18630	? Laurophyllum sp.	12	2			0	1	0	0	0	[illegible]
SM.B_Me_18668	Ficus sp.	169	12			0	0	0	0	0	[illegible]
SM.B_Me_18671	Dicot. sp. indet.	195				0	0	0	0	0	[illegible]
SM.B_Me_1869	Juglandaceae sp.	12				0	0	0	0	0	[illegible]
SM.B_Me_18696	Juglandaceae sp.	80				0	0	0	0	0	[illegible]
SM.B_Me_18701	Juglandaceae sp.	2				0	1	0	0	0	[illegible]
SM.B_Me_18705	Dicot. sp. indet.	208	12	3		0	0	1	0	0	[illegible]
SM.B_Me_18714	Lauraceae sp.	14	13			0	0	0	0	0	[illegible]
SM.B_Me_18716	Lauraceae sp.	36				0	0	0	0	0	[illegible]
SM.B_Me_18725	Lauraceae sp.	17				0	0	0	0	0	[illegible]
SM.B_Me_18728	Dicot. sp. indet.	12				0	0	0	0	0	[illegible]
SM.B_Me_18729	Lauraceae sp.	12				0	0	0	0	0	[illegible]
SM.B_Me_18730	Dicot. sp. indet.	12				0	0	0	0	0	[illegible]
SM.B_Me_18732	Juglandaceae sp.	12				0	0	0	0	0	[illegible]
SM.B_Me_18734	Daphnogene sp.	201				0	0	0	0	0	[illegible]
SM.B_Me_18735	Nymphaeaceae sp.	49				0	0	0	0	0	[illegible]
SM.B_Me_18736	Nymphaeaceae sp.	49				0	0	0	0	0	[illegible]
SM.B_Me_18737	Nymphaeaceae sp.	49				0	0	0	0	0	[illegible]
SM.B_Me_18740	Vitaceae sp.	171	90			0	0	0	0	0	[illegible]
SM.B_Me_18744	Nymphaeaceae sp.	49				0	0	0	0	0	[illegible]
SM.B_Me_18746	Juglandaceae sp.	12				0	0	0	0	0	[illegible]
SM.B_Me_18761	Dicot. sp. indet.	200				0	0	0	0	0	[illegible]
SM.B_Me_18764	Laurophyllum sp.	145				0	0	0	0	0	[illegible]
SM.B_Me_18775	Dicot. sp. indet.	197	12			0	0	0	0	0	[illegible]
SM.B_Me_1878	Laurophyllum lanigeroides	145	163			0	0	0	0	0	[illegible]
SM.B_Me_18782	Vitaceae sp.	12				0	0	0	0	0	[illegible]
SM.B_Me_18787	Lauraceae sp.	195				0	0	0	0	0	[illegible]
SM.B_Me_18798	Lauraceae sp.	37	206	3		0	0	1	0	0	[illegible]
SM.B_Me_18806	Lauraceae sp.	3				0	0	1	0	0	[illegible]
SM.B_Me_18812	Laurophyllum sp.	171	3			0	0	1	0	0	[illegible]
SM.B_Me_18823	Lauraceae sp.	145				0	0	0	0	0	[illegible]
SM.B_Me_18824	Dicot. sp. indet.	12				0	0	0	0	0	[illegible]
SM.B_Me_18825	Dicot. sp. indet.	200				0	0	0	0	0	[illegible]
SM.B_Me_18828	Daphnogene crebrigranosa	14	13			0	0	0	0	0	[illegible]
SM.B_Me_18832	Juglandaceae sp.	12	2			0	1	0	0	0	[illegible]
SM.B_Me_18833	Dicot. sp. indet.	208				0	0	0	0	0	[illegible]
SM.B_Me_18835	Lauraceae sp.	14				0	0	0	0	0	[illegible]
SM.B_Me_18839	Lauraceae sp.	12				0	0	0	0	0	[illegible]
SM.B_Me_18846	? Illiciaceae sp.	14				0	0	0	0	0	[illegible]
SM.B_Me_18847	Lauraceae sp.	12				0	0	0	0	0	[illegible]
SM.B_Me_18852	Juglandaceae sp.	12				0	0	0	0	0	[illegible]
SM.B_Me_18885	Leguminosae sp. 5	12				0	0	0	0	0	[illegible]
SM.B_Me_1891	Dicot. sp. indet.	12				0	0	0	0	0	[illegible]
SM.B_Me_18931	Lauraceae sp.	14				0	0	0	0	0	[illegible]
SM.B_Me_18934	Juglandaceae sp.	12				0	0	0	0	0	[illegible]
SM.B_Me_18937	Lauraceae sp.	145	12			0	0	0	0	0	[illegible]
SM.B_Me_18943	Dicot. sp. indet.	195	12			0	0	0	0	0	[illegible]
SM.B_Me_1895	Nyssa sp.	30				0	0	0	0	0	[illegible]
SM.B_Me_18957	Dicot. sp. indet.	85				0	0	0	0	0	[illegible]
SM.B_Me_1902	Laurophyllum sp.	14				0	0	0	0	0	[illegible]
SM.B_Me_19021	Leguminosae sp. 5	12				0	0	0	0	0	[illegible]
SM.B_Me_19030	Dicot. sp. indet.	12				0	0	0	0	0	[illegible]
SM.B_Me_19032	Dicot. sp. indet.	12				0	0	0	0	0	[illegible]
SM.B_Me_19037	Dicot. sp. indet.	3	12			0	0	1	0	0	[illegible]
SM.B_Me_1904	Juglandaceae Formenkreis 2	12				0	0	0	0	0	[illegible]
SM.B_Me_19042	Juglandaceae sp.	14				0	0	0	0	0	[illegible]
SM.B_Me_19045	Dicot. sp. indet.	12				0	0	0	0	0	[illegible]
SM.B_Me_19046	Juglandaceae sp.	163	12			0	0	0	0	0	[illegible]
SM.B_Me_19048	Lauraceae sp.	3				0	0	1	0	0	[illegible]
SM.B_Me_19051	Dicot. sp. indet.	12				0	0	0	0	0	[illegible]
SM.B_Me_19053	Dicot. sp. indet.	195				0	0	0	0	0	[illegible]
SM.B_Me_19054	Laurophyllum lanigeroides	146				0	0	0	0	0	[illegible]
SM.B_Me_19056	Laurophyllum sp.	3				0	0	1	0	0	[illegible]
SM.B_Me_19059	Juglandaceae sp.	12				0	0	0	0	0	[illegible]
SM.B_Me_1906	Dicot. sp. indet.	2				0	1	0	0	0	[illegible]
SM.B_Me_19065	Dicot. sp. indet.	12				0	0	0	0	0	[illegible]
SM.B_Me_19066	Laurophyllum sp.	206	30			0	0	0	0	0	[illegible]
SM.B_Me_19069	Daphnogene sp.	14				0	0	0	0	0	[illegible]
SM.B_Me_1907	Dicot. sp. indet.	220	1			1	0	0	0	0	[illegible]
SM.B_Me_19072	Juglandaceae sp.	12				0	0	0	0	0	[illegible]
SM.B_Me_19074	Dicot. sp. GM013	3				0	0	1	0	0	[illegible]
SM.B_Me_19079	Lauraceae sp.	3	13			0	0	1	0	0	[illegible]
SM.B_Me_19083	Laurophyllum sp.	3				0	0	1	0	0	[illegible]
SM.B_Me_19086	Juglandaceae sp.	12				0	0	0	0	0	[illegible]
SM.B_Me_19089	Juglandaceae sp.	12				0	0	0	0	0	[illegible]
SM.B_Me_19092	Laurophyllum lanigeroides	12	2			0	1	0	0	0	[illegible]
SM.B_Me_19096	Juglandaceae sp.	15				0	0	0	0	0	[illegible]
SM.B_Me_19099	Dicot. sp. indet.	12				0	0	0	0	0	[illegible]
SM.B_Me_19103	Dicot. sp. indet.	188				0	0	0	0	0	[illegible]
SM.B_Me_19106	Dicot. sp. indet.	2				0	1	0	0	0	[illegible]
SM.B_Me_19107	Juglandaceae sp.	14				0	0	0	0	0	[illegible]
SM.B_Me_19116	Juglandaceae sp.	12				0	0	0	0	0	[illegible]
SM.B_Me_19119	Dicot. sp. indet.	146				0	0	0	0	0	[illegible]
SM.B_Me_19140	Leguminosae sp. 5	210				0	0	0	0	0	[illegible]
SM.B_Me_19143	Juglandaceae sp.	163				0	0	0	0	0	[illegible]
SM.B_Me_19145	Juglandaceae sp.	163	12			0	0	0	0	0	[illegible]
SM.B_Me_19164	Dicot. sp. indet.	142				0	0	0	0	0	[illegible]
SM.B_Me_19165	Dicot. sp. indet.	142				0	0	0	0	0	[illegible]
SM.B_Me_19167	Juglandaceae sp.	12				0	0	0	0	0	[illegible]
SM.B_Me_1919	Laurophyllum lanigeroides	12				0	0	0	0	0	[illegible]
SM.B_Me_19219	Juglandaceae sp.	15				0	0	0	0	0	[illegible]
SM.B_Me_19221	Ficus sp.	34				0	0	0	0	0	[illegible]
SM.B_Me_19227	Dicot. sp. indet.	76				0	0	0	0	0	[illegible]
SM.B_Me_19233	Juglandaceae sp.	12				0	0	0	0	0	[illegible]
SM.B_Me_19240	Laurophyllum lanigeroides	12				0	0	0	0	0	[illegible]
SM.B_Me_19245	Dicot. sp. indet.	14	12			0	0	0	0	0	[illegible]
SM.B_Me_19246	Juglandaceae sp.	109	14			0	0	0	0	0	[illegible]
SM.B_Me_19249	Laurophyllum sp.	62				0	0	0	0	0	[illegible]
SM.B_Me_19250	Dicot. sp. indet.	191				0	0	0	0	0	[illegible]
SM.B_Me_1926	Ericaceae sp.	163				0	0	0	0	0	[illegible]
SM.B_Me_19260	Dicot. sp. indet.	195				0	0	0	0	0	[illegible]
SM.B_Me_1927	Dicot. sp. indet.	76				0	0	0	0	0	[illegible]
SM.B_Me_1928	Ficus sp.	208				0	0	0	0	0	[illegible]
SM.B_Me_1929	Laurophyllum lanigeroides	187				0	0	0	0	0	[illegible]
SM.B_Me_19309	Dicot. sp. indet.	191				0	0	0	0	0	[illegible]
SM.B_Me_19311	Juglandaceae sp.	12				0	0	0	0	0	[illegible]
SM.B_Me_19312	Daphnogene sp.	12				0	0	0	0	0	[illegible]
SM.B_Me_19331	Dicot. sp. indet.	187				0	0	0	0	0	[illegible]
SM.B_Me_19347	Dicot. sp. indet.	195				0	0	0	0	0	[illegible]
SM.B_Me_19348	Dicot. sp. indet.	36				0	0	0	0	0	[illegible]
SM.B_Me_19355	Juglandaceae sp.	4				0	0	0	1	0	[illegible]
SM.B_Me_19356	Juglandaceae sp.	149	216	32	14	0	0	0	0	0	[illegible]
SM.B_Me_19357	Laurophyllum sp.	218	32			0	0	0	0	0	[illegible]
SM.B_Me_19358	Laurophyllum sp.	3	17			0	0	1	0	0	[illegible]
SM.B_Me_1936	Dicot. sp. indet.	190				0	0	0	0	0	[illegible]
SM.B_Me_19362	Lauraceae sp.	145				0	0	0	0	0	[illegible]
SM.B_Me_19367	Lauraceae sp.	12				0	0	0	0	0	[illegible]
SM.B_Me_1937	Daphnogene sp.	15				0	0	0	0	0	[illegible]
SM.B_Me_19370	Dicot. sp. indet.	36				0	0	0	0	0	[illegible]
SM.B_Me_19371	Laurophyllum sp.	145				0	0	0	0	0	[illegible]
SM.B_Me_19378	Juglandaceae sp.	195	14			0	0	0	0	0	[illegible]
SM.B_Me_19380	Dicot. sp. indet.	12				0	0	0	0	0	[illegible]
SM.B_Me_19383	Laurophyllum sp.	12				0	0	0	0	0	[illegible]
SM.B_Me_19391	Laurophyllum lanigeroides	14				0	0	0	0	0	[illegible]
SM.B_Me_19392	Juglandaceae sp.	163				0	0	0	0	0	[illegible]
SM.B_Me_19394	Lauraceae sp.	145				0	0	0	0	0	[illegible]
SM.B_Me_19400	Juglandaceae sp.	78				0	0	0	0	0	[illegible]
SM.B_Me_1945	Lauraceae sp.	189				0	0	0	0	0	[illegible]
SM.B_Me_19480	Juglandaceae sp.	12				0	0	0	0	0	[illegible]
SM.B_Me_1951	Juglandaceae Formenkreis 1	149				0	0	0	0	0	[illegible]
SM.B_Me_1957	Dicot. sp. GM007	216				0	0	0	0	0	[illegible]
SM.B_Me_19577	Lauraceae sp.	14				0	0	0	0	0	[illegible]
SM.B_Me_19578	Juglandaceae sp.	12				0	0	0	0	0	[illegible]
SM.B_Me_19590	Dicot. sp. indet.	2				0	1	0	0	0	[illegible]
SM.B_Me_19604	Juglandaceae sp.	12				0	0	0	0	0	[illegible]
SM.B_Me_19605	Dicot. sp. indet.	195				0	0	0	0	0	[illegible]
SM.B_Me_19608	Dicot. sp. indet.	195				0	0	0	0	0	[illegible]
SM.B_Me_19613	Juglandaceae sp.	2				0	1	0	0	0	[illegible]
SM.B_Me_19617	Dicot. sp. indet.	4				0	0	0	1	0	[illegible]
SM.B_Me_19621	Laurophyllum sp.	206	145			0	0	0	0	0	[illegible]
SM.B_Me_19633	Dicot. sp. indet.	16				0	0	0	0	0	[illegible]
SM.B_Me_19637	Laurophyllum sp.	3				0	0	1	0	0	[illegible]
SM.B_Me_19640	Juglandaceae sp.	12				0	0	0	0	0	[illegible]
SM.B_Me_19644	Laurophyllum sp.	146	12			0	0	0	0	0	[illegible]
SM.B_Me_19646	Lauraceae sp.	163	218			0	0	0	0	0	[illegible]
SM.B_Me_19648	Juglandaceae sp.	12				0	0	0	0	0	[illegible]
SM.B_Me_19650	Laurophyllum sp.	145				0	0	0	0	0	[illegible]
SM.B_Me_19677	Lauraceae sp.	163				0	0	0	0	0	[illegible]
SM.B_Me_19679	Lauraceae sp.	62				0	0	0	0	0	[illegible]
SM.B_Me_19698	Juglandaceae sp.	189	92			0	0	0	0	0	[illegible]
SM.B_Me_19702	Dicot. sp. indet.	195				0	0	0	0	0	[illegible]
SM.B_Me_19714	Magnoliaceae sp.	195				0	0	0	0	0	[illegible]
SM.B_Me_1973	? Vitaceae / ? Menispermaceae / ? Acer	187				0	0	0	0	0	[illegible]
SM.B_Me_19733	Laurophyllum sp.	145				0	0	0	0	0	[illegible]
SM.B_Me_19743	Juglandaceae sp.	163				0	0	0	0	0	[illegible]
SM.B_Me_19752	Dicot. sp. indet.	5				0	0	0	0	1	[illegible]
SM.B_Me_19764	Dicot. sp. indet.	189				0	0	0	0	0	[illegible]
SM.B_Me_19787	Lauraceae sp.	12				0	0	0	0	0	[illegible]
SM.B_Me_19794	Lauraceae sp.	62				0	0	0	0	0	[illegible]
SM.B_Me_19796	Lauraceae sp.	12				0	0	0	0	0	[illegible]
SM.B_Me_19807	Lauraceae sp.	54				0	0	0	0	0	[illegible]
SM.B_Me_19826	Laurophyllum lanigeroides	3				0	0	1	0	0	[illegible]
SM.B_Me_19830	Juglandaceae Formenkreis 1	190				0	0	0	0	0	[illegible]
SM.B_Me_19831	Lauraceae sp.	12				0	0	0	0	0	[illegible]
SM.B_Me_19891	Dicot. sp. indet.	14				0	0	0	0	0	[illegible]
SM.B_Me_1992	Laurophyllum hirsutum	142				0	0	0	0	0	[illegible]
SM.B_Me_1994	? Laurophyllum sp.	62				0	0	0	0	0	[illegible]
SM.B_Me_19944	Dicot. sp. GM013	213				0	0	0	0	0	[illegible]
SM.B_Me_1996	Tremophyllum sp.	198				0	0	0	0	0	[illegible]
SM.B_Me_19997	Juglandaceae Formenkreis 1	163				0	0	0	0	0	[illegible]
SM.B_Me_20019	Juglandaceae Formenkreis 1	12				0	0	0	0	0	[illegible]
SM.B_Me_20024	Juglandaceae Formenkreis 1	12				0	0	0	0	0	[illegible]
SM.B_Me_20037	Juglandaceae sp.	12				0	0	0	0	0	[illegible]
SM.B_Me_20093	Dicot. sp. indet.	36				0	0	0	0	0	[illegible]
SM.B_Me_20188	Juglandaceae sp.	163				0	0	0	0	0	[illegible]
SM.B_Me_20220	Dicot. sp. indet.	189				0	0	0	0	0	[illegible]
SM.B_Me_20396	Tremophyllum sp.	36				0	0	0	0	0	[illegible]
SM.B_Me_20399	Juglandaceae sp.	14				0	0	0	0	0	[illegible]
SM.B_Me_20404	Dicot. sp. indet.	210				0	0	0	0	0	[illegible]
SM.B_Me_20414	Lauraceae sp.	146	12			0	0	0	0	0	[illegible]
SM.B_Me_20421	Juglandaceae sp.	12				0	0	0	0	0	[illegible]
SM.B_Me_20426	Leguminosae sp. 5	191				0	0	0	0	0	[illegible]
SM.B_Me_20436	Laurophyllum sp.	13				0	0	0	0	0	[illegible]
SM.B_Me_20450	Dicot. sp. indet.	195				0	0	0	0	0	[illegible]
SM.B_Me_20451	Lauraceae sp.	12				0	0	0	0	0	[illegible]
SM.B_Me_20458	Juglandaceae Formenkreis 1	208				0	0	0	0	0	[illegible]
SM.B_Me_20459	Juglandaceae sp.	12				0	0	0	0	0	[illegible]
SM.B_Me_20472	Juglandaceae sp.	12				0	0	0	0	0	[illegible]
SM.B_Me_20480	Dicot. sp. indet.	13				0	0	0	0	0	[illegible]
SM.B_Me_20489	Dicot. sp. indet.	13				0	0	0	0	0	[illegible]
SM.B_Me_20491	Dicot. sp. indet.	2				0	1	0	0	0	[illegible]
SM.B_Me_20494	Dicot. sp. indet.	16				0	0	0	0	0	[illegible]
SM.B_Me_20495	Lauraceae sp.	142				0	0	0	0	0	[illegible]
SM.B_Me_20496	Juglandaceae Formenkreis 1	163				0	0	0	0	0	[illegible]
SM.B_Me_20498	Juglandaceae Formenkreis 1	163	12			0	0	0	0	0	[illegible]
SM.B_Me_20500	Dicot. sp. indet.	14				0	0	0	0	0	[illegible]
SM.B_Me_20505	Dicot. sp. indet.	12				0	0	0	0	0	[illegible]
SM.B_Me_20708	Dicot. sp. indet.	30				0	0	0	0	0	[illegible]
SM.B_Me_20710	Vitaceae sp.	12				0	0	0	0	0	[illegible]
SM.B_Me_20730	Dicot. sp. indet.	2				0	1	0	0	0	[illegible]
SM.B_Me_20732	Dicot. sp. indet.	36				0	0	0	0	0	[illegible]
SM.B_Me_20747	Juglandaceae sp.	12				0	0	0	0	0	[illegible]
SM.B_Me_20760	Dicot. sp. GM003	208				0	0	0	0	0	[illegible]
SM.B_Me_20761	Ficus sp.	12				0	0	0	0	0	[illegible]
SM.B_Me_208	Laurophyllum lanigeroides	206				0	0	0	0	0	[illegible]
SM.B_Me_20923	Ulmaceae sp.	12				0	0	0	0	0	[illegible]
SM.B_Me_21007	Juglandaceae sp.	163				0	0	0	0	0	[illegible]
SM.B_Me_21074	Laurophyllum sp.	17				0	0	0	0	0	[illegible]
SM.B_Me_21075	Ficus sp.	213				0	0	0	0	0	[illegible]
SM.B_Me_21089	Juglandaceae sp.	12				0	0	0	0	0	[illegible]
SM.B_Me_21091	Dicot. sp. indet.	12				0	0	0	0	0	[illegible]
SM.B_Me_21093	Laurophyllum lanigeroides	12				0	0	0	0	0	[illegible]
SM.B_Me_21094	Juglandaceae Formenkreis 1	189				0	0	0	0	0	[illegible]
SM.B_Me_211	Dicot. sp. GM007	33				0	0	0	0	0	[illegible]
SM.B_Me_21101	Laurophyllum lanigeroides	145				0	0	0	0	0	[illegible]
SM.B_Me_21104	Leguminosae sp. 5	147				0	0	0	0	0	[illegible]
SM.B_Me_21106	Dicot. sp. indet.	12				0	0	0	0	0	[illegible]
SM.B_Me_21128	Juglandaceae sp.	206	14			0	0	0	0	0	[illegible]
SM.B_Me_21141	Juglandaceae Formenkreis 1	190				0	0	0	0	0	[illegible]
SM.B_Me_21143	Juglandaceae sp.	163				0	0	0	0	0	[illegible]
SM.B_Me_21155	Juglandaceae sp.	14				0	0	0	0	0	[illegible]
SM.B_Me_21172	Dicot. sp. indet.	16				0	0	0	0	0	[illegible]
SM.B_Me_21173	Leguminosae sp. 5	62	3			0	0	1	0	0	[illegible]
SM.B_Me_21174	Lauraceae sp.	62				0	0	0	0	0	[illegible]
SM.B_Me_21175	? Myricaceae / ? Juglandaceae	117				0	0	0	0	0	[illegible]
SM.B_Me_21178	Dicot. sp. indet.	81				0	0	0	0	0	[illegible]
SM.B_Me_21179	Dicot. sp. GM003	36				0	0	0	0	0	[illegible]
SM.B_Me_21180	Laurophyllum lanigeroides	206				0	0	0	0	0	[illegible]
SM.B_Me_21181	? Illiciaceae sp.	80	12			0	0	0	0	0	[illegible]
SM.B_Me_21182	Dicot. sp. indet.	54				0	0	0	0	0	[illegible]
SM.B_Me_21183	Dicot. sp. indet.	213	12	15		0	0	0	0	0	[illegible]
SM.B_Me_21184	Laurophyllum lanigeroides	148	13	206		0	0	0	0	0	[illegible]
SM.B_Me_21186	Laurophyllum lanigeroides	206				0	0	0	0	0	[illegible]
SM.B_Me_21187	Laurophyllum lanigeroides	189	34			0	0	0	0	0	[illegible]
SM.B_Me_21188	Lauraceae sp.	208				0	0	0	0	0	[illegible]
SM.B_Me_21189	Lauraceae sp.	145				0	0	0	0	0	[illegible]
SM.B_Me_21190	Laurophyllum sp.	188	3			0	0	1	0	0	[illegible]
SM.B_Me_21191	Leguminosae sp. 5	30				0	0	0	0	0	[illegible]
SM.B_Me_21193	Juglandaceae sp.	12				0	0	0	0	0	[illegible]
SM.B_Me_21196	Juglandaceae Formenkreis 1	12				0	0	0	0	0	[illegible]
SM.B_Me_21197	Leguminosae sp. 5	14	12			0	0	0	0	0	[illegible]
SM.B_Me_21199	Daphnogene crebrigranosa	12				0	0	0	0	0	[illegible]
SM.B_Me_21202	Juglandaceae Formenkreis 4	17				0	0	0	0	0	[illegible]
SM.B_Me_21204	Juglandaceae Formenkreis 1	80	31			0	0	0	0	0	[illegible]
SM.B_Me_21205	Juglandaceae Formenkreis 4	14	12			0	0	0	0	0	[illegible]
SM.B_Me_21206	Lauraceae sp.	142				0	0	0	0	0	[illegible]
SM.B_Me_21210	Juglandaceae sp.	12				0	0	0	0	0	[illegible]
SM.B_Me_21212	Dicot. sp. GM014	195				0	0	0	0	0	[illegible]
SM.B_Me_21215	Lauraceae sp.	197				0	0	0	0	0	[illegible]
SM.B_Me_21223	Lauraceae sp.	30				0	0	0	0	0	[illegible]
SM.B_Me_21224	Rosaceae sp.	12				0	0	0	0	0	[illegible]
SM.B_Me_21229	Juglandaceae sp.	12				0	0	0	0	0	[illegible]
SM.B_Me_21231	Juglandaceae Formenkreis 1	36				0	0	0	0	0	[illegible]
SM.B_Me_21232	Daphnogene sp. 2	30				0	0	0	0	0	[illegible]
SM.B_Me_21236	Leguminosae sp. 5	12				0	0	0	0	0	[illegible]
SM.B_Me_21245	Daphnogene sp.	12				0	0	0	0	0	[illegible]
SM.B_Me_21246	Rhodomyrtophyllum sinuatum	16				0	0	0	0	0	[illegible]
SM.B_Me_21247	? Anonaceae / ? Illicaceae	195				0	0	0	0	0	[illegible]
SM.B_Me_21248	? Anonaceae / ? Illicaceae	119				0	0	0	0	0	[illegible]
SM.B_Me_21258	Laurophyllum lanigeroides	81	12	3		0	0	1	0	0	[illegible]
SM.B_Me_21259	Laurophyllum sp.	81				0	0	0	0	0	[illegible]
SM.B_Me_229	Laurophyllum sp.	12				0	0	0	0	0	[illegible]
SM.B_Me_232	Dicot. sp. indet.	16				0	0	0	0	0	[illegible]
SM.B_Me_238	Lauraceae sp.	32				0	0	0	0	0	[illegible]
SM.B_Me_239	Laurophyllum sp.	34				0	0	0	0	0	[illegible]
SM.B_Me_24	Lauraceae sp.	12				0	0	0	0	0	[illegible]
SM.B_Me_241	Laurophyllum sp.	171				0	0	0	0	0	[illegible]
SM.B_Me_248	Dicot. sp. indet.	5				0	0	0	0	1	[illegible]
SM.B_Me_25	Daphnogene crebrigranosa	14				0	0	0	0	0	[illegible]
SM.B_Me_250	Acer sp.	12				0	0	0	0	0	[illegible]
SM.B_Me_2742	Dicot. sp. indet.	54				0	0	0	0	0	[illegible]
SM.B_Me_2744	Laurophyllum lanigeroides	145				0	0	0	0	0	[illegible]
SM.B_Me_2746	Rhodomyrtophyllum sinuatum	164				0	0	0	0	0	[illegible]
SM.B_Me_2747	Dicot. sp. indet.	4				0	0	0	1	0	[illegible]
SM.B_Me_2749	Dicot. sp. indet.	109				0	0	0	0	0	[illegible]
SM.B_Me_2750	Dicot. sp. indet.	109				0	0	0	0	0	[illegible]
SM.B_Me_2752	Dicot. sp. indet.	145				0	0	0	0	0	[illegible]
SM.B_Me_2753	Dicot. sp. GM008	32				0	0	0	0	0	[illegible]
SM.B_Me_3013	Laurophyllum lanigeroides	12	13			0	0	0	0	0	[illegible]
SM.B_Me_3023	Dicot. sp. indet.	85				0	0	0	0	0	[illegible]
SM.B_Me_3024	Laurophyllum lanigeroides	206				0	0	0	0	0	[illegible]
SM.B_Me_3027	Daphnogene sp.	145				0	0	0	0	0	[illegible]
SM.B_Me_3034	Juglandaceae Formenkreis 1	12				0	0	0	0	0	[illegible]
SM.B_Me_3051	Daphnogene sp.	12				0	0	0	0	0	[illegible]
SM.B_Me_3054	Dicot. sp. indet.	12				0	0	0	0	0	[illegible]
SM.B_Me_3059	Juglandaceae sp.	189				0	0	0	0	0	[illegible]
SM.B_Me_3063	Dicot. sp. indet.	5				0	0	0	0	1	[illegible]
SM.B_Me_3065	Leguminosae sp. 5	12				0	0	0	0	0	[illegible]
SM.B_Me_3077	Juglandaceae sp.	12				0	0	0	0	0	[illegible]
SM.B_Me_308	Vitaceae sp.	30				0	0	0	0	0	[illegible]
SM.B_Me_3084	Leguminosae sp. 5	12				0	0	0	0	0	[illegible]
SM.B_Me_3088	Laurophyllum lanigeroides	206	142			0	0	0	0	0	[illegible]
SM.B_Me_3091	Myricaceae sp.	14				0	0	0	0	0	[illegible]
SM.B_Me_3092	Laurophyllum lanigeroides	205				0	0	0	0	0	[illegible]
SM.B_Me_3094	Laurophyllum lanigeroides	206				0	0	0	0	0	[illegible]
SM.B_Me_3095	Juglandaceae Formenkreis 2	163	80			0	0	0	0	0	[illegible]
SM.B_Me_3101	Juglandaceae sp.	119				0	0	0	0	0	[illegible]
SM.B_Me_3107	Dicot. sp. indet.	12				0	0	0	0	0	[illegible]
SM.B_Me_3108	Juglandaceae Formenkreis 2	200	13	12		0	0	0	0	0	[illegible]
SM.B_Me_3109	Laurophyllum lanigeroides	206	12			0	0	0	0	0	[illegible]
SM.B_Me_3112	Juglandaceae sp.	54				0	0	0	0	0	[illegible]
SM.B_Me_3117	Juglandaceae sp.	14				0	0	0	0	0	[illegible]
SM.B_Me_3118	Laurophyllum lanigeroides	146				0	0	0	0	0	[illegible]
SM.B_Me_3122	? Illiciaceae sp.	222	12			0	0	0	0	0	[illegible]
SM.B_Me_3124	Laurophyllum lanigeroides	206	13			0	0	0	0	0	[illegible]
SM.B_Me_3126	Daphnogene sp. 2	30				0	0	0	0	0	[illegible]
SM.B_Me_314	Daphnogene sp.	12				0	0	0	0	0	[illegible]
SM.B_Me_3140	Juglandaceae Formenkreis 1	208				0	0	0	0	0	[illegible]
SM.B_Me_3146	Leguminosae sp. 5	147				0	0	0	0	0	[illegible]
SM.B_Me_3153	Juglandaceae sp.	2	12			0	1	0	0	0	[illegible]
SM.B_Me_3154	Dicot. sp. GM005	13				0	0	0	0	0	[illegible]
SM.B_Me_3155	Dicot. sp. indet.	12				0	0	0	0	0	[illegible]
SM.B_Me_316	Dicot. sp. indet.	2				0	1	0	0	0	[illegible]
SM.B_Me_3166	Laurophyllum lanigeroides	30				0	0	0	0	0	[illegible]
SM.B_Me_3168	Leguminosae sp. 5	12				0	0	0	0	0	[illegible]
SM.B_Me_3177	? Laurophyllum sp.	62				0	0	0	0	0	[illegible]
SM.B_Me_3183	Juglandaceae Formenkreis 1	12				0	0	0	0	0	[illegible]
SM.B_Me_3184	Laurophyllum lanigeroides	206	12			0	0	0	0	0	[illegible]
SM.B_Me_3186	Dicot. sp. GM015	3				0	0	1	0	0	[illegible]
SM.B_Me_3191	Dicot. sp. indet.	14				0	0	0	0	0	[illegible]
SM.B_Me_3198	Laurophyllum lanigeroides	146	12	163		0	0	0	0	0	[illegible]
SM.B_Me_3202	Leguminosae sp. 5	13				0	0	0	0	0	[illegible]
SM.B_Me_3204	Juglandaceae Formenkreis 1	195				0	0	0	0	0	[illegible]
SM.B_Me_3205	Tremophyllum sp.	8				0	0	0	0	0	[illegible]
SM.B_Me_3207	Dicot. sp. indet.	12				0	0	0	0	0	[illegible]
SM.B_Me_3213	Juglandaceae sp.	36				0	0	0	0	0	[illegible]
SM.B_Me_3214	Dicot. sp. indet.	36				0	0	0	0	0	[illegible]
SM.B_Me_3217	Leguminosae sp. 5	12				0	0	0	0	0	[illegible]
SM.B_Me_3220	Laurophyllum lanigeroides	206				0	0	0	0	0	[illegible]
SM.B_Me_3221	Laurophyllum lanigeroides	12				0	0	0	0	0	[illegible]
SM.B_Me_3223	Laurophyllum lanigeroides	12				0	0	0	0	0	[illegible]
SM.B_Me_3224	Dicot. sp. indet.	163	16			0	0	0	0	0	[illegible]
SM.B_Me_3229	Dicot. sp. indet.	12				0	0	0	0	0	[illegible]
SM.B_Me_3238	Juglandaceae sp.	213				0	0	0	0	0	[illegible]
SM.B_Me_3250	Dicot. sp. indet.	16				0	0	0	0	0	[illegible]
SM.B_Me_3256	Dicot. sp. indet.	12				0	0	0	0	0	[illegible]
SM.B_Me_3258	Sloanea sp.	30				0	0	0	0	0	[illegible]
SM.B_Me_3259	Laurophyllum sp.	195				0	0	0	0	0	[illegible]
SM.B_Me_3263	Laurophyllum lanigeroides	206	198			0	0	0	0	0	[illegible]
SM.B_Me_3264	? Illiciaceae sp.	185				0	0	0	0	0	[illegible]
SM.B_Me_3267	Laurophyllum lanigeroides	206				0	0	0	0	0	[illegible]
SM.B_Me_3269	Dicot. sp. indet.	188				0	0	0	0	0	[illegible]
SM.B_Me_3272	Laurophyllum lanigeroides	3				0	0	1	0	0	[illegible]
SM.B_Me_3275	Laurophyllum lanigeroides	3				0	0	1	0	0	[illegible]
SM.B_Me_3281	Laurophyllum lanigeroides	12				0	0	0	0	0	[illegible]
SM.B_Me_3285	Laurophyllum lanigeroides	3	15			0	0	1	0	0	[illegible]
SM.B_Me_3290	Juglandaceae sp.	12				0	0	0	0	0	[illegible]
SM.B_Me_3292	Juglandaceae Formenkreis 1	195				0	0	0	0	0	[illegible]
SM.B_Me_3298	Rhodomyrtophyllum sinuatum	79				0	0	0	0	0	[illegible]
SM.B_Me_3305	Juglandaceae sp.	12				0	0	0	0	0	[illegible]
SM.B_Me_3306	Dicot. sp. indet.	12				0	0	0	0	0	[illegible]
SM.B_Me_3317	Laurophyllum lanigeroides	3	15			0	0	1	0	0	[illegible]
SM.B_Me_3319	Dicot. sp. indet.	12				0	0	0	0	0	[illegible]
SM.B_Me_3326	Rhodomyrtophyllum sinuatum	3				0	0	1	0	0	[illegible]
SM.B_Me_3327	Ficus sp.	12				0	0	0	0	0	[illegible]
SM.B_Me_3330	Ternstroemites dentaus	189				0	0	0	0	0	[illegible]

SM.B_Me_3342	Juglandaceae Formenkreis 1	2					0	1	0	0	0	0	0	0	0	0	0	0	0	0	0	0	0	0	0	[illegible]
SM.B_Me_3346	Vitaceae sp.	200					0	0	0	0	0	0	0	0	0	0	0	0	0	0	0	0	0	0	0	[illegible]
SM.B_Me_3349	Dicot. sp. indet.	208					0	0	0	0	0	0	0	0	0	0	0	0	0	0	0	0	0	0	0	[illegible]
SM.B_Me_3356	Dicot. sp. indet.	3					0	0	1	0	0	0	0	0	0	0	0	0	0	0	0	0	0	0	0	[illegible]
SM.B_Me_3357	Juglandaceae Formenkreis 1	33					0	0	0	0	0	0	0	0	0	0	0	0	0	0	0	0	0	0	0	[illegible]
SM.B_Me_3359	Laurophyllum sp.	36					0	0	0	0	0	0	0	0	0	0	0	0	0	0	0	0	0	0	0	[illegible]
SM.B_Me_3362	Daphnogene crebrigranosa	14					0	0	0	0	0	0	0	0	0	0	0	0	0	1	0	0	0	0	0	[illegible]
SM.B_Me_3363	Laurophyllum sp.	14					0	0	0	0	0	0	0	0	0	0	0	0	0	1	0	0	0	0	0	[illegible]
SM.B_Me_3365	Dicot. sp. indet.	208					0	0	0	0	0	0	0	0	0	0	0	0	0	0	0	0	0	0	0	[illegible]
SM.B_Me_3372	Laurophyllum lanigeroides	15					0	0	0	0	0	0	0	0	0	0	0	0	0	0	1	0	0	0	0	[illegible]
SM.B_Me_3377	Laurophyllum lanigeroides	14					0	0	0	0	0	0	0	0	0	0	0	0	0	1	0	0	0	0	0	[illegible]
SM.B_Me_3379	Dicot. sp. indet.	61					0	0	0	0	0	0	0	0	0	0	0	0	0	0	0	0	0	0	0	[illegible]
[illegible]	Laurophyllum sp.	207					0	0	0	0	0	0	0	0	0	0	0	0	0	0	0	0	0	0	0	[illegible]
SM.B_Me_3383	Juglandaceae sp.	152					0	0	0	0	0	0	0	0	0	0	0	0	0	0	0	0	0	0	0	[illegible]
SM.B_Me_3388	Dicot. sp. indet.	14					0	0	0	0	0	0	0	0	0	0	0	0	0	1	0	0	0	0	0	[illegible]
SM.B_Me_339	Dicot. sp. indet.	2					0	1	0	0	0	0	0	0	0	0	0	0	0	0	0	0	0	0	0	[illegible]
SM.B_Me_3395	Laurophyllum lanigeroides	206	15				0	0	0	0	0	0	0	0	0	0	0	0	0	0	1	0	0	0	0	[illegible]
SM.B_Me_3396	Rhodomyrtophyllum sinuatum	12	15				0	0	0	0	0	0	0	0	0	0	0	1	0	0	1	0	0	0	0	[illegible]
SM.B_Me_3397	Juglandaceae sp.	12					0	0	0	0	0	0	0	0	0	0	0	1	0	0	0	0	0	0	0	[illegible]
SM.B_Me_3399	Juglandaceae sp.	145					0	0	0	0	0	0	0	0	0	0	0	0	0	0	0	0	0	0	0	[illegible]
SM.B_Me_3400	Daphnogene crebrigranosa	2					0	1	0	0	0	0	0	0	0	0	0	0	0	0	0	0	0	0	0	[illegible]
SM.B_Me_3401	Juglandaceae sp.	14					0	0	0	0	0	0	0	0	0	0	0	0	0	1	0	0	0	0	0	[illegible]
SM.B_Me_3406	Laurophyllum lanigeroides	206					0	0	0	0	0	0	0	0	0	0	0	0	0	0	0	0	0	0	0	[illegible]
SM.B_Me_3411	Laurophyllum lanigeroides	12					0	0	0	0	0	0	0	0	0	0	0	1	0	0	0	0	0	0	0	[illegible]
SM.B_Me_3412	Juglandaceae sp.	13					0	0	0	0	0	0	0	0	0	0	0	0	1	0	0	0	0	0	0	[illegible]
SM.B_Me_3415	Leguminosae sp. 5	147					0	0	0	0	0	0	0	0	0	0	0	0	0	0	0	0	0	0	0	[illegible]
SM.B_Me_3419	Dicot. sp. indet.	14	12				0	0	0	0	0	0	0	0	0	0	0	1	0	1	0	0	0	0	0	[illegible]
SM.B_Me_3422	Juglandaceae Formenkreis 1	190					0	0	0	0	0	0	0	0	0	0	0	0	0	0	0	0	0	0	0	[illegible]
SM.B_Me_3425	Leguminosae sp. 5	13					0	0	0	0	0	0	0	0	0	0	0	0	1	0	0	0	0	0	0	[illegible]
SM.B_Me_3427	Daphnogene crebrigranosa	12					0	0	0	0	0	0	0	0	0	0	0	1	0	0	0	0	0	0	0	[illegible]
SM.B_Me_3434	Juglandaceae sp.	12					0	0	0	0	0	0	0	0	0	0	0	1	0	0	0	0	0	0	0	[illegible]
SM.B_Me_344	Dicot. sp. indet.	216					0	0	0	0	0	0	0	0	0	0	0	0	0	0	0	0	0	0	0	[illegible]
SM.B_Me_3440	Juglandaceae Formenkreis 1	2	12				0	1	0	0	0	0	0	0	0	0	0	1	0	0	0	0	0	0	0	[illegible]
SM.B_Me_3447	Juglandaceae Formenkreis 1	205					0	0	0	0	0	0	0	0	0	0	0	0	0	0	0	0	0	0	0	[illegible]
SM.B_Me_3462	Dicot. sp. indet.	36					0	0	0	0	0	0	0	0	0	0	0	0	0	0	0	0	0	0	0	[illegible]
SM.B_Me_3463	Ficus sp.	169	3	2		1	1	1	1	0	0	0	0	0	0	0	0	0	0	0	0	0	0	0	0	[illegible]
[illegible]	[illegible]	[illegible]	[illegible]	[illegible]	[illegible]	[illegible]	[illegible]	[illegible]	[illegible]	[illegible]	[illegible]	[illegible]	[illegible]	[illegible]	[illegible]	[illegible]	[illegible]	[illegible]	[illegible]	[illegible]	[illegible]	[illegible]	[illegible]	[illegible]	[illegible]	[illegible]

Wappler et al. SOM File S2

Wappler_et al_SOM_File_S1

SM.B_Me_9526	Dicot. sp. indet.	*195*		0	0	0	0	0	0	0	0	0	0	0	0	0	0	0	0	0	0	0
SM.B_Me_9527	Dicot. sp. indet.	*14*		0	0	0	0	0	0	0	0	0	0	0	0	0	1	0	0	0	0	0
SM.B_Me_953	Dicot. sp. indet.	*12*		0	0	0	0	0	0	0	0	0	0	0	1	0	0	0	0	0	0	0
SM.B_Me_9531	Rhodomyrtophyllum sinuatum	*208*		0	0	0	0	0	0	0	0	0	0	0	0	0	0	0	0	0	0	0
SM.B_Me_9533	Rhodomyrtophyllum sinuatum	*5*		0	0	0	0	1	0	0	0	0	0	0	0	0	0	0	0	0	0	0
SM.B_Me_9534	Rhodomyrtophyllum sinuatum	*12*		0	0	0	0	0	0	0	0	0	0	0	1	0	0	0	0	0	0	0
SM.B_Me_9540	? Vitaceae / ? Menispermaceae / ? Acer	*208*		0	0	0	0	0	0	0	0	0	0	0	0	0	0	0	0	0	0	0
SM.B_Me_9548	Rhodomyrtophyllum sinuatum	*208*		0	0	0	0	0	0	0	0	0	0	0	0	0	0	0	0	0	0	0
SM.B_Me_955	Dicot. sp. indet.	*12*		0	0	0	0	0	0	0	0	0	0	0	1	0	0	0	0	0	0	0
SM.B_Me_9558	Leguminosae sp. 5	*147*		0	0	0	0	0	0	0	0	0	0	0	0	0	0	0	0	0	0	0
SM.B_Me_9561	Leguminosae sp. 5	*147*		0	0	0	0	0	0	0	0	0	0	0	0	0	0	0	0	0	0	0
SM.B_Me_9562	Lauraceae sp.	*3*		0	0	1	0	0	0	0	0	0	0	0	0	0	0	0	0	0	0	0
[illegible]	Dicot. sp. indet.	*106*		0	0	0	0	0	0	0	0	0	0	0	0	0	0	0	0	0	0	0
SM.B_Me_9579	Daphnogene sp.	*3*		0	0	1	0	0	0	0	0	0	0	0	0	0	0	0	0	0	0	0
SM.B_Me_9586	Lauraceae sp.	*12*		0	0	0	0	0	0	0	0	0	0	0	1	0	0	0	0	0	0	0
SM.B_Me_9595	Lauraceae sp.	*3*		0	0	1	0	0	0	0	0	0	0	0	0	0	0	0	0	0	0	0
SM.B_Me_9596	Lauraceae sp.	*3*	*12*	0	0	1	0	0	0	0	0	0	0	0	1	0	0	0	0	0	0	0
SM.B_Me_9597	Lauraceae sp.	*12*		0	0	0	0	0	0	0	0	0	0	0	1	0	0	0	0	0	0	0
SM.B_Me_9602	Daphnogene sp.	*13*		0	0	0	0	0	0	0	0	0	0	0	0	1	0	0	0	0	0	0
SM.B_Me_9605	Laurophyllum sp.	*12*		0	0	0	0	0	0	0	0	0	0	0	1	0	0	0	0	0	0	0
SM.B_Me_9610	Laurophyllum lanigeroides	*3*		0	0	1	0	0	0	0	0	0	0	0	0	0	0	0	0	0	0	0
SM.B_Me_9614	Lauraceae sp.	*145*		0	0	0	0	0	0	0	0	0	0	0	0	0	0	0	0	0	0	0
SM.B_Me_9623	Lauraceae sp.	*54*		0	0	0	0	0	0	0	0	0	0	0	0	0	0	0	0	0	0	0
SM.B_Me_9625	Leguminosae sp. 5	*14*		0	0	0	0	0	0	0	0	0	0	0	0	0	1	0	0	0	0	0
SM.B_Me_9630	Lauraceae sp.	*12*		0	0	0	0	0	0	0	0	0	0	0	1	0	0	0	0	0	0	0
SM.B_Me_9634	Dicot. sp. indet.	*12*		0	0	0	0	0	0	0	0	0	0	0	1	0	0	0	0	0	0	0
SM.B_Me_964	Juglandaceae Formenkreis 2	*15*		0	0	0	0	0	0	0	0	0	0	0	0	0	0	1	0	0	0	0
SM.B_Me_9640	Dicot. sp. GM004	*12*		0	0	0	0	0	0	0	0	0	0	0	1	0	0	0	0	0	0	0
SM.B_Me_9642	Myricaceae sp.	*195*	*152*	0	0	0	0	0	0	0	0	0	0	0	0	0	0	0	0	0	0	0
SM.B_Me_9645	Leguminosae sp. 5	*147*		0	0	0	0	0	0	0	0	0	0	0	0	0	0	0	0	0	0	0
SM.B_Me_9659	Rhodomyrtophyllum sinuatum	*12*		0	0	0	0	0	0	0	0	0	0	0	1	0	0	0	0	0	0	0
SM.B_Me_9663	Myricaceae sp.	*3*		0	0	1	0	0	0	0	0	0	0	0	0	0	0	0	0	0	0	0
SM.B_Me_9667	Juglandaceae sp.	*2*		0	1	0	0	0	0	0	0	0	0	0	0	0	0	0	0	0	0	0
SM.B_Me_9670	Juglandaceae sp.	*12*		0	0	0	0	0	0	0	0	0	0	0	1	0	0	0	0	0	0	0
SM.B_Me_9671	Juglandaceae sp.	*12*		0	0	0	0	0	0	0	0	0	0	0	1	0	0	0	0	0	0	0
SM.B_Me_9681	Rhodomyrtophyllum sinuatum	*200*		0	0	0	0	0	0	0	0	0	0	0	0	0	0	0	0	0	0	0
SM.B_Me_9682	Juglandaceae sp.	*12*		0	0	0	0	0	0	0	0	0	0	0	1	0	0	0	0	0	0	0
SM.B_Me_9684	Juglandaceae sp.	*206*		0	0	0	0	0	0	0	0	0	0	0	0	0	0	0	0	0	0	0
SM.B_Me_9694	Dicot. sp. indet.	*12*		0	0	0	0	0	0	0	0	0	0	0	1	0	0	0	0	0	0	0
SM.B_Me_9698	Leguminosae sp. 5	*147*		0	0	0	0	0	0	0	0	0	0	0	0	0	0	0	0	0	0	0
SM.B_Me_9713	Dicot. sp. indet.	*190*		0	0	0	0	0	0	0	0	0	0	0	0	0	0	0	0	0	0	0
SM.B_Me_9715	Laurophyllum sp.	*12*		0	0	0	0	0	0	0	0	0	0	0	1	0	0	0	0	0	0	0
SM.B_Me_9721	Dicot. sp. indet.	*80*		0	0	0	0	0	0	0	0	0	0	0	0	0	0	0	0	0	0	0
SM.B_Me_9722	Dicot. sp. indet.	*12*		0	0	0	0	0	0	0	0	0	0	0	1	0	0	0	0	0	0	0
SM.B_Me_9728	Juglandaceae sp.	*12*		0	0	0	0	0	0	0	0	0	0	0	1	0	0	0	0	0	0	0
SM.B_Me_973	Ficus sp.	*12*		0	0	0	0	0	0	0	0	0	0	0	1	0	0	0	0	0	0	0
SM.B_Me_9734	Dicot. sp. indet.	*208*		0	0	0	0	0	0	0	0	0	0	0	0	0	0	0	0	0	0	0
SM.B_Me_9739	Dicot. sp. indet.	*12*		0	0	0	0	0	0	0	0	0	0	0	1	0	0	0	0	0	0	0
SM.B_Me_9745	Dicot. sp. indet.	*12*		0	0	0	0	0	0	0	0	0	0	0	1	0	0	0	0	0	0	0
SM.B_Me_9748	Juglandaceae sp.	*12*		0	0	0	0	0	0	0	0	0	0	0	1	0	0	0	0	0	0	0
SM.B_Me_975	Ficus sp.	*14*		0	0	0	0	0	0	0	0	0	0	0	0	0	1	0	0	0	0	0
SM.B_Me_9759	Juglandaceae sp.	*14*		0	0	0	0	0	0	0	0	0	0	0	0	0	1	0	0	0	0	0
SM.B_Me_9762	Dicot. sp. indet.	*12*		0	0	0	0	0	0	0	0	0	0	0	1	0	0	0	0	0	0	0
SM.B_Me_977	Dicot. sp. indet.	*119*		0	0	0	0	0	0	0	0	0	0	0	0	0	0	0	0	0	0	0
SM.B_Me_9774	Dicot. sp. indet.	*13*		0	0	0	0	0	0	0	0	0	0	0	0	1	0	0	0	0	0	0
SM.B_Me_9775	Dicot. sp. indet.	*13*		0	0	0	0	0	0	0	0	0	0	0	0	1	0	0	0	0	0	0
SM.B_Me_9777	Dicot. sp. indet.	*3*		0	0	1	0	0	0	0	0	0	0	0	0	0	0	0	0	0	0	0
SM.B_Me_9779	Dicot. sp. indet.	*3*	*12*	0	0	1	0	0	0	0	0	0	0	0	1	0	0	0	0	0	0	0
SM.B_Me_978	Leguminosae sp. 5	*147*		0	0	0	0	0	0	0	0	0	0	0	0	0	0	0	0	0	0	0
SM.B_Me_9788	Dicot. sp. indet.	*78*		0	0	0	0	0	0	0	0	0	0	0	0	0	0	0	0	0	0	0
SM.B_Me_979	Juglandaceae Formenkreis 1	*14*		0	0	0	0	0	0	0	0	0	0	0	0	0	1	0	0	0	0	0
SM.B_Me_9794	Juglandaceae Formenkreis 1	*211*		0	0	0	0	0	0	0	0	0	0	0	0	0	0	0	0	0	0	0
SM.B_Me_980	Dicot. sp. indet.	*195*	*12*	0	0	0	0	0	0	0	0	0	0	0	1	0	0	0	0	0	0	0
SM.B_Me_982	Lauraceae sp.	*142*		0	0	0	0	0	0	0	0	0	0	0	0	0	0	0	0	0	0	0
SM.B_Me_9869	Dicot. sp. indet.	*12*		0	0	0	0	0	0	0	0	0	0	0	1	0	0	0	0	0	0	0
SM.B_Me_9875	Juglandaceae sp.	*3*	*12*	0	0	1	0	0	0	0	0	0	0	0	1	0	0	0	0	0	0	0
SM.B_Me_9880	Dicot. sp. indet.	*163*		0	0	0	0	0	0	0	0	0	0	0	0	0	0	0	0	0	0	0
SM.B_Me_9883	Dicot. sp. indet.	*216*		0	0	0	0	0	0	0	0	0	0	0	0	0	0	0	0	0	0	0
SM.B_Me_9886	Juglandaceae Formenkreis 4	*4*		0	0	0	1	0	0	0	0	0	0	0	0	0	0	0	0	0	0	0
SM.B_Me_9888	Laurophyllum sp.	*76*	*3*	0	0	1	0	0	0	0	0	0	0	0	0	0	0	0	0	0	0	0
SM.B_Me_9889	Juglandaceae sp.	*208*		0	0	0	0	0	0	0	0	0	0	0	0	0	0	0	0	0	0	0
SM.B_Me_9901	Lauraceae sp.	*145*		0	0	0	0	0	0	0	0	0	0	0	0	0	0	0	0	0	0	0
SM.B_Me_9903	Juglandaceae Formenkreis 4	*12*	*4*	0	0	0	1	0	0	0	0	0	0	0	1	0	0	0	0	0	0	0
SM.B_Me_9912	Lauraceae sp.	*36*		0	0	0	0	0	0	0	0	0	0	0	0	0	0	0	0	0	0	0
SM.B_Me_9915	Dicot. sp. indet.	*12*		0	0	0	0	0	0	0	0	0	0	0	1	0	0	0	0	0	0	0
SM.B_Me_9916	Dicot. sp. indet.	*12*		0	0	0	0	0	0	0	0	0	0	0	1	0	0	0	0	0	0	0
SM.B_Me_9927	Juglandaceae sp.	*12*		0	0	0	0	0	0	0	0	0	0	0	1	0	0	0	0	0	0	0
SM.B_Me_9928	Ulmaceae sp.	*3*		0	0	1	0	0	0	0	0	0	0	0	0	0	0	0	0	0	0	0
SM.B_Me_9932	Dicot. sp. indet.	*16*		0	0	0	0	0	0	0	0	0	0	0	0	0	0	0	1	0	0	0
SM.B_Me_9935	Laurophyllum sp.	*14*		0	0	0	0	0	0	0	0	0	0	0	0	0	1	0	0	0	0	0
SM.B_Me_9938	Juglandaceae Formenkreis 1	*76*		0	0	0	0	0	0	0	0	0	0	0	0	0	0	0	0	0	0	0
SM.B_Me_9945	Dicot. sp. GM001	*32*		0	0	0	0	0	0	0	0	0	0	0	0	0	0	0	0	0	0	0
SM.B_Me_9947	Lauraceae sp.	*12*		0	0	0	0	0	0	0	0	0	0	0	1	0	0	0	0	0	0	0
SM.B_Me_9951	Rhodomyrtophyllum sinuatum	*22*		0	0	0	0	0	0	0	0	0	0	0	0	0	0	0	0	0	0	0
SM.B_Me_9968	Dicot. sp. indet.	*195*		0	0	0	0	0	0	0	0	0	0	0	0	0	0	0	0	0	0	0
SM.B_Me_9969	Dicot. sp. indet.	*2*		0	1	0	0	0	0	0	0	0	0	0	0	0	0	0	0	0	0	0
SM.B_Me_9974	Laurophyllum lanigeroides	*16*		0	0	0	0	0	0	0	0	0	0	0	0	0	0	0	1	0	0	0
SM.B_Me_9976	Juglandaceae Formenkreis 2	*2*		0	1	0	0	0	0	0	0	0	0	0	0	0	0	0	0	0	0	0
SM.B_Me_9981	Juglandaceae sp.	*12*		0	0	0	0	0	0	0	0	0	0	0	1	0	0	0	0	0	0	0
SM.B_Me_9987	Laurophyllum sp.	*12*		0	0	0	0	0	0	0	0	0	0	0	1	0	0	0	0	0	0	0
SM.B_Me_9989	Dicot. sp. indet.	*12*		0	0	0	0	0	0	0	0	0	0	0	1	0	0	0	0	0	0	0
SM.B_Me_9990	Juglandaceae Formenkreis 1	*12*		0	0	0	0	0	0	0	0	0	0	0	1	0	0	0	0	0	0	0
SM.B_Me_9991	Dicot. sp. GM001	*32*		0	0	0	0	0	0	0	0	0	0	0	0	0	0	0	0	0	0	0
SM.B_Me_9993	Leguminosae sp. 5	*147*		0	0	0	0	0	0	0	0	0	0	0	0	0	0	0	0	0	0	0

DT 179	DT 180	DT 181	DT 182	DT 183	DT 184	DT 185	DT 186	DT 187	DT 188	DT 189	DT 190	DT 191	DT 192	DT 193	DT 194	DT 195	DT 196	DT 197	DT 198	DT 199	DT 200	DT 201	DT 202	DT 203	DT 204	DT 205	DT 206	DT 207	DT 208	DT 209	DT 210	DT 211	DT 212	DT 213	DT 214	DT 215	DT 216	DT 217	DT 218	DT 219	DT 220	DT 221	DT 222	DT 223
0	0	0	0	0	0	0	0	0	0	0	0	0	0	0	0	0	0	0	0	0	0	0	0	0	0	0	0	0	0	0	0	0	0	0	0	0	0	0	0	0	0	0	0	0
0	0	0	0	0	0	0	0	0	0	0	0	0	0	0	0	0	0	0	0	0	0	0	0	0	0	0	0	0	0	0	0	0	0	0	0	0	0	0	0	0	0	0	0	0
0	0	0	0	0	0	0	0	0	0	0	0	0	0	0	0	0	0	0	0	0	0	0	0	0	0	0	0	0	0	0	0	0	0	0	0	0	0	0	0	0	0	0	0	0
0	0	0	0	0	0	0	0	0	0	0	0	0	0	0	0	0	0	0	0	0	0	0	0	0	0	0	0	0	0	0	0	0	0	0	0	0	0	0	0	0	0	0	0	0
0	0	0	0	0	0	0	0	0	0	0	0	0	0	0	0	0	0	0	0	0	0	0	0	0	0	0	0	0	0	0	0	0	0	0	0	0	0	0	0	0	0	0	0	0
0	0	0	0	0	0	0	0	0	0	0	0	0	0	0	0	0	0	0	0	0	0	0	0	0	0	0	0	0	0	0	0	0	0	0	0	0	0	0	0	0	0	0	0	0
0	0	0	0	0	0	0	0	0	0	0	0	0	0	0	0	0	0	0	0	0	0	1	0	0	0	0	0	0	0	0	0	0	0	0	0	0	0	0	0	0	0	0	0	0
0	0	0	0	0	0	0	0	0	0	0	0	0	0	0	0	0	0	0	0	0	0	0	0	0	0	0	0	0	0	0	0	0	0	0	0	0	0	0	0	0	0	0	0	0
0	0	0	0	0	0	0	0	0	0	0	0	0	0	0	0	0	0	0	0	0	0	0	0	0	0	0	0	0	0	0	0	0	0	0	0	0	0	0	0	0	0	0	0	0
0	0	0	0	0	0	0	0	0	0	0	0	0	0	0	0	0	0	0	0	0	0	0	0	0	0	0	0	0	0	0	0	0	0	0	0	0	0	0	0	0	0	0	0	0
[illegible]	[illegible]	[illegible]	0	0	0	0	0	0	0	0	0	0	0	0	0	0	0	0	0	0	0	0	0	0	0	0	0	0	0	0	0	0	0	0	0	0	0	0	0	0	0	0	0	0
0	0	0	0	0	0	0	0	0	0	0	0	0	0	0	0	0	0	0	0	0	0	0	0	0	0	0	0	0	0	0	0	0	0	0	0	0	0	0	0	0	0	0	0	0
0	0	0	0	0	0	0	0	0	0	0	0	0	0	0	0	0	0	0	0	0	0	0	0	0	0	0	0	0	0	0	0	0	0	0	0	0	0	0	0	0	0	0	0	0
0	0	0	0	0	0	0	0	0	0	0	0	0	0	0	0	0	0	0	0	0	0	0	0	0	0	0	0	0	0	0	0	0	0	0	0	0	0	0	0	0	0	0	0	0
0	0	0	0	0	0	0	0	0	0	0	0	0	0	0	0	0	0	0	0	0	0	0	0	0	0	0	0	0	0	0	0	0	0	0	0	0	0	0	0	0	0	0	0	0
0	0	0	0	0	0	0	0	0	0	0	0	0	0	0	0	0	0	0	0	0	0	0	0	0	0	0	0	0	0	0	0	0	0	0	0	0	0	0	0	0	0	0	0	1
0	0	0	0	0	0	0	0	0	0	0	0	0	0	0	0	0	0	0	0	0	0	0	0	0	0	0	0	0	0	0	0	0	0	0	0	0	0	0	0	0	0	0	0	0
0	0	0	0	0	0	0	0	0	0	0	0	0	0	0	0	0	0	0	0	0	0	0	0	0	0	0	0	0	0	0	0	0	0	0	0	0	1	0	0	0	0	0	0	0
0	0	0	0	0	0	0	0	0	0	0	0	0	0	0	0	0	0	0	0	0	0	0	0	0	0	0	0	0	0	0	0	0	0	0	0	0	0	0	0	0	1	0	0	0
0	0	0	0	0	0	0	0	0	0	0	0	0	0	0	0	0	0	0	0	0	0	0	0	0	0	0	0	0	0	0	0	0	0	0	0	0	0	0	0	0	0	0	0	0
0	0	0	0	0	0	0	0	0	0	1	0	0	0	0	0	0	0	0	0	0	0	0	0	0	0	0	0	0	0	0	0	0	0	0	0	0	0	0	0	0	0	0	0	0
0	0	0	0	0	0	0	0	0	0	0	0	0	0	0	0	0	0	0	0	0	0	0	0	0	0	0	0	0	0	0	0	0	0	0	0	0	0	0	0	0	0	0	0	0
0	0	0	0	0	0	0	0	0	0	0	0	0	0	0	0	0	0	0	0	0	0	0	0	0	0	0	0	0	0	0	0	0	0	0	0	0	0	0	0	0	0	0	0	0
0	0	0	0	0	0	0	0	0	0	0	0	0	0	0	0	0	0	0	0	0	0	0	0	0	0	0	0	0	0	0	0	0	0	0	0	0	0	0	0	0	0	0	0	0
0	0	0	0	0	0	0	0	0	0	0	0	0	0	0	0	0	0	0	0	0	0	0	0	0	0	0	0	0	0	0	0	0	0	0	0	0	0	0	0	0	0	0	0	0
0	0	0	0	0	0	0	0	0	0	0	0	0	0	0	0	0	0	0	0	0	0	0	0	0	0	0	0	0	0	0	0	0	0	0	0	0	0	0	0	0	0	0	0	0
0	0	0	0	0	0	0	0	0	0	0	0	0	0	0	0	0	0	0	0	0	0	0	0	0	0	0	0	0	0	0	0	0	0	0	0	0	0	0	0	0	0	0	0	0
0	0	0	0	0	0	0	0	0	0	0	0	0	0	0	0	0	0	0	0	0	0	0	0	0	0	0	0	0	0	0	0	0	0	0	0	0	0	0	0	0	0	0	0	0
0	0	0	0	0	0	0	0	0	0	0	0	0	0	0	0	0	0	0	0	0	0	0	0	0	0	0	1	0	0	0	0	0	0	0	0	0	0	0	0	0	0	0	0	0
0	0	0	0	0	0	0	0	0	0	0	0	0	0	0	0	0	0	0	0	0	0	0	0	0	0	0	0	0	0	0	0	0	0	0	0	0	0	0	0	0	0	0	0	0
0	0	0	0	0	0	0	0	0	0	0	0	0	0	0	0	0	0	0	0	0	0	1	0	0	0	0	0	0	0	0	0	0	0	0	0	0	0	0	0	0	0	0	0	0
0	0	0	0	0	0	0	0	0	0	0	0	0	0	0	0	0	0	0	0	0	0	0	0	0	0	0	0	0	0	0	0	0	0	0	0	0	0	0	0	0	0	0	0	0
0	0	0	0	0	0	0	0	0	0	0	0	0	0	0	0	0	0	0	1	0	0	0	0	0	0	0	0	0	0	0	0	0	0	0	0	0	0	0	0	0	0	0	0	0
0	0	0	0	0	0	0	0	0	0	0	0	0	0	0	0	0	0	0	0	0	0	0	0	0	0	0	0	0	0	0	0	0	0	0	0	0	0	0	0	0	0	0	0	0
0	0	0	0	0	0	0	0	0	0	0	0	0	0	0	0	0	0	0	1	0	0	0	0	0	0	0	0	1	0	0	0	0	0	0	0	0	0	0	0	0	0	0	0	0
0	0	0	0	0	0	0	0	0	0	0	0	0	0	0	0	0	0	0	0	0	0	0	0	0	0	0	0	0	0	0	0	0	0	0	0	0	0	0	0	0	0	0	0	0
0	0	0	0	0	0	0	0	0	0	0	0	0	0	0	0	0	0	0	0	0	0	0	0	0	0	0	0	0	0	0	0	0	0	0	0	0	0	0	0	0	0	0	0	0
0	0	0	0	0	0	0	0	0	0	0	0	0	0	0	0	0	0	0	0	0	0	0	0	0	0	0	0	0	0	0	0	0	0	0	0	0	0	0	0	0	0	0	0	0
0	0	0	0	0	0	0	0	0	0	0	0	0	0	0	0	0	0	0	0	0	0	0	0	0	0	0	0	0	0	0	0	0	0	0	0	0	0	0	0	0	0	0	0	0
0	0	0	0	0	0	0	0	0	0	0	0	0	0	0	0	0	0	0	0	0	0	0	0	0	0	0	0	0	1	0	0	0	0	0	0	0	0	0	0	0	0	0	0	0
0	0	0	0	0	0	0	0	0	0	0	0	0	0	0	0	0	0	0	0	0	0	0	0	0	0	0	0	0	0	0	0	0	0	0	0	0	0	0	0	0	0	0	1	0
0	0	0	0	0	0	0	0	0	0	0	0	0	0	0	0	0	0	0	0	0	0	0	0	0	0	0	0	0	0	0	0	0	0	0	0	0	0	0	0	0	0	0	0	0
0	0	0	0	0	0	0	0	0	0	0	0	0	0	0	0	0	0	0	0	0	0	0	0	0	0	0	0	0	0	0	0	0	0	0	0	0	0	0	0	0	0	0	0	0
0	0	0	0	0	0	0	0	0	0	0	0	0	0	0	0	0	0	0	0	0	0	0	0	0	0	0	0	0	0	0	0	0	0	0	0	0	0	0	0	0	0	0	0	0
0	0	0	0	0	0	0	0	0	0	0	0	0	0	0	0	0	0	0	0	0	0	0	0	0	0	0	0	0	0	0	0	0	0	0	0	0	0	0	0	0	0	0	0	0
0	0	0	0	0	0	0	0	0	0	0	0	0	0	0	0	0	0	0	0	0	0	0	0	0	0	0	0	0	1	0	0	0	0	0	0	0	0	0	0	0	0	0	0	0
0	0	0	0	0	0	0	0	0	0	0	0	0	0	0	0	0	0	0	0	0	0	0	0	0	0	0	0	0	0	0	0	0	0	0	0	0	0	0	0	0	0	0	0	0
0	0	0	0	0	0	0	0	0	0	0	0	0	0	0	0	0	0	0	0	0	0	0	0	0	0	0	0	0	0	0	0	0	0	0	0	0	0	0	0	0	0	0	0	0
0	0	0	0	0	0	0	0	0	0	0	0	0	0	0	0	0	0	0	0	0	0	0	0	0	0	0	0	0	0	0	0	0	0	0	0	0	0	0	0	0	0	0	0	0
0	0	0	0	0	0	0	0	0	0	0	0	0	0	0	0	0	0	0	0	0	0	0	0	0	0	0	0	0	0	0	0	0	0	0	0	0	0	0	0	0	0	0	0	0
0	0	0	0	0	0	0	0	0	0	0	0	0	0	0	0	0	0	0	0	0	0	0	0	0	0	0	0	0	0	0	0	0	0	0	0	0	0	0	0	0	0	0	0	0
0	0	0	0	0	0	0	0	0	0	0	0	0	0	0	0	0	0	0	0	0	0	0	0	0	0	0	0	0	0	0	0	0	0	0	0	0	0	0	0	0	0	0	0	0
0	0	0	0	0	0	0	0	0	0	0	0	0	0	0	0	0	0	0	0	0	0	0	0	0	0	0	0	0	0	0	0	0	0	0	0	0	1	0	0	0	0	0	0	0
0	0	0	0	0	0	0	0	0	0	0	0	0	0	0	0	0	0	0	0	0	0	0	0	0	0	0	0	0	0	0	0	0	0	0	0	0	0	0	0	0	0	0	0	0
0	0	0	0	0	0	0	0	0	0	0	0	0	0	0	0	0	0	0	0	0	0	0	0	0	0	0	0	0	0	0	0	0	0	0	0	0	0	0	0	0	0	0	0	0
0	0	0	0	0	0	0	0	0	0	0	0	0	0	0	0	1	0	0	0	0	0	0	0	0	0	0	0	0	0	0	0	0	0	0	0	0	0	0	0	0	0	0	0	0
0	0	0	0	0	0	0	0	0	0	0	0	0	0	0	0	0	0	0	0	0	0	0	0	0	0	0	0	0	0	0	0	0	0	0	0	0	0	0	0	0	0	0	0	0
0	0	0	0	0	0	0	0	0	0	0	0	0	0	0	0	0	0	0	0	0	0	0	0	0	0	0	1	0	0	0	0	0	0	0	0	0	0	0	0	0	0	0	0	0
0	0	0	0	0	0	0	0	0	0	0	0	0	0	0	0	0	0	0	0	0	0	0	0	0	0	0	0	0	0	0	0	0	0	0	0	0	0	0	0	0	0	0	0	0
0	0	0	0	0	0	0	0	0	0	0	0	0	0	0	0	0	0	0	0	0	0	0	0	0	0	0	0	0	0	0	0	0	0	0	0	0	0	0	0	0	0	0	0	0
0	0	0	0	0	0	0	0	0	0	0	0	0	0	0	0	0	0	0	0	0	0	0	0	0	0	0	0	0	0	0	0	0	0	0	0	0	0	0	0	0	0	0	0	0
0	0	0	0	0	0	0	0	0	0	0	0	0	0	0	0	0	0	0	0	0	0	0	0	0	0	0	1	0	0	0	0	0	0	0	0	0	0	0	0	0	0	0	0	0
0	0	0	0	0	0	0	0	0	0	0	0	0	0	0	0	0	0	0	0	0	0	0	0	0	0	0	0	0	0	0	0	0	0	0	0	0	0	0	0	0	0	0	0	0
0	0	0	0	0	0	0	0	0	0	0	0	0	0	0	0	0	0	0	0	0	0	0	0	0	0	0	0	0	0	0	0	0	0	0	0	0	0	0	0	0	0	0	0	0
0	0	0	0	0	0	0	0	0	0	0	0	0	0	0	0	0	0	0	0	0	0	0	0	0	0	0	0	0	0	0	0	0	0	0	0	0	0	0	0	0	0	0	0	0
0	0	0	0	0	0	0	0	0	0	0	0	0	0	0	0	0	0	0	0	0	0	0	0	0	0	0	0	0	0	0	0	0	0	0	0	0	0	0	0	0	0	0	0	0
0	0	0	0	0	0	0	0	0	0	0	0	0	0	0	0	0	0	0	0	0	0	0	0	0	0	0	0	0	0	0	0	0	0	0	0	0	0	0	0	0	0	0	0	0
0	0	0	0	0	0	0	0	0	0	0	0	0	0	0	0	0	0	0	0	0	0	0	0	0	0	0	0	0	0	0	0	0	0	0	0	0	0	0	0	0	0	0	0	0
0	0	0	0	0	0	0	0	0	0	0	0	0	0	0	0	0	0	0	0	0	0	0	0	0	0	0	0	0	0	0	0	0	0	0	0	0	0	0	0	0	0	0	1	0
0	0	0	0	0	0	0	0	0	0	0	0	0	0	0	0	0	0	0	0	0	0	0	0	0	0	0	0	0	1	0	0	0	0	0	0	0	0	0	0	0	0	0	0	0
0	0	0	0	0	0	0	0	0	0	0	0	0	0	0	0	0	0	0	0	0	0	0	0	0	0	0	0	0	0	0	0	0	0	0	0	0	0	0	0	0	0	0	0	0
0	0	0	0	0	0	0	0	0	0	0	0	0	0	0	0	0	0	0	0	0	0	0	0	0	0	0	0	0	0	0	0	0	0	0	0	0	0	0	0	0	0	0	0	0
0	0	0	0	0	0	0	0	0	0	0	0	0	0	0	0	0	0	0	0	0	0	0	0	0	0	0	0	0	0	0	0	0	0	0	0	0	0	0	0	0	0	0	0	0
0	0	0	0	0	0	0	0	0	0	0	0	0	0	0	0	0	0	0	0	0	0	0	0	0	0	0	0	0	0	0	0	0	0	0	0	0	0	0	0	0	0	0	0	0
0	0	0	0	0	0	0	0	0	0	0	0	0	0	0	0	0	0	0	0	0	0	0	0	0	0	0	0	0	0	0	0	0	0	0	0	0	0	0	0	0	0	0	0	0
0	0	0	0	0	0	0	0	0	0	1	0	0	0	0	0	0	0	0	0	0	0	0	0	0	0	0	0	0	0	0	0	0	0	0	0	0	0	0	0	0	0	0	0	0
0	0	0	0	0	0	0	0	0	0	0	0	0	0	0	0	0	0	0	0	0	0	0	0	0	0	0	0	0	0	0	0	0	0	0	0	0	0	0	0	0	0	0	0	0
0	0	0	0	0	0	0	0	0	0	0	0	0	0	0	0	0	0	0	0	0	0	0	0	0	0	0	1	0	0	0	0	0	0	0	0	0	0	0	0	0	0	0	0	0
0	0	0	0	0	0	0	0	0	0	0	0	0	0	0	0	0	0	0	0	0	0	0	0	0	0	0	0	0	0	0	0	0	0	0	0	0	0	0	0	0	0	0	0	0
0	0	0	0	0	0	0	0	0	0	0	0	0	0	0	0	0	0	0	0	0	0	0	0	0	0	0	0	0	0	0	1	0	0	0	0	0	0	0	0	0	0	0	0	0
0	0	0	0	0	0	0	0	0	0	0	0	0	0	0	0	0	0	0	0	0	0	0	0	0	0	0	0	0	0	0	0	0	0	0	0	0	0	0	0	0	0	0	0	0
0	0	0	0	0	0	0	0	0	0	0	0	0	0	0	0	0	0	0	0	0	0	0	0	0	0	0	0	0	0	0	0	0	0	0	0	0	0	0	0	0	0	0	0	0
0	0	0	0	0	0	0	0	0	0	0	0	0	0	0	0	0	0	0	0	0	0	0	0	0	0	0	0	0	0	0	0	0	0	0	0	0	0	0	0	0	0	0	0	0
0	0	0	0	0	0	0	0	0	0	0	0	0	0	0	0	0	0	0	0	0	0	0	0	0	0	0	0	0	0	0	0	0	0	0	0	0	0	0	0	0	0	0	0	0
0	0	0	0	0	0	0	0	0	0	0	0	0	0	0	0	0	0	0	0	0	0	0	0	0	0	0	0	0	0	0	1	0	0	0	0	0	0	0	0	0	0	0	0	0
0	0	0	0	0	0	0	0	0	0	0	0	0	0	0	0	0	0	0	0	0	0	0	0	0	0	0	0	0	0	0	0	0	0	0	0	0	0	0	0	0	0	0	0	0
0	0	0	0	0	0	0	0	0	0	0	0	0	0	0	0	0	0	0	0	0	0	0	0	0	0	0	0	0	0	0	0	0	0	0	0	0	0	0	0	0	0	0	0	0
0	0	0	0	0	0	0	0	0	0	0	0	0	0	0	0	0	0	0	0	0	0	0	0	0	0	0	0	0	0	0	1	0	0	0	0	0	0	0	0	0	0	0	0	0
0	0	0	0	0	0	0	0	0	0	0	0	0	0	0	0	0	0	0	0	0	0	0	0	0	0	0	0	0	0	0	0	0	0	0	0	0	0	0	0	0	0	0	0	0
0	0	0	0	0	0	0	0	1	0	0	0	0	0	0	0	0	0	0	0	0	0	0	0	0	0	0	0	0	0	0	0	0	0	0	0	0	0	0	0	0	0	0	0	0
0	0	0	0	0	0	0	0	0	0	0	0	0	0	0	0	0	0	0	0	0	0	0	0	0	0	0	0	0	0	0	0	0	0	0	0	0	0	0	0	0	0	0	0	0
0	0	0	0	0	0	0	0	0	0	0	0	0	0	0	0	0	0	0	0	0	0	0	0	0	0	0	0	0	0	0	0	0	0	0	0	0	0	0	0	0	0	0	0	0
0	0	0	0	0	0	0	0	0	0	0	0	0	0	0	0	0	0	0	0	0	0	0	0	0	0	0	0	0	0	0	0	0	0	0	0	0	0	0	0	0	0	0	0	0
0	0	0	0	0	0	0	0	0	0	0	0	0	0	0	0	0	0	0	0	0	0	0	0	0	0	0	0	0	0	0	0	0	0	0	0	0	0	0	0	0	0	0	0	0
0	0	0	0	0	0	0	0	0	0	0	0	0	0	0	0	0	0	0	0	0	0	0	0	0	0	0	0	0	1	0	0	0	0	0	0	0	0	0	0	0	0	0	0	0
0	0	0	0	0	0	0	0	0	0	0	0	0	0	0	0	0	0	0	0	0	0	0	0	0	0	0	0	0	0	0	0	1	0	0	0	0	0	0	0	0	0	0	0	0
0	0	0	0	0	0	0	0	0	1	0	0	0	0	0	0	0	0	0	0	0	0	0	0	0	0	0	0	0	0	0	0	0	0	0	0	0	0	0	0	0	0	0	0	0
0	0	0	0	0	0	0	0	0	0	0	0	0	0	0	0	0	0	0	0	0	0	0	0	0	0	0	0	0	0	0	0	0	0	0	0	0	0	0	0	0	0	0	0	0
0	0	0	0	0	0	0	0	0	0	0	0	0	0	0	0	0	0	0	0	0	0	0	0	0	0	0	0	0	0	0	0	0	0	0	0	0	0	0	0	0	0	0	0	0
0	0	0	0	0	0	0	0	0	0	0	0	0	0	0	0	0	0	0	0	0	0	0	0	0	0	0	0	0	0	0	0	0	0	0	0	0	0	0	0	0	0	0	0	0
0	0	0	0	0	0	0	0	0	0	0	0	0	0	0	0	0	0	0	0	0	0	0	0	0	0	0	0	0	0	0	0	0	0	0	1	0	0	0	0	0	0	0	0	0
0	0	0	0	0	0	0	0	0	0	0	0	0	0	0	0	0	0	0	0	0	0	0	0	0	0	0	0	0	0	0	0	0	0	0	0	0	0	0	0	0	0	0	0	0
0	0	0	0	0	0	0	0	0	0	0	0	0	0	0	0	0	0	0	0	0	0	0	0	0	0	0	0	0	1	0	0	0	0	0	0	0	0	0	0	0	0	0	0	0
0	0	0	0	0	0	0	0	0	0	0	0	0	0	0	0	0	0	0	0	0	0	0	0	0	0	0	0	0	0	0	0	0	0	0	0	0	0	0	0	0	0	0	0	0
0	0	0	0	0	0	0	0	0	0	0	0	0	0	0	0	0	0	0	0	0	0	0	0	0	0	0	0	0	0	0	0	0	0	0	0	0	0	0	0	0	0	0	0	0
0	0	0	0	0	0	0	0	0	0	0	0	0	0	0	0	0	0	0	0	0	0	0	0	0	0	0	0	0	0	0	0	0	0	0	0	0	0	0	0	0	0	0	0	0
0	0	0	0	0	0	0	0	0	0	0	0	0	0	0	0	0	0	0	0	0	0	0	0	0	0	0	0	0	0	0	0	0	0	0	0	0	0	0	0	0	0	0	0	0
0	0	0	0	0	0	0	0	0	0	0	0	0	0	0	0	0	0	0	0	0	0	0	0	0	0	0	0	0	0	0	0	0	0	0	0	0	0	0	0	0	0	0	0	0
0	0	0	0	0	0	0	0	0	0	0	0	0	0	0	0	0	0	0	0	0	0	0	0	0	0	0	0	0	0	0	0	0	0	0	0	0	0	0	0	0	0	0	0	0
0	0	0	0	0	0	0	0	0	0	0	0	0	0	0	0	0	0	0	0	0	0	0	0	0	0	0	0	0	0	0	0	0	0	0	0	0	0	0	0	0	0	0	0	0
0	0	0	0	0	0	0	0	0	0	0	1	0	0	0	0	0	0	0	0	0	0	0	0	0	0	0	0	0	0	0	0	0	0	0	0	0	0	0	0	0	0	0	0	0
0	0	0	0	0	0	0	0	0	0	0	0	0	0	0	0	0	0	0	0	0	0	0	0	0	0	0	0	0	0	0	0	0	0	0	0	0	0	0	0	0	0	0	0	0
0	0	0	0	0	0	0	0	0	0	0	0	0	0	0	0	0	0	0	0	0	0	0	0	0	0	0	0	0	0	0	0	0	0	0	0	0	0	0	0	0	0	0	0	0
0	0	0	0	0	0	0	0	0	0	0	0	0	0	0	0	0	0	0	0	0	0	0	0	0	0	0	0	0	0	0	0	0	0	0	0	0	0	0	0	0	0	0	0	0
0	0	0	0	0	0	0	0	0	0	0	0	0	0	0	0	0	0	0	0	0	0	0	0	0	0	0	0	0	0	0	0	0	0	0	0	0	0	0	0	0	0	0	0	0
0	0	0	0	0	0	0	0	0	0	0	0	0	0	0	0	0	0	0	0	0	0	0	0	0	0	0	0	0	0	0	0	0	0	0	0	0	0	0	0	0	0	0	0	0
0	0	0	0	0	0	0	0	0	0	0	0	0	0	0	0	0	0	0	0	0	0	0	0	0	0	0	0	0	0	0	0	0	0	0	0	0	0	0	0	0	0	0	0	0
0	0	0	0	0	0	0	0	0	0	0	0	0	0	0	0	0	0	0	0	0	0	0	0	0	0	0	0	0	0	0	0	0	0	0	0	0	0	0	0	0	0	0	0	0
0	0	0	0	0	0	0	0	0	0	0	0	0	0	0	0	0	0	0	0	0	0	0	0	0	0	0	0	0	0	0	0	0	0	0	0	0	0	0	0	0	0	0	0	0
0	0	0	0	0	0	0	0	0	0	0	0	0	0	0	0	0	0	0	0	0	0	0	0	0	0	0	0	0	0	0	0	0	0	0	0	0	0	0	0	0	0	0	0	0
0	0	0	0	0	0	0	0	0	0	0	0	0	0	0	0	0	0	0	0	0	0	0	0	0	0	0	0	0	0	0	0	0	0	0	0	0	0	0	0	0	0	0	0	0
0	0	0	0	0	0	0	0	0	0	0	0	0	0	0	0	0	0	0	0	0	0	0	0	0	0	0	0	0	0	0	0	0	0	0	0	0	0	0	0	0	0	0	0	0
0	0	0	0	0	0	0	0	0	0	0	0	0	0	0	0	0	0	0	0	0	0	0	0	0	0	0	0	0	0	0	0	0	0	1	0	0	0	0	0	0	0	0	0	0
0	0	0	0	0	0	0	0	0	0	0	0	0	0	0	0	0	0	0	0	0	0	0	0	0	0	0	0	0	0	0	0	0	0	0	0	0	0	0	0	0	0	0	0	0
0	0	0	0	0	0	0	0	0	0	0	0	0	0	0	0	0	0	0	0	0	0	0	0	0	0	0	0	0	0	0	0	0	0	0	0	0	0	0	0	0	0	0	0	0
0	0	0	0	0	0	0	0	0	0	0	0	0	0	0	0	0	0	0	0	0	0	0	0	0	0	0	0	0	0	0	0	0	0	0	0	0	0	0	0	0	0	0	0	0
0	0	0	0	0	0	0	0	0	0	0	0	0	0	0	0	0	0	0	0	0	0	0	0	0	0	0	0	0	1	0	0	0	0	0	0	0	0	0	0	0	0	0	0	0
0	0	0	0	0	0	0	0	0	0	0	0	0	0	0	0	0	0	0	0	0	0	0	0	0	0	0	0	0	0	0	0	0	0	0	0	0	0	0	0	0	0	0	0	0
0	0	0	0	0	0	0	0	0	0	0	0	0	0	0	0	0	0	0	0	0	0	0	0	0	0	0	0	0	0	0	0	0	0	0	0	0	0	0	0	0	0	0	0	0
0	0	0	0	0	0	0	0	0	0	0	0	0	0	0	0	0	0	0	0	0	0	0	0	0	0	0	0	0	0	0	0	0	0	0	0	0	0	0	0	0	0	0	0	0
0	0	0	0	0	0	0	0	0	0	0	0	0	0	0	0	0	0	0	0	0	0	0	0	0	0	0	0	0	0	0	0	0	0	0	0	0	0	0	0	0	0	0	0	0
0	0	0	0	0	0	0	0	0	0	0	0	0	0	0	0	0	0	0	0	0	0	0	0	0	0	0	0	0	0	0	0	0	0	0	0	0	0	0	0	0	0	0	0	0
0	0	0	0	0	0	0	0	0	0	0	0	0	0	0	0	0	0	0	0	0	0	0	0	1	0	0	0	0	0	0	0	0	0	0	0	0	0	0	0	0	0	0	0	0
0	0	0	0	0	0	0	0	0	0	0	0	0	0	0	0	0	0	0	0	0	0	0	0	0	0	0	0	0	0	0	0	0	0	0	0	0	0	0	0	0	0	0	0	0
0	0	0	0	0	0	0	0	0	0	0	0	0	0	0	0	0	0	0	0	0	0	0	0	0	0	0	0	0	0	0	0	0	0	0	0	0	0	0	0	0	0	0	0	0
0	0	0	0	0	0	0	0	0	0	0	0	0	0	0	0	0	0	0	0	0	0	0	0	0	0	0	0	0	0	0	0	0	0	0	0	0	0	0	0	0	0	0	0	0
0	0	0	0	0	0	0	0	0	0	0	0	0	0	0	0	0	0	0	0	0	0	0	0	0	0	0	0	0	0	0	0	0	0	0	0	0	0	0	0	0	0	0	0	0
0	0	0	0	0	0	0	0	0	0	0	0	0	0	0	0	0	0	0	0	0	0	0	0	0	0	0	0	0	0	0	0	0	0	0	0	0	0	0	0	0	0	0	0	0
0	0	0	0	0	0	1	0	0	0	0	0	0	0	0	0	0	0	0	0	0	0	0	0	0	0	0	0	0	0	0	0	0	0	0	0	0	0	0	0	0	0	0	0	0
0	0	0	0	0	0	0	0	0	0	0	0	0	0	0	0	0	0	0	0	0	0	0	0	0	0	0	0	0	0	0	0	0	0	0	0	0	0	0	0	0	0	0	0	1
0	0	0	0	0	0	0	0	0	0	0	0	0	0	0	0	0	0	0	0	0	0	0	0	0	0	0	0	0	0	0	0	0	0	0	0	0	0	0	0	0	0	0	0	0
0	0	0	0	0	0	0	0	0	0	0	0	0	0	0	0	0	0	0	0	0	0	0	0	0	0	0	0	0	0	0	0	0	0	0	0	0	0	0	0	0	0	0	0	0
0	0	0	0	0	0	0	0	0	0	0	0	0	0	0	0	0	0	0	0	0	0	0	0	0	0	0	0	0	0	0	0	0	0	0	0	0	0	0	0	0	0	0	0	0
0	0	0	0	0	0	0	0	0	0	0	0	0	0	0	0	0	0	0	0	0	0	0	0	0	0	0	0	0	0	0	0	0	0	0	0	0	0	0	0	0	0	0	0	0
0	0	0	0	0	0	0	0	0	0	0	0	0	0	0	0	0	0	0	0	0	0	0	0	0	0	0	0	0	0	0	0	0	0	0	0	0	0	0	0	0	0	0	0	0
0	0	0	0	0	0	0	0	0	0	0	0	0	0	0	0	0	0	0	0	0	0	0	0	0	0	0	1	0	0	0	0	0	0	0	0	0	0	0	0	0	0	0	0	0
0	0	0	0	0	0	0	0	0	0	0	0	0	0	0	0	0	0	0	0	0	0	0	0	0	0	0	1	0	0	0	0	0	0	0	0	0	0	0	0	0	0	0	0	0
0	0	0	0	0	0	0	0	0	0	0	0	0	0	0	0	0	0	0	0	0	0	0	0	0	0	0	0	0	0	0	0	0	0	0	0	0	0	0	0	0	0	0	0	0
0	0	0	0	0	0	0	0	0	0	0	0	0	0	0	0	0	0	0	0	0	0	0	0	0	0	0	0	0	0	0	0	0	0	0	0	0	0	0	0	0	0	0	0	0
0	0	0	0	0	0	0	0	0	0	0	0	0	0	0	0	0	0	0	0	0	0	0	0	0	0	0	0	0	0	0	0	0	0	0	0	0	0	0	0	0	0	0	0	0
0	0	0	0	0	0	0	0	0	0	0	0	0	0	0	0	0	0	0	0	0	0	0	0	0	0	0	0	0	0	0	0	0	0	0	0	0	0	0	0	0	0	0	0	0
0	0	0	0	0	0	0	0	0	0	0	0	0	0	0	0	0	0	0	0	0	0	0	0	0	0	0	0	0	0	0	0	0	0	0	0	0	0	0	0	0	0	0	0	0
0	0	0	0	0	0	0	0	0	0	0	0	0	0	0	0	0	0	0	0	0	0	0	0	0	0	0	0	0	0	0	0	0	0	0	0	0	1	0	0	0	0	0	0	0
0	0	0	0	0	0	0	0	0	0	0	0	0	0	0	0	0	0	0	0	0	0	0	0	0	0	0	0	0	0	0	0	0	0	0	0	0	1	0	0	0	0	0	0	0
0	0	0	0	0	0	0	0	0	0	0	0	0	0	0	0	0	0	0	0	0	0	0	0	0	0	0	0	0	0	0	0	0	0	0	0	0	0	0	0	0	0	0	0	0
0	0	0	0	0	0	0	0	0	0	0	0	0	0	0	0	0	0	0	0	0	0	0	0	0	0	0	0	0	0	0	0	0	0	0	0	0	0	0	0	0	0	0	0	0
0	0	0	0	0	0	0	0	0	0	0	0	0	0	0	0	0	0	0	0	0	0	0	0	0	0	0	0	0	0	0	0	0	0	0	0	0	0	0	0	0	0	0	0	0
0	0	0	0	0	0	0	0	0	0	0	0	0	0	0	0	0	0	0	0	0	0	0	0	0	0	0	0	0	0	0	0	0	0	0	0	0	0	0	0	0	0	0	0	0
0	0	0	0	0	0	0	0	0	0	0	0	0	0	0	0	0	0	0	0	0	0	0	0	0	0	0	0	0	0	0	0	0	0	0	0	0	0	0	0	0	0	0	0	0
0	0	0	0	0	0	0	0	0	0	0	0	0	0	0	0	0	0	0	0	0	0	0	0	0	0	0	0	0	0	0	0	0	0	0	0	0	1	0	0	0	0	0	0	0
0	0	0	0	0	0	0	0	0	0	0	0	0	0	0	0	0	0	0	0	0	0	0	0	0	0	0	0	0	0	0	1	0	0	0	0	0	0	0	0	0	0	0	0	0
0	0	0	0	0	0	0	0	0	0	0	0	0	0	0	0	0	0	0	0	0	0	0	0	0	0	0	0	0	0	0	0	0	0	0	0	0	0	0	0	0	0	0	0	0
0	0	0	0	0	0	0	0	0	0	0	0	0	0	0	0	0	0	0	0	0	0	0	0	0	0	0	0	0	1	0	0	0	0	0	0	0	0	0	0	0	0	0	0	0
0	0	0	0	0	0	0	0	0	0	0	0	0	0	0	0	0	0	0	0	0	0	0	0	0	0	0	0	0	0	0	0	0	0	0	0	0	0	0	0	0	0	0	0	0
0	0	0	0	0	0	0	0	0	0	0	0	0	0	0	0	0	0	0	0	0	0	0	0	0	0	0	0	0	0	0	0	0	0	0	0	0	0	0	0	0	0	0	0	0
0	0	0	0	0	0	0	0	0	0	0	0	0	0	0	0	0	0	0	0	0	0	0	0	0	0	0	0	0	0	0	0	0	0	0	0	0	0	0	0	0	0	0	0	0
0	0	0	0	0	0	0	0	0	0	0	0	0	0	0	0	0	0	0	0	0	0	0	0	0	0	0	0	0	0	0	0	0	0	0	0	0	0	0	0	0	0	0	0	0
0	0	0	0	0	0	0	0	0	0	0	0	0	0	0	0	0	0	0	0	0	0	0	0	0	0	0	0	0	0	0	0	0	0	0	0	0	0	0	0	0	0	0	0	0
0	0	0	0	0	0	0	0	0	0	0	0	0	0	0	0	0	0	0	0	0	0	0	0	0	0	0	0	0	0	0	0	0	0	0	0	0	0	0	0	0	1	0	0	0
0	0	0	0	0	0	0	0	0	0	0	0	0	0	0	0	0	0	0	0	0	0	0	0	0	0	0	0	0	0	0	0	0	0	0	0	0	0	0	0	0	0	0	0	0
0	0	0	0	0	0	0	0	0	0	0	0	0	0	0	0	0	0	0	0	0	0	0	0	0	0	0	0	0	0	0	0	0	0	0	0	0	0	0	0	0	0	0	0	0
0	0	0	0	0	0	0	0	0	0	0	0	0	0	0	0	0	0	0	0	0	0	0	0	0	0	0	1	0	0	0	0	0	0	0	0	0	0	0	0	0	0	0	0	0
0	0	0	0	0	0	0	0	0	0	0	0	0	0	0	0	0	0	0	0	0	0	0	0	0	0	0	0	0	0	0	0	0	0	0	0	0	0	0	0	0	0	0	0	0
0	0	0	0	0	0	0	0	0	0	0	0	0	0	0	0	0	0	0	0	0	0	0	0	0	0	0	0	0	0	0	0	0	0	0	0	0	1	0	0	0	0	0	0	0
0	0	0	0	0	0	0	0	0	0	0	0	0	0	0	0	0	0	0	0	0	0	0	0	0	0	0	0	0	0	0	0	0	0	0	0	0	0	0	0	0	0	0	0	0
0	0	0	0	0	0	0	0	0	0	0	0	0	0	0	0	0	0	0	0	0	0	0	0	0	0	0	0	0	0	0	0	0	0	0	0	0	0	0	0	0	0	0	0	0
0	0	0	0	0	0	0	0	0	0	0	0	0	0	0	0	0	0	0	0	0	0	0	0	0	0	0	0	0	1	0	0	0	0	0	0	0	0	0	0	0	0	0	0	0
0	0	0	0	0	0	0	0	0	0	0	0	0	0	0	0	0	0	0	0	0	0	0	0	0	0	0	0	0	0	0	0	0	0	0	0	0	0	0	0	0	0	0	0	0
0	0	0	0	0	0	0	0	0	0	0	0	0	0	0	0	0	0	0	0	0	0	0	0	0	0	0	0	0	0	0	0	0	0	0	0	0	0	0	0	0	0	0	0	0
0	0	0	0	0	0	0	0	0	0	0	0	0	0	0	0	0	0	0	0	0	0	0	0	0	0	0	0	0	0	0	0	0	0	0	0	0	0	0	0	0	0	0	0	0
0	0	0	0	0	0	0	0	0	0	0	0	0	0	0	0	0	0	0	0	0	0	0	0	0	0	0	0	0	0	0	0	0	0	0	0	0	0	0	0	0	0	0	0	0
0	0	0	0	0	0	0	0	0	0	0	0	0	0	0	0	0	0	0	0	0	0	0	0	0	0	0	0	0	0	0	0	0	0	0	0	0	0	0	0	0	0	0	0	0
0	0	0	0	0	0	0	0	0	0	0	0	0	0	0	0	0	0	0	0	0	0	0	0	0	0	0	0	0	1	0	0	0	0	0	0	0	0	0	0	0	0	0	0	0
0	0	0	0	0	0	0	0	0	0	0	0	0	0	0	0	0	0	0	0	0	0	0	0	0	0	0	0	0	0	0	0	0	0	0	0	0	0	0	0	0	0	0	0	0
0	0	0	0	0	0	0	0	0	0	0	0	0	0	0	0	0	0	0	0	0	0	0	0	0	0	0	0	0	1	0	0	0	0	0	0	0	0	0	0	0	0	0	0	0
0	0	0	0	0	0	0	0	0	0	0	0	0	0	0	0	0	0	0	0	0	0	0	0	0	0	0	0	0	1	0	0	0	0	0	0	0	0	0	0	0	0	0	0	0
0	0	0	0	0	0	0	0	0	0	0	0	0	0	0	0	0	0	0	0	0	0	0	0	0	0	0	0	0	0	0	0	0	0	0	0	0	0	0	0	0	0	0	0	0
0	0	0	0	0	0	0	0	0	0	0	0	0	0	0	0	0	0	0	0	0	0	0	0	0	0	0	0	0	0	0	0	0	0	0	0	0	0	0	0	0	0	0	0	0
0	0	0	0	0	0	0	0	0	0	0	0	0	0	0	0	1	0	0	0	0	0	0	0	0	0	0	0	0	0	0	0	0	0	0	0	0	0	0	0	0	0	0	0	0
0	0	0	0	0	0	0	0	0	0	0	0	0	0	0	0	0	0	0	0	0	0	0	0	0	0	0	0	0	0	0	0	0	0	0	0	0	1	0	0	0	0	0	0	0
0	0	0	0	0	0	0	0	0	0	0	0	0	0	0	0	0	0	0	0	0	0	0	0	0	0	0	0	0	0	0	0	0	0	0	0	0	0	0	0	0	0	0	0	0
0	0	0	0	0	0	0	0	0	0	0	0	0	0	0	0	0	0	0	0	0	0	0	0	0	0	0	0	0	0	0	0	0	0	0	0	0	0	0	0	0	0	0	0	0
0	0	0	0	0	0	0	0	0	0	0	0	0	0	0	0	0	0	0	0	0	0	0	0	0	0	0	0	0	0	0	0	0	0	0	0	0	0	0	0	0	0	0	0	0
0	0	0	0	0	0	0	0	0	0	0	0	0	0	0	0	0	0	0	0	0	0	0	0	0	0	0	0	0	0	0	0	0	0	0	0	0	0	0	0	0	0	0	0	0
0	0	0	0	0	0	0	0	0	0	0	0	0	0	0	0	1	0	0	0	0	0	0	0	0	0	0	0	0	0	0	0	1	0	0	0	0	0	0	0	0	0	0	0	0
0	0	0	0	0	0	0	0	0	0	0	0	0	0	0	0	0	0	0	0	0	0	0	0	0	0	0	0	0	0	0	0	0	0	0	0	0	0	0	0	0	0	0	0	0
0	0	0	0	0	0	0	0	0	0	0	0	0	0	0	0	0	0	0	0	0	0	0	0	0	0	0	0	0	0	0	0	0	0	0	0	0	0	0	0	0	0	0	0	0
0	0	0	0	0	0	0	0	0	0	0	0	0	0	0	0	0	0	0	0	0	0	0	0	0	0	0	0	0	0	0	0	0	0	0	0	0	0	0	0	0	0	0	0	0
0	0	0	0	0	0	0	0	0	0	0	0	0	0	0	0	0	0	0	0	0	0	0	0	0	0	0	0	0	0	0	0	0	0	0	0	0	0	0	0	0	0	0	0	0
0	0	0	0	0	0	0	0	0	0	0	0	0	0	0	0	0	0	0	0	0	0	0	0	0	0	0	0	0	0	0	0	0	0	0	0	0	0	0	0	0	0	0	0	0
0	0	0	0	0	0	0	0	0	0	0	0	0	0	0	0	0	0	0	0	0	0	0	0	0	0	0	0	0	0	0	0	0	0	0	0	0	0	0	0	0	0	0	0	0
0	0	0	0	0	0	0	0	0	0	0	0	0	0	0	0	0	0	0	0	0	0	0	0	0	0	0	0	0	0	0	0	0	0	0	0	0	0	0	0	0	0	0	0	0
0	0	0	0	0	0	0	0	0	0	0	0	0	0	0	0	1	0	0	0	0	0	0	0	0	0	0	0	0	0	0	0	0	0	0	0	0	0	0	0	0	0	0	0	0
0	0	0	0	0	0	0	0	0	0	0	0	0	0	0	0	0	0	0	0	0	0	0	0	0	0	0	0	0	0	0	0	0	0	0	0	0	0	0	0	0	0	0	0	0
0	0	0	0	0	0	0	0	0	0	0	0	0	0	0	0	0	0	0	0	0	0	0	0	0	0	0	0	0	0	0	0	0	0	0	0	0	0	0	0	0	0	0	0	0
0	0	0	0	0	0	0	0	0	0	0	0	0	0	0	0	0	0	0	0	0	0	0	0	0	0	0	0	0	0	0	0	0	1	0	0	0	0	0	0	0	0	0	0	0
0	0	0	0	0	0	0	0	0	0	0	0	0	0	0	0	0	0	0	0	0	0	0	0	0	0	0	0	0	1	0	0	0	0	0	0	0	0	0	0	0	0	0	0	0
0	0	0	0	0	0	0	0	0	0	0	0	0	0	0	0	0	0	0	0	0	0	0	0	0	0	0	0	0	1	0	0	0	0	0	0	0	0	0	0	0	0	0	0	0
0	0	0	0	0	0	0	0	0	0	0	0	0	0	0	0	0	0	0	0	0	0	0	0	0	0	0	0	0	0	0	0	0	0	0	0	0	0	0	0	0	0	0	0	0
0	0	0	0	0	0	0	0	0	0	0	0	0	0	0	0	0	0	0	0	0	0	0	0	0	0	0	0	0	0	0	0	0	0	0	0	0	0	0	0	0	0	0	0	0
0	0	0	0	0	0	0	0	0	0	0	0	0	0	0	0	0	0	0	0	0	0	0	0	0	0	0	0	0	1	0	0	0	0	0	0	0	0	0	0	0	0	0	0	0
0	0	0	0	0	0	0	0	0	0	0	0	0	0	0	0	0	0	0	0	0	0	0	0	0	0	0	0	0	0	0	0	0	0	0	0	0	0	0	0	0	0	0	0	0
0	0	0	0	0	0	0	0	0	0	0	0	0	0	0	0	0	0	0	0	0	0	0	0	0	0	0	0	0	0	0	0	0	0	0	0	0	0	0	0	0	0	0	0	0
0	0	0	0	0	0	0	0	0	0	0	0	0	0	0	0	0	0	0	0	0	0	0	0	0	0	0	0	0	0	0	0	0	0	0	0	0	0	0	0	0	0	0	0	0
0	0	0	0	0	0	0	0	0	0	0	0	0	0	0	0	0	0	0	0	0	0	0	0	0	0	0	0	0	0	0	0	0	0	0	0	0	0	0	0	0	0	0	0	0
0	0	0	0	0	0	0	0	0	0	0	0	0	0	0	0	0	0	0	0	0	0	0	0	0	0	0	0	0	0	0	0	0	0	0	0	0	0	0	0	0	0	0	0	0
0	0	0	0	0	0	0	0	0	0	0	0	0	0	0	0	0	0	0	0	0	0	0	0	0	0	0	0	0	1	0	0	0	0	0	0	0	0	0	0	0	0	0	0	0
0	0	0	0	0	0	0	0	0	0	0	0	0	0	0	0	0	0	0	0	0	0	0	0	0	0	0	0	0	0	0	0	0	0	0	0	0	0	0	0	0	0	0	0	0
0	0	0	0	0	0	0	0	0	0	0	0	0	0	0	0	0	0	0	0	0	0	0	0	0	0	0	0	0	0	0	0	0	0	0	0	0	0	0	0	0	0	0	0	0
0	0	0	0	0	0	0	0	0	0	0	0	0	0	0	0	0	0	0	0	0	0	0	0	0	0	0	0	0	0	0	0	0	0	0	0	0	0	0	0	0	0	0	0	0
0	0	0	0	0	0	0	0	0	0	0	0	0	0	0	0	0	0	0	0	0	0	0	0	0	0	0	0	0	0	1	0	0	0	0	0	0	0	0	0	0	0	0	0	0
0	0	0	0	0	0	0	0	0	0	0	0	0	0	0	0	0	0	0	0	0	0	0	0	0	0	0	0	0	0	0	0	0	0	0	0	0	0	0	0	0	0	0	0	0
0	0	0	0	0	0	0	0	0	0	0	0	0	0	0	0	0	0	0	0	0	0	0	0	0	0	0	0	0	0	0	0	0	0	0	0	0	0	0	0	0	0	0	0	0
0	0	0	0	0	0	0	0	0	0	0	0	0	0	0	0	0	0	0	0	0	0	0	0	0	0	0	0	0	0	0	0	0	0	0	0	0	0	0	0	0	0	0	0	0
0	0	0	0	0	0	0	0	0	0	0	0	0	0	0	0	0	0	0	0	0	0	0	0	0	0	0	0	0	0	0	0	0	0	0	0	0	0	0	0	0	0	0	0	0
0	0	0	0	0	0	0	0	0	0	0	0	0	0	0	0	0	0	0	0	0	0	0	0	0	0	0	0	0	1	0	0	0	0	0	0	0	0	0	0	0	0	0	0	0
0	0	0	0	0	0	0	0	0	0	0	0	0	0	0	0	0	0	0	0	0	0	0	0	0	0	0	0	0	0	0	0	0	0	0	0	0	0	0	0	0	0	0	0	0
0	0	0	0	0	0	0	0	0	0	0	0	0	0	0	0	0	0	0	0	0	0	0	0	0	0	0	0	0	0	0	0	0	0	0	0	0	0	0	0	0	0	0	0	0
0	0	0	0	0	0	0	0	0	0	0	0	0	0	0	0	0	0	0	0	0	0	0	0	0	0	0	0	0	1	0	0	0	0	0	0	0	0	0	0	0	0	0	0	0
0	0	0	0	0	0	0	0	0	0	0	0	0	0	0	0	0	0	0	0	0	0	0	0	0	0	0	0	0	0	0	0	0	0	0	0	0	0	0	0	0	0	0	0	0
0	0	0	0	0	0	1	0	0	0	0	0	0	0	0	0	0	0	0	0	0	0	0	0	0	0	0	0	0	0	0	0	0	0	0	0	0	0	0	0	0	0	0	0	0
0	0	0	0	0	0	0	0	0	0	0	0	0	0	0	0	0	0	0	0	0	0	0	0	0	0	0	0	0	0	0	0	0	0	0	0	0	0	0	0	0	0	0	0	0
0	0	0	0	0	0	0	0	0	0	0	0	0	0	0	0	0	0	0	0	0	0	0	0	0	0	0	0	0	0	0	0	0	0	0	0	0	0	0	0	0	0	0	0	0
0	0	0	0	0	0	0	0	0	0	0	0	0	0	0	0	0	0	0	0	0	0	0	0	0	0	0	0	0	0	0	0	0	0	0	0	0	0	0	0	0	0	0	0	0
0	0	0	0	0	0	0	0	0	0	0	0	0	0	0	0	0	0	0	0	0	0	0	0	0	0	0	0	0	0	0	0	0	0	0	0	0	0	0	0	0	0	0	0	0
0	0	0	0	0	0	0	0	0	0	0	0	0	0	0	0	0	0	0	0	0	0	0	0	0	0	0	0	0	0	0	0	0	0	0	0	0	0	0	0	0	0	0	0	0
0	0	0	0	0	0	0	0	0	0	0	0	0	0	0	0	0	0	0	0	0	0	0	0	0	0	0	0	0	1	0	0	0	0	0	0	0	0	0	0	0	0	0	0	0
0	0	0	0	0	0	0	0	0	0	0	0	0	0	0	0	0	0	0	0	0	0	0	0	0	0	0	0	0	0	0	0	0	0	0	0	0	0	0	0	0	0	0	0	0
0	0	0	0	0	0	0	0	0	0	0	0	0	0	0	0	0	0	0	0	0	0	0	0	0	0	0	0	0	0	0	0	0	0	0	0	0	0	0	0	0	0	0	0	0
0	0	0	0	0	0	0	0	0	0	0	0	0	0	0	0	0	0	0	0	0	0	0	0	0	0	0	0	0	0	0	0	0	0	0	0	0	0	0	0	0	0	0	0	0
0	0	0	0	0	0	0	0	0	0	0	0	0	0	0	0	0	0	0	0	0	0	0	0	0	0	0	0	0	0	0	0	0	0	0	0	0	0	0	0	0	0	0	0	0
0	0	0	0	0	0	0	0	0	0	0	0	0	0	0	0	0	0	0	0	0	0	0	0	0	0	0	0	0	0	0	0	0	0	0	0	0	0	0	0	0	0	0	0	0
0	0	0	0	0	0	0	0	0	0	0	0	0	0	0	0	0	0	0	0	0	0	0	0	0	0	0	0	0	0	0	0	0	0	0	0	0	0	0	0	0	0	0	0	0
0	0	0	0	0	0	0	0	0	0	0	0	0	0	0	0	0	0	0	0	0	0	0	0	0	0	0	0	0	0	0	0	0	0	0	0	0	0	0	0	0	0	0	0	0
0	0	0	0	0	0	0	0	0	0	0	0	0	0	0	0	0	0	0	0	0	0	0	0	0	0	0	0	0	0	0	0	0	0	0	0	0	0	0	0	0	0	0	0	0
0	0	0	0	0	0	0	0	0	0	0	0	0	0	0	0	0	0	0	0	0	0	0	0	0	0	0	0	0	0	0	0	0	0	0	0	0	0	0	0	0	0	0	0	0
0	0	0	0	0	0	0	0	0	0	1	0	0	0	0	0	0	0	0	0	0	0	0	0	0	0	0	0	0	0	0	0	0	0	0	0	0	0	0	0	0	0	0	0	0
0	0	0	0	0	0	0	0	0	0	0	0	0	0	0	0	0	0	0	0	0	0	0	0	0	0	0	0	0	0	0	0	0	0	0	0	0	0	0	0	0	0	0	0	0
0	0	0	0	0	0	0	0	0	0	0	0	0	0	0	0	0	0	0	0	0	0	0	0	0	0	0	0	0	0	0	0	0	0	0	0	0	0	0	0	0	0	0	0	0
0	0	0	0	0	0	0	0	0	0	0	0	0	0	0	0	0	0	0	0	0	0	0	0	0	0	0	0	0	0	0	0	0	0	0	0	0	0	0	0	0	0	0	0	0
0	0	0	0	0	0	0	0	0	0	0	0	0	0	0	0	0	0	0	0	0	0	0	0	0	0	0	0	0	0	0	0	0	0	0	0	0	0	0	0	0	0	0	0	1
0	0	0	0	0	0	0	0	0	0	0	0	0	0	0	0	0	0	0	0	0	0	0	0	0	0	0	0	0	0	0	0	0	0	0	0	0	0	0	0	0	0	0	0	0
0	0	0	0	0	0	0	0	0	0	0	0	0	0	0	0	0	0	0	0	0	0	0	0	0	0	0	0	0	0	0	0	0	0	0	0	0	0	0	0	0	0	0	0	0
0	0	0	0	0	0	0	0	0	0	0	0	0	0	0	0	0	0	0	0	0	0	0	0	0	0	0	0	0	0	0	0	0	0	0	0	0	0	0	0	0	0	0	0	0
0	0	0	0	0	0	0	0	0	0	0	0	0	0	0	0	0	0	0	0	0	0	0	0	0	0	0	0	0	0	0	0	0	0	0	0	0	0	0	0	0	0	0	0	0
0	0	0	0	0	0	0	0	0	0	0	0	0	0	0	0	0	0	0	0	0	0	0	0	0	0	0	0	0	0	0	0	0	0	0	0	0	0	0	0	0	0	0	0	0
0	0	0	0	0	0	0	0	0	0	0	0	0	0	0	0	0	0	0	0	0	0	0	0	0	0	0	0	0	0	0	0	0	0	0	0	0	0	0	0	0	0	0	0	0
0	0	0	0	0	0	0	0	0	0	0	0	0	0	0	0	0	0	0	0	0	0	0	0	0	0	0	0	0	0	0	0	0	0	0	0	0	0	0	0	0	0	0	0	0
0	0	0	0	0	0	0	0	0	0	0	0	0	0	0	0	0	0	0	0	0	0	0	0	0	0	0	0	0	0	0	0	0	0	0	0	0	0	0	0	0	0	0	0	0
0	0	0	0	0	0	0	0	0	0	1	0	0	0	0	0	0	0	0	0	0	0	0	0	0	0	0	0	0	0	0	0	0	0	0	0	0	0	0	0	0	0	0	0	0
0	0	0	0	0	0	0	0	0	0	0	0	0	0	0	0	0	0	0	0	0	0	0	0	0	0	0	0	0	0	0	0	0	0	0	0	0	0	0	0	0	0	0	0	0
0	0	0	0	0	0	0	0	0	0	0	0	0	0	0	0	0	0	0	0	0	0	0	0	0	0	0	0	0	0	0	1	0	0	0	0	0	0	0	0	0	0	0	0	0
0	0	0	0	0	0	0	0	0	0	0	0	0	0	0	0	0	0	0	0	0	0	0	0	0	0	0	0	0	0	0	0	0	0	0	0	0	0	0	0	0	0	0	0	0
0	0	0	0	0	0	0	0	0	0	0	0	0	0	0	0	0	0	0	0	0	0	0	0	0	0	0	0	0	0	0	0	0	0	0	0	0	0	0	0	0	0	0	0	0
0	0	0	0	0	0	0	0	0	0	0	0	0	0	0	0	0	0	0	0	0	0	0	0	0	0	0	0	0	0	0	0	0	0	0	0	0	0	0	0	0	0	0	0	0
0	0	0	0	0	0	0	0	0	0	0	0	0	0	0	0	0	0	0	0	0	0	0	0	0	0	0	0	0	0	0	0	0	0	0	0	0	0	0	0	0	0	0	0	0
0	0	0	0	0	0	0	0	0	0	0	0	0	0	0	0	0	0	0	0	0	0	0	0	0	0	0	0	0	0	0	0	0	0	0	0	0	0	0	0	0	0	0	0	0
0	0	0	0	0	0	0	0	0	0	0	0	0	0	0	0	0	0	0	0	0	0	0	0	0	0	0	0	0	0	0	0	0	0	0	0	0	0	0	0	0	0	0	0	0
0	0	0	0	0	0	0	0	0	0	0	0	0	0	0	0	0	0	0	0	0	0	0	0	0	0	0	0	0	0	0	0	0	0	0	0	0	0	0	0	0	0	0	0	0
0	0	0	0	0	0	0	0	0	0	0	0	0	0	0	0	0	0	0	0	0	0	0	0	0	0	0	0	0	0	0	0	0	0	0	0	0	0	0	0	0	0	0	0	0
0	0	0	0	0	0	0	0	0	0	0	0	0	0	0	0	0	0	0	0	0	0	0	0	0	0	0	0	0	0	0	0	0	0	0	0	0	0	0	0	0	0	0	0	0
0	0	0	0	0	0	0	0	0	0	0	0	0	0	0	0	0	0	0	0	0	0	0	0	0	0	0	0	0	0	0	0	0	0	0	0	0	0	0	0	0	0	0	0	0
0	0	0	0	0	0	0	0	0	0	0	0	0	0	0	0	0	0	0	0	0	0	0	0	0	0	0	0	0	0	0	0	0	0	0	0	0	0	0	0	0	0	0	0	0
0	0	0	0	0	0	0	0	0	0	0	0	0	0	0	0	0	0	0	0	0	0	0	0	0	0	0	0	0	0	0	0	0	0	0	0	0	0	0	0	0	0	0	0	0
0	0	0	0	0	0	0	0	0	0	0	0	0	0	0	0	0	0	0	0	0	0	0	0	0	0	0	0	0	0	0	0	0	0	0	0	0	0	0	0	0	0	0	0	0
0	0	0	0	0	0	0	0	0	0	0	0	0	0	0	0	0	0	0	0	0	0	0	0	0	0	0	0	0	0	0	0	0	0	0	0	0	0	0	0	0	0	0	0	0
0	0	0	0	0	0	0	0	0	0	0	0	0	0	0	0	0	0	0	0	0	0	0	0	0	0	0	0	0	0	0	0	0	0	0	0	0	0	0	0	0	0	0	0	0
0	0	0	0	0	0	0	0	0	0	0	0	0	0	0	0	0	0	0	0	0	0	0	0	0	0	0	0	0	0	0	0	0	0	0	0	0	0	0	0	0	0	0	0	0
0	0	0	0	0	0	0	0	0	0	0	0	0	0	0	0	0	0	0	0	0	0	0	0	0	0	0	0	0	0	0	0	0	0	0	0	0	0	0	0	0	0	0	0	0
0	0	0	0	0	0	0	0	0	0	0	0	0	0	0	0	0	0	0	0	0	0	0	0	0	0	0	0	0	0	0	0	0	0	0	0	0	0	0	0	0	0	0	0	0
0	0	0	0	0	0	0	0	0	0	0	0	0	0	0	0	0	0	0	0	0	1	0	0	0	0	0	0	0	0	0	0	0	0	0	0	0	0	0	0	0	0	0	0	0
0	0	0	0	0	0	0	0	0	0	0	0	0	0	0	0	0	0	0	0	0	0	0	0	0	0	0	0	0	1	0	0	0	0	0	0	0	0	0	0	0	0	0	0	0
0	0	0	0	0	0	0	0	0	0	0	1	0	0	0	0	0	0	0	0	0	0	0	0	0	0	0	0	0	0	0	0	0	0	0	0	0	0	0	0	0	0	0	0	0
0	0	0	0	0	0	0	0	0	0	0	0	0	0	0	0	0	0	0	0	0	0	0	0	0	0	0	0	0	1	0	0	0	0	0	0	0	0	0	0	0	0	0	0	0
0	0	0	0	0	0	0	0	0	0	0	0	0	0	0	0	0	0	0	0	0	0	0	0	0	0	0	0	0	0	0	0	0	0	0	0	0	0	0	0	0	0	0	0	0
0	0	0	0	0	0	0	0	0	0	0	0	0	0	0	0	0	0	0	0	0	0	0	0	0	0	0	0	0	0	0	0	0	0	0	0	0	0	0	0	0	0	0	0	0
0	0	0	0	0	0	0	0	0	0	0	0	0	0	0	0	0	0	0	0	0	0	0	0	0	0	0	0	0	0	0	0	0	0	0	0	0	0	0	0	0	0	0	0	0
0	0	0	0	0	0	0	0	0	0	0	0	0	0	0	0	0	0	0	0	0	0	0	0	0	0	0	0	0	0	0	0	0	0	0	0	0	0	0	0	0	0	0	0	0
0	0	0	0	0	0	0	0	0	0	0	0	0	0	0	0	0	0	0	0	0	0	0	0	0	0	0	0	0	0	0	0	0	0	0	0	0	0	0	0	0	0	0	0	0
0	0	0	0	0	0	0	0	0	0	0	0	0	0	0	0	0	0	0	0	0	0	0	0	0	0	0	0	0	0	0	0	0	0	0	0	0	0	0	0	0	0	0	0	0
0	0	0	0	0	0	0	0	0	0	0	0	0	0	0	0	0	0	0	0	0	0	0	0	0	0	0	0	0	0	0	0	0	0	0	0	0	0	0	0	0	0	0	0	0
0	0	0	0	0	0	0	0	0	0	0	0	0	0	0	0	0	0	0	0	0	0	0	0	0	0	0	0	0	0	0	0	0	0	0	0	0	0	0	0	0	0	0	0	0
0	0	0	0	0	0	0	0	0	0	0	0	0	0	0	0	0	0	0	0	0	0	0	0	0	0	0	0	0	0	0	0	0	0	0	0	0	0	0	0	0	0	0	0	0
0	0	0	0	0	0	0	0	0	0	0	0	0	0	0	0	0	0	0	0	0	0	0	0	0	0	0	0	0	0	0	0	0	0	0	0	0	0	0	0	0	0	0	0	0
0	0	0	0	0	0	0	0	0	0	0	0	0	0	0	0	0	0	0	0	0	0	0	0	0	0	0	0	0	0	0	0	0	0	0	0	0	0	0	0	0	0	0	0	0
0	0	0	0	0	0	0	0	0	0	0	0	0	0	0	0	0	0	0	0	0	0	0	0	0	0	0	0	0	0	0	0	0	0	0	0	0	0	0	0	0	0	0	0	0
0	0	0	0	0	0	0	0	0	0	0	0	0	0	0	0	0	0	0	0	0	0	0	0	0	0	0	0	0	0	0	0	0	0	0	0	0	0	0	0	0	0	0	0	0
0	0	0	0	0	0	0	0	0	0	0	0	0	0	0	0	1	0	0	0	0	0	0	0	0	0	0	0	0	0	0	0	0	0	0	0	0	0	0	0	0	0	0	0	0
0	0	0	0	0	0	0	0	0	0	0	0	0	0	0	0	0	0	0	0	0	0	0	0	0	0	0	0	0	0	0	0	0	0	0	0	0	0	0	0	0	0	0	0	0
0	0	0	0	0	0	0	0	0	0	0	0	0	0	0	0	0	0	0	0	0	0	0	0	0	0	0	0	0	0	0	0	0	0	0	0	0	0	0	0	0	0	0	0	0
0	0	0	0	0	0	0	0	0	0	0	0	0	0	0	0	0	0	0	0	0	0	0	0	0	0	0	0	0	0	0	0	0	0	0	0	0	0	0	0	0	0	0	0	0
0	0	0	0	0	0	0	0	0	0	0	0	0	0	0	0	0	0	0	0	0	0	0	0	0	0	0	0	0	0	0	0	0	0	0	0	0	0	0	0	0	0	0	0	0
0	0	0	0	0	0	0	0	0	0	0	0	0	0	0	0	0	0	0	0	0	0	0	0	0	0	0	0	0	0	0	0	0	0	0	0	0	0	0	0	0	0	0	0	0
0	0	0	0	0	0	0	0	0	0	0	0	0	0	0	0	0	0	0	0	0	0	0	0	0	0	0	0	0	0	0	0	0	0	0	0	0	0	0	0	0	0	0	0	0
0	0	0	0	0	0	0	0	0	0	0	0	0	0	0	0	0	0	0	0	0	0	0	0	0	0	0	0	0	0	0	0	0	0	0	0	0	0	0	0	0	0	0	0	0
0	0	0	0	0	0	0	0	0	0	0	0	0	0	0	0	0	0	0	0	0	0	0	0	0	0	0	0	0	0	0	0	0	0	0	0	0	0	0	0	0	0	0	0	0
0	0	0	0	0	0	0	0	1	0	0	0	0	0	0	0	0	0	0	0	0	0	0	0	0	0	0	0	0	1	0	0	0	0	0	0	0	0	0	0	0	0	0	0	0
0	0	0	0	0	0	0	0	0	0	0	0	0	0	0	0	0	0	0	0	0	0	0	0	0	0	0	0	0	0	0	0	0	0	0	0	0	0	0	0	0	0	0	0	0
0	0	0	0	0	0	0	0	0	0	0	0	0	0	0	0	0	0	0	0	0	0	0	0	0	0	0	0	0	0	0	0	0	0	0	0	0	0	0	0	0	0	0	0	0
0	0	0	0	0	0	0	0	0	0	0	0	0	0	0	0	0	0	0	0	0	0	0	0	0	0	0	0	0	0	0	0	0	0	0	0	0	0	0	0	0	0	0	0	0
0	0	0	0	0	0	0	0	0	0	0	0	0	0	0	0	0	0	0	0	0	0	0	0	0	0	0	0	0	0	0	0	0	0	0	0	0	0	0	0	0	0	0	0	0
0	0	0	0	0	0	0	0	0	0	0	0	0	0	0	0	0	0	0	0	0	0	0	0	0	0	0	0	0	0	0	0	0	0	0	0	0	0	0	0	0	0	0	0	0
0	0	0	0	0	0	0	0	0	0	0	0	0	0	0	0	0	0	0	0	0	0	0	0	0	0	0	0	0	0	0	0	0	0	0	0	0	0	0	0	0	0	0	0	0
0	0	0	0	0	0	0	0	0	0	0	0	0	0	0	0	0	0	0	0	0	0	0	0	0	0	0	0	0	0	0	0	0	0	0	0	0	0	0	0	0	0	0	0	0
0	0	0	0	0	0	0	0	0	0	0	0	0	0	0	0	0	0	0	0	0	0	0	0	0	0	0	0	0	0	0	0	0	0	0	0	0	0	0	0	0	0	0	0	0
0	0	0	0	0	0	0	0	0	0	0	0	0	0	0	0	0	0	0	0	0	0	0	0	0	0	0	0	0	0	0	0	0	0	0	0	0	0	0	0	0	0	0	0	0
0	0	0	0	0	0	0	0	0	0	0	0	0	0	0	0	1	0	0	0	0	0	0	0	0	0	0	0	0	0	0	0	0	0	0	0	0	0	0	0	0	0	0	0	0
0	0	0	0	0	0	0	0	0	0	0	0	0	0	0	0	0	0	0	0	0	0	0	0	0	0	0	0	0	0	0	0	0	0	0	0	0	0	0	0	0	0	0	0	0
0	0	0	0	0	0	0	0	0	0	0	0	0	0	0	0	0	0	0	0	0	0	1	0	0	0	0	0	0	0	0	0	0	0	0	0	0	0	0	0	0	0	0	0	0
0	0	0	0	0	0	0	0	0	0	0	0	0	0	0	0	0	0	0	0	0	0	0	0	0	0	0	0	0	0	0	0	0	0	0	0	0	0	0	0	0	0	0	0	0
0	0	0	0	0	0	0	0	0	0	0	0	0	0	0	0	0	0	0	0	0	0	0	0	0	0	0	0	0	0	0	0	0	0	0	0	0	0	0	0	0	0	0	0	0
0	0	0	0	0	0	0	0	0	0	0	0	0	0	0	0	0	0	0	0	0	0	0	0	0	0	0	0	0	0	0	0	0	0	0	0	0	0	0	0	0	0	0	0	0
0	0	0	0	0	0	0	0	1	0	0	0	0	0	0	0	0	0	0	0	0	0	0	0	0	0	0	0	0	0	0	0	0	0	0	0	0	0	0	0	0	0	0	0	0
0	0	0	0	0	0	0	0	0	0	0	0	0	0	0	0	0	0	0	0	0	0	0	0	0	0	0	0	0	0	0	0	0	0	0	0	0	0	0	0	0	0	0	0	0
0	0	0	0	0	0	0	0	0	1	0	0	0	0	0	0	0	0	0	0	0	0	0	0	0	0	0	0	0	0	0	0	0	0	0	0	0	0	0	0	0	0	0	0	0
0	0	0	0	0	0	0	0	1	0	0	0	0	0	0	0	0	0	0	0	0	0	0	0	0	0	0	0	0	0	0	0	0	0	0	0	0	0	0	0	0	0	0	0	0
0	0	0	0	0	0	0	0	0	0	0	0	0	0	0	0	1	0	0	0	0	0	0	0	0	0	0	0	0	0	0	0	0	0	0	0	0	0	0	0	0	0	0	0	0
0	0	0	0	0	0	0	0	0	0	0	0	0	0	0	0	0	0	0	0	0	0	0	0	0	0	0	0	0	0	0	0	0	0	0	0	0	0	0	0	0	0	0	0	0
0	0	0	0	0	0	0	0	0	0	0	0	0	0	0	0	0	0	0	0	0	0	0	0	0	0	0	0	0	0	0	0	0	0	0	0	0	0	0	0	0	0	0	0	0
0	0	0	0	0	0	0	0	0	0	0	0	0	0	0	0	0	0	0	0	0	0	0	0	0	0	0	0	0	0	0	0	0	0	0	0	0	0	0	0	0	0	0	0	0
0	0	0	0	0	0	0	0	0	0	0	0	0	0	0	0	0	0	0	0	0	0	0	0	0	0	0	0	0	0	0	0	0	0	0	0	0	0	0	0	0	0	0	0	0
0	0	0	0	0	0	0	0	0	0	0	0	0	0	0	0	0	0	0	0	0	0	0	0	0	0	0	0	0	0	0	0	0	0	0	0	0	0	0	0	0	0	0	0	0
0	0	0	0	0	0	0	0	0	0	0	0	0	0	0	0	0	0	0	0	0	0	0	0	0	0	0	0	0	0	0	0	0	0	0	0	0	0	0	0	0	0	0	0	0
0	0	0	0	0	0	0	0	0	0	0	0	0	0	0	0	0	0	0	0	0	0	0	0	0	0	0	0	0	0	0	0	0	0	0	0	0	0	0	0	0	0	0	0	0
0	0	0	0	0	0	0	0	0	0	0	1	0	0	0	0	0	0	0	0	0	0	0	0	0	0	0	0	0	0	0	0	0	0	0	0	0	0	0	0	0	0	0	0	0
0	0	0	0	0	0	0	0	0	0	0	0	0	0	0	0	0	0	0	0	0	0	0	0	0	0	0	0	0	0	0	0	0	0	0	0	0	0	0	0	0	0	0	0	0
0	0	0	0	0	0	0	0	0	0	0	0	0	0	0	0	0	0	0	0	0	0	0	0	0	0	0	0	0	0	0	0	0	0	0	0	0	0	0	0	0	0	0	0	0
0	0	0	0	0	0	0	0	0	0	0	0	0	0	0	0	0	0	0	0	0	0	0	0	0	0	0	0	0	0	0	0	0	0	0	0	0	0	0	0	0	0	0	0	0
0	0	0	0	0	0	0	0	0	0	0	0	0	0	0	0	0	0	0	0	0	0	0	0	0	0	0	0	0	0	0	0	0	0	0	0	0	0	0	0	0	0	0	0	0
0	0	0	0	0	0	0	0	0	0	0	0	0	0	0	0	0	0	0	0	0	0	0	0	0	0	0	0	0	0	0	0	0	0	0	0	0	0	0	0	0	0	0	0	0
0	0	0	0	0	0	0	0	0	0	0	0	0	0	0	0	0	0	0	0	0	0	0	0	0	0	0	0	0	0	0	0	0	0	0	0	0	0	0	0	0	0	0	0	0
0	0	0	0	0	0	0	0	0	0	0	0	0	0	0	0	0	0	0	0	0	0	0	0	0	0	0	0	0	0	0	0	0	0	0	0	0	0	0	0	0	0	0	0	0
0	0	0	0	0	0	0	0	0	0	0	0	0	0	0	0	0	0	0	0	0	0	0	0	0	0	0	0	0	0	0	0	0	0	0	0	0	0	0	0	0	0	0	0	0
0	0	0	0	0	0	0	0	0	0	0	0	0	0	0	0	0	0	0	0	0	0	0	0	0	0	0	0	0	0	0	0	0	0	0	0	0	0	0	0	0	0	0	0	0
0	0	0	0	0	0	0	0	0	0	0	0	0	0	0	0	0	0	0	0	0	0	0	0	0	0	0	0	0	0	0	0	0	0	0	0	0	0	0	0	0	0	0	0	0
0	0	0	0	0	0	0	0	0	0	0	0	0	0	0	0	1	0	0	0	0	0	0	0	0	0	0	0	0	0	0	0	0	0	0	0	0	0	0	0	0	0	0	0	0
0	0	0	0	0	0	0	0	0	0	0	0	0	0	0	0	0	0	0	0	0	0	0	0	0	0	0	0	0	0	0	0	0	0	0	0	0	0	0	0	0	0	0	0	0
0	0	0	0	0	0	0	0	0	0	0	0	0	0	0	0	0	0	0	0	0	0	0	0	0	0	0	0	0	0	0	0	0	0	0	0	0	0	0	0	0	0	0	0	0
0	0	0	0	0	0	0	0	0	0	0	0	0	0	0	0	0	0	0	0	0	0	0	0	0	0	0	0	0	0	0	0	0	0	0	0	0	0	0	0	0	0	0	0	0
0	0	0	0	0	0	0	0	0	0	0	0	0	0	0	0	0	0	0	0	0	0	0	0	0	0	0	0	0	0	0	0	0	0	0	0	0	0	0	0	0	0	0	0	0
0	0	0	0	0	0	0	0	0	0	0	0	0	0	0	0	0	0	0	0	0	0	0	0	0	0	0	0	0	0	0	0	0	0	0	0	0	0	0	0	0	0	0	0	0
0	0	0	0	0	0	0	0	1	0	0	0	0	0	0	0	0	0	0	0	0	0	0	0	0	0	0	0	0	0	0	0	0	0	0	0	0	0	0	0	0	0	0	0	0
0	0	0	0	0	0	0	0	0	0	0	0	0	0	0	0	0	0	0	0	0	0	0	0	0	0	0	0	0	0	0	0	0	0	0	0	0	0	0	0	0	0	0	0	0
0	0	0	0	0	0	0	0	0	0	0	0	0	0	0	0	0	0	0	0	0	0	0	0	0	0	0	0	0	0	0	0	0	0	0	0	0	0	0	0	0	0	0	0	0
0	0	0	0	0	0	0	0	0	0	0	0	0	0	0	0	0	0	0	0	0	0	0	0	0	0	0	0	0	0	0	0	0	0	0	0	0	0	0	0	0	0	0	0	0
0	0	0	0	0	0	0	0	0	0	0	0	0	0	0	0	0	0	0	0	0	0	0	0	0	0	0	0	0	0	0	0	0	0	0	0	0	0	0	0	0	0	0	0	0
0	0	0	0	0	0	0	0	0	0	0	0	0	0	0	0	0	0	0	0	0	0	0	0	0	0	0	0	0	0	0	0	0	0	0	0	0	0	0	0	0	0	0	0	0
0	0	0	0	0	0	0	0	0	0	0	0	0	0	0	0	0	0	0	0	0	0	0	0	0	0	0	0	0	0	0	0	0	0	0	0	0	0	0	0	0	0	0	0	0
0	0	0	0	0	0	0	0	0	0	0	0	0	0	0	0	0	0	0	0	0	0	0	0	0	0	0	0	0	0	0	0	0	0	0	0	0	0	0	0	0	0	0	0	0
0	0	0	0	0	0	0	0	0	0	0	0	0	0	0	0	0	0	0	0	0	0	0	0	0	0	0	0	0	0	0	0	0	0	0	0	0	0	0	0	0	0	0	0	0
0	0	0	0	0	0	0	0	0	0	0	0	0	0	0	0	0	0	0	0	0	0	0	0	0	0	0	0	0	0	0	0	0	0	0	0	0	0	0	0	0	0	0	0	0
0	0	0	0	0	0	0	0	0	1	0	0	0	0	0	0	0	0	0	0	0	0	0	0	0	0	0	0	0	0	0	0	0	0	0	0	0	0	0	0	0	0	0	0	0
0	0	0	0	0	0	0	0	0	0	0	0	0	0	0	0	0	0	0	0	0	0	0	0	0	0	0	0	0	0	0	0	0	0	0	0	0	0	0	0	0	0	0	0	0

Wappler_et_al_SOM_File_S1

Waggler_et.al_SOM_File_S1

[illegible]

Waggler_et al_SOM File_S1

Waggler_et_al_SOM_File_S1

Waggler_et al_SOM_File_S1

Waggler_et al_SOM_File_S1

[illegible]
