# Supplementary material for: Testing for the Effects and Consequences of Mid Paleogene Climate Change on Insect Herbivory
Source: PLoS One. 2012 Jul 18;7(7):e40744. doi: 10.1371/journal.pone.0040744 (PMC3399891; doi:10.1371/journal.pone.0040744)
Supplement: File S2 — Complete Eckfeld insect damage dataset. (PDF) [file pone.0040744.s002.pdf]

Testing for the Effects and Consequences of Mid Paleogene Climate Change on Insect Herbivory

Torsten Wappler[1*], Conrad C. Labandeira[2,3], Jes Rust[1], Herbert Frankenhäuser[4], Volker Wilde[5]

1 Steinmann Institute, University of Bonn, 53115 Bonn, Germany; **2** Department of Paleobiology, National Museum of Natural History, Smithsonian Institution, Washington, DC 20013, USA; **3** Department of Entomology and BEES Program, University of Maryland, College Park, MD 20742, USA; **4** Mainz Natural History Museum / State Collection for Natural History of Rhineland-Palatine, Mainz, Germany; **5** Senckenberg Forschungsinstitut und Naturmuseum, Paläobotanik, Frankfurt am Main, Germany.

***** Author for correspondence.

ELECTRONIC SUPPLEMENTARY MATERIAL

FILE S2

Dataset 2. Summary data for the Eckfeld maar locality. The number of occurrences of each damage type (DT) was recorded.

Summary data for the [illegible] maar locality.

Collection number	Leaf species or morphotype	Damage type summary	DT 1	DT 2	DT 3	DT 4
PB_1989_24	Dicot. sp. indet.	12	0	0	0	0
PB_1989_67	Dicot. sp. indet.	189	0	0	0	0
PB_1989_68	Pungiphyllum waltheri	8	0	0	0	0
PB_1989_8	Juglandaceae sp.	2	0	1	0	0
PB_1990_160	Dicot. sp. indet.	206	0	0	0	0
PB_1990_165	Juglandaceae sp.	14	0	0	0	0
PB_1990_172	Dicot. sp., salicoid	12	0	0	0	0
PB_1990_181	Dicot. sp. indet.	11	0	0	0	0
PB_1990_187	Juglandaceae sp.	17	0	0	0	0
PB_1990_194	cf. Ternstroemites dentatus	12	0	0	0	0
[illegible]	Juglandaceae sp.	5 189	0	0	0	0
PB_1990_236	Dicot. sp. indet.	190	0	0	0	0
PB_1990_269	Dicot. sp. indet.	14 186	0	0	0	0
PB_1990_290	Juglandaceae sp.	2 206	0	1	0	0
PB_1990_296	Dicot. sp. indet.	12 32	0	0	0	0
PB_1990_59	Juglandaceae sp.	12	0	0	0	0
PB_1990_6	Dicot. sp. indet.	144	0	0	0	0
PB_1990_62	Juglandaceae sp.	15 176	0	0	0	0
PB_1990_66	Juglandaceae sp.	2	0	1	0	0
PB_1990_67	Dicot. sp. indet.	189	0	0	0	0
PB_1990_68	Dicot. sp., salicoid	14 15 41	0	0	0	0
PB_1990_69	Dicot. sp. indet.	12 14 189	0	0	0	0
PB_1990_77	Juglandaceae sp.	41	0	0	0	0
PB_1991_90	"Apocynophyllum sp."	12	0	0	0	0
PB_1991_93	Juglandaceae sp.	12	0	0	0	0
PB_1993_33	cf. Ternstroemites dentatus	12	0	0	0	0
PB_1995_171	Juglandaceae sp.	12	0	0	0	0
PB_1995_183	Dicot. sp., salicoid	8	0	0	0	0
PB_1995_184	Juglandaceae sp.	12	0	0	0	0
PB_1995_188	Juglandaceae sp.	12	0	0	0	0
PB_1995_191	Ulmaceae sp.	143	0	0	0	0
PB_1995_201	Lauraceae sp.	12	0	0	0	0
PB_1995_204	Lauraceae sp.	12	0	0	0	0
PB_1995_214	cf. Ternstroemites dentatus	2	0	1	0	0
PB_1995_216	cf. Ternstroemites dentatus	1	1	0	0	0
PB_1995_226	cf. Ternstroemites dentatus	1	1	0	0	0
PB_1995_239	cf. Ternstroemites dentatus	14	0	0	0	0
PB_1995_30	Pungiphyllum waltheri	12	0	0	0	0
PB_1995_44	Pungiphyllum waltheri	44	0	0	0	0
PB_1995_45	Pungiphyllum waltheri	33	0	0	0	0
PB_1995_57	Dicot. sp., carpinoid	12	0	0	0	0
PB_1995_73	Pungiphyllum waltheri	17	0	0	0	0
PB_1996 136	Dicot. sp., carpinoid	2	0	1	0	0
PB_1996 37	Dicot. sp. indet.	3	0	0	1	0
PB_1996 40	Dicot. sp. indet.	3	0	0	1	0
PB_1996 43	Dicot. sp. indet.	12	0	0	0	0
PB_1996 46	Dicot. sp. indet.	2	0	1	0	0
PB_1996 49	Dicot. sp. indet.	2 15	0	1	0	0
PB_1996 61	Dicot. sp. indet.	53	0	0	0	0
PB_1996 72	Dicot. sp. indet.	187	0	0	0	0
PB_1997_10	Dicot. sp., salicoid	13	0	0	0	0
PB_1997_1002	cf. Ternstroemites dentatus	17	0	0	0	0
PB_1997_1005	Pungiphyllum waltheri	17	0	0	0	0
PB_1997_1006	Pungiphyllum waltheri	44	0	0	0	0
PB_1997_1007	Pungiphyllum waltheri	17	0	0	0	0
PB_1997_101	Dicot. sp. indet.	12	0	0	0	0
PB_1997_1012	Juglandaceae sp.	16	0	0	0	0
PB_1997_1018	Juglandaceae sp.	16	0	0	0	0
PB_1997_1042	Juglandaceae sp.	12	0	0	0	0
PB_1997_1046	Juglandaceae sp.	14	0	0	0	0
PB_1997_1050	Juglandaceae sp.	12 15 34	0	0	0	0
PB_1997_1052	Juglandaceae sp.	2	0	1	0	0
PB_1997_1055	Juglandaceae sp.	80	0	0	0	0
PB_1997_1056	Juglandaceae sp.	12	0	0	0	0
PB_1997_1060	Juglandaceae sp.	36	0	0	0	0
PB_1997_1072	Juglandaceae sp.	12	0	0	0	0
PB_1997_1078	Juglandaceae sp.	14	0	0	0	0
PB_1997_1080	Juglandaceae sp.	12	0	0	0	0
PB_1997_1086	Juglandaceae sp.	12	0	0	0	0
PB_1997_1096	Juglandaceae sp.	12 17	0	0	0	0
PB_1997_11	Dicot. sp., salicoid	143	0	0	0	0
PB_1997_1106	Juglandaceae sp.	80	0	0	0	0
PB_1997_1108	Juglandaceae sp.	2	0	1	0	0
PB_1997_1139	Juglandaceae sp.	2	0	1	0	0
PB_1997_1150	Juglandaceae sp.	36	0	0	0	0
PB_1997_1160	Juglandaceae sp.	3	0	0	1	0
PB_1997_128	Dicot. sp. indet.	15	0	0	0	0
PB_1997_135	Dicot. sp. indet.	12	0	0	0	0
PB_1997_145	Dicot. sp. indet.	12	0	0	0	0
PB_1997_156	Lauraceae sp.	15 62 189 198	0	0	0	0
PB_1997_161	Dicot. sp. indet.	90	0	0	0	0
PB_1997_162	Dicot. sp. indet.	187	0	0	0	0
PB_1997_168	Dicot. sp. indet.	53	0	0	0	0
PB_1997_175	Dicot. sp. indet.	208	0	0	0	0
PB_1997_180	Dicot. sp. indet.	12	0	0	0	0
PB_1997_182	Dicot. sp. indet.	2	0	1	0	0
PB_1997_183	Anacardiaceae sp.	34	0	0	0	0
PB_1997_19	Juglandaceae sp.	187	0	0	0	0
PB_1997_192	Dicot. sp. indet.	2	0	1	0	0
PB_1997_20	Dicot. sp., salicoid	14 187	0	0	0	0
PB_1997_203	Dicot. sp. indet.	111	0	0	0	0
PB_1997_205	Dicot. sp. indet.	3 197	0	0	1	0
PB_1997_206	Dicot. sp. indet.	2	0	1	0	0
PB_1997_210	Dicot. sp. indet.	1	1	0	0	0
PB_1997_236	Dicot. sp., salicoid	12 33	0	0	0	0
PB_1997_250	Dicot. sp. indet.	2	0	1	0	0
PB_1997_265	Dicot. sp. indet.	12	0	0	0	0
PB_1997_271	Dicot. sp. indet.	12 80	0	0	0	0
PB_1997_273	Dicot. sp. indet.	12	0	0	0	0
PB_1997_285	Dicot. sp. indet.	12 43	0	0	0	0
PB_1997_292	Dicot. sp. indet.	14	0	0	0	0
PB_1997_295	Dicot. sp. indet.	3	0	0	1	0
PB_1997_296	Dicot. sp. indet.	6 142	0	0	0	0
PB_1997_367	Dicot. sp., vitioid	1 4 5 12	1	0	0	1
PB_1997_379	Dicot. sp., vitioid	12 37 186 194	0	0	0	0
PB_1997_403	Dicot. sp., vitioid	12 14	0	0	0	0
PB_1997_424	"Apocynophyllum sp."	14	0	0	0	0
PB_1997_452	"Apocynophyllum sp."	12	0	0	0	0
PB_1997_46	Lauraceae sp.	188	0	0	0	0
PB_1997_460	"Apocynophyllum sp."	3	0	0	1	0
PB_1997_464	"Apocynophyllum sp."	12	0	0	0	0
PB_1997_47	Rutaceae sp.	1 12 208	1	0	0	0
PB_1997_475	"Apocynophyllum sp."	12	0	0	0	0
PB_1997_481	"Apocynophyllum sp."	12	0	0	0	0
PB_1997_483	"Apocynophyllum sp."	14	0	0	0	0
PB_1997_486	"Apocynophyllum sp."	3	0	0	1	0
PB_1997_49	Dicot. sp. indet.	12 208	0	0	0	0
PB_1997_5	Dicot. sp., salicoid	14	0	0	0	0
PB_1997_527	"Apocynophyllum sp."	12	0	0	0	0
PB_1997_529	"Apocynophyllum sp."	13	0	0	0	0
PB_1997_531	"Apocynophyllum sp."	12	0	0	0	0
PB_1997_533	"Apocynophyllum sp."	12	0	0	0	0
PB_1997_535	"Apocynophyllum sp."	14	0	0	0	0
PB_1997_543	"Apocynophyllum sp."	12	0	0	0	0
PB_1997_560	"Apocynophyllum sp."	12	0	0	0	0
PB_1997_578	Dicot. sp., ulmoid	143	0	0	0	0
PB_1997_587	Dicot. sp., ulmoid	143	0	0	0	0
PB_1997_597	Dicot. sp., ulmoid	2	0	1	0	0
PB_1997_601	Dicot. sp., ulmoid	143	0	0	0	0
PB_1997_604	Dicot. sp., ulmoid	9	0	0	0	0
PB_1997_612	Dicot. sp., ulmoid	9	0	0	0	0
PB_1997_621	Dicot. sp., ulmoid	14	0	0	0	0
PB_1997_624	Dicot. sp., ulmoid	78	0	0	0	0
PB_1997_627	Dicot. sp., ulmoid	2	0	1	0	0
PB_1997_628	Dicot. sp., ulmoid	143	0	0	0	0
PB_1997_633	Dicot. sp., ulmoid	143	0	0	0	0
PB_1997_643	Dicot. sp., ulmoid	2	0	1	0	0
PB_1997_675	Dicot. sp., ulmoid	143	0	0	0	0
PB_1997_679	Dicot. sp., ulmoid	143	0	0	0	0
PB_1997_680	Dicot. sp., ulmoid	143	0	0	0	0
PB_1997_694	Dicot. sp., ulmoid	143	0	0	0	0
PB_1997_7	Juglandaceae sp.	81	0	0	0	0
PB_1997_722	Juglandaceae sp.	14	0	0	0	0
PB_1997_724	Juglandaceae sp.	144	0	0	0	0
PB_1997_725	Juglandaceae sp.	12	0	0	0	0
PB_1997_726	Juglandaceae sp.	80	0	0	0	0
PB_1997_727	Juglandaceae sp.	12	0	0	0	0
PB_1997_729	Juglandaceae sp.	3	0	0	1	0
PB_1997_730	Juglandaceae sp.	2	0	1	0	0
PB_1997_735	Juglandaceae sp.	12	0	0	0	0
PB_1997_737	Juglandaceae sp.	12	0	0	0	0
PB_1997_739	Juglandaceae sp.	2 12 198	0	1	0	0
PB_1997_741	Juglandaceae sp.	2	0	1	0	0
PB_1997_742	Juglandaceae sp.	144	0	0	0	0
PB_1997_743	Juglandaceae sp.	2	0	1	0	0
PB_1997_761	Juglandaceae sp.	2	0	1	0	0
PB_1997_769	Juglandaceae sp.	12 206	0	0	0	0
PB_1997_773	Juglandaceae sp.	12	0	0	0	0
PB_1997_782	Juglandaceae sp.	12	0	0	0	0
PB_1997_789	Juglandaceae sp.	12 202	0	0	0	0
PB_1997_792	Juglandaceae sp.	14	0	0	0	0
PB_1997_798	Juglandaceae sp.	12 195	0	0	0	0
PB_1997_800	Juglandaceae sp.	80	0	0	0	0
PB_1997_803	Juglandaceae sp.	12	0	0	0	0
PB_1997_807	Juglandaceae sp.	12	0	0	0	0
PB_1997_817	Juglandaceae sp.	80	0	0	0	0
PB_1997_837	Juglandaceae sp.	189	0	0	0	0
PB_1997_847	Juglandaceae sp.	12	0	0	0	0
PB_1997_850	Juglandaceae sp.	12 80	0	0	0	0
PB_1997_854	Juglandaceae sp.	12	0	0	0	0
PB_1997_866	Juglandaceae sp.	61	0	0	0	0
PB_1997_88	Dicot. sp. indet.	3	0	0	1	0
PB_1997_883	Juglandaceae sp.	12 13 206	0	0	0	0
PB_1997_891	Juglandaceae sp.	2	0	1	0	0
PB_1997_899	Juglandaceae sp.	12	0	0	0	0
PB_1997_901	Juglandaceae sp.	2	0	1	0	0
PB_1997_916	Juglandaceae sp.	12	0	0	0	0
PB_1997_929	Juglandaceae sp.	3 17	0	0	1	0
PB_1997_934	Juglandaceae sp.	12	0	0	0	0
PB_1997_952	Juglandaceae sp.	196	0	0	0	0
PB_1997_953	Juglandaceae sp.	12	0	0	0	0
PB_1997_961	Juglandaceae sp.	16	0	0	0	0
PB_1997_965	Juglandaceae sp.	12	0	0	0	0
PB_1997_968	Juglandaceae sp.	12	0	0	0	0
PB_1997_984	Juglandaceae sp.	12	0	0	0	0
PB_1997_989	Juglandaceae sp.	12	0	0	0	0
PB_2000 242	Pungiphyllum waltheri	80	0	0	0	0
PB_2000 256	Pungiphyllum waltheri	80	0	0	0	0
PB_2000 265	Pungiphyllum waltheri	14	0	0	0	0
PB_2000 310	cf. Ternstroemites dentatus	2	0	1	0	0
PB_2000 313	cf. Ternstroemites dentatus	12	0	0	0	0
PB_2000 328	cf. Ternstroemites dentatus	12	0	0	0	0
PB_2000_331	Dicot. sp., carpinoid	1	1	0	0	0
PB_2001 18	cf. Ternstroemites dentatus	12	0	0	0	0
PB_2001 82	Fabaceae sp.	81	0	0	0	0
PB_2003_1000	Dicot. sp. indet.	30	0	0	0	0
PB_2003_1002	Dicot. sp. indet.	12 13 142	0	0	0	0
PB_2003_1003	Juglandaceae sp.	12	0	0	0	0
PB_2003_1004	Myricaceae sp.	214	0	0	0	0
PB_2003_1006	Juglandaceae sp.	12	0	0	0	0
PB_2003_1007	Juglandaceae sp.	12	0	0	0	0
PB_2003_1008	Juglandaceae sp.	12	0	0	0	0
PB_2003_1009	Juglandaceae sp.	16	0	0	0	0
PB_2003_1011	Juglandaceae sp.	12	0	0	0	0
PB_2003_1012	Juglandaceae sp.	5 12 13	0	0	0	0
PB_2003_1013	Juglandaceae sp.	12	0	0	0	0
PB_2003_1014	Juglandaceae sp.	12 16	0	0	0	0
PB_2003_1016	Juglandaceae sp.	2	0	1	0	0
PB_2003_1017	Dicot. sp. indet.	2	0	1	0	0
PB_2003_1018	Dicot. sp. indet.	22	0	0	0	0
PB_2003_1022	Dicot. sp. indet.	14	0	0	0	0
PB_2003_1024	Dicot. sp. indet.	5 12 15	0	0	0	0
PB_2003_1025	Lauraceae sp.	2	0	1	0	0
PB_2003_1026	Dicot. sp., salicoid	208	0	0	0	0
PB_2003_1028	Dicot. sp., salicoid	12 14	0	0	0	0
PB_2003_1030	"Apocynophyllum sp."	2	0	1	0	0
PB_2003_1031	"Apocynophyllum sp."	15	0	0	0	0
PB_2003_1032	"Apocynophyllum sp."	12	0	0	0	0
PB_2003_280	Dicot. sp. indet.	104	0	0	0	0
PB_2003_288	Dicot. sp. indet.	12	0	0	0	0
PB_2003_453	Dicot. sp., "hallensis"	32	0	0	0	0
PB_2003_460	Dicot. sp., "hallensis"	2	0	1	0	0
PB_2003_462	Dicot. sp., "hallensis"	2	0	1	0	0
PB_2003_936	Dicot. sp. indet.	12 13	0	0	0	0
PB_2003_937	Dicot. sp. indet.	173	0	0	0	0
PB_2003_938	"Apocynophyllum sp."	14	0	0	0	0
PB_2003_939	Rutaceae sp.	5 13 14 208	0	0	0	0
PB_2003_940	Juglandaceae sp.	14	0	0	0	0
PB_2003_942	Dicot. sp. indet.	200	0	0	0	0
PB_2003_945	Dicot. sp. indet.	32	0	0	0	0
PB_2003_947	Lauraceae sp.	12	0	0	0	0
PB_2003_948	Juglandaceae sp.	168	0	0	0	0
PB_2003_949	Ulmaceae sp.	16 30	0	0	0	0
PB_2003_950	Dicot. sp. indet.	29	0	0	0	0
PB_2003_952	Juglandaceae sp.	17	0	0	0	0
PB_2003_953	Dicot. sp. indet.	12	0	0	0	0
PB_2003_954	Pungiphyllum waltheri	12 14	0	0	0	0
PB_2003_955	Dicot. sp. indet.	2	0	1	0	0
PB_2003_956	Dicot. sp. indet.	2	0	1	0	0
PB_2003_957	Juglandaceae sp.	3	0	0	1	0
PB_2003_958	Dicot. sp. indet.	12 14	0	0	0	0
PB_2003_959	Fabaceae sp.	2 12	0	1	0	0
PB_2003_960	Dicot. sp. indet.	5 14	0	0	0	0
PB_2003_962	Dicot. sp. indet.	2 16	0	1	0	0
PB_2003_963	Dicot. sp. indet.	12 79	0	0	0	0
PB_2003_964	Juglandaceae sp.	16	0	0	0	0
PB_2003_966	Dicot. sp. indet.	2 12	0	1	0	0
PB_2003_969	Dicot. sp. indet.	32	0	0	0	0
PB_2003_970	Dicot. sp. indet.	32	0	0	0	0
PB_2003_971	Dicot. sp. indet.	29 32	0	0	0	0
PB_2003_972	Dicot. sp. indet.	12 14 15	0	0	0	0
PB_2003_973	Fabaceae sp.	14 163	0	0	0	0
PB_2003_974	Ulmaceae sp.	14 16	0	0	0	0
PB_2003_975	"Apocynophyllum sp."	3 5	0	0	1	0
PB_2003_977	Dicot. sp. indet.	2	0	1	0	0
PB_2003_978	Juglandaceae sp.	5 14	0	0	0	0
PB_2003_979	Dicot. sp. indet.	12 32	0	0	0	0
PB_2003_980	"Apocynophyllum sp."	5	0	0	0	0
PB_2003_981	Rutaceae sp.	208	0	0	0	0
PB_2003_983	Dicot. sp. indet.	12	0	0	0	0
PB_2003_986	Dicot. sp. indet.	2 12	0	1	0	0
PB_2003_987	Juglandaceae sp.	17	0	0	0	0
PB_2003_989	Dicot. sp. indet.	188	0	0	0	0
PB_2003_990	Dicot. sp. indet.	32	0	0	0	0
PB_2003_991	Fabaceae sp.	81	0	0	0	0
PB_2003_992	"Apocynophyllum sp."	12	0	0	0	0
PB_2003_993	Ulmaceae sp.	1 2 3 12 8	1	1	1	0
PB_2003_994	Juglandaceae sp.	3 5	0	0	1	0
PB_2003_996	"Apocynophyllum sp."	3	0	0	1	0
PB_2003_998	cf. Ternstroemites dentatus	17	0	0	0	0
PB_2003_999	cf. Ternstroemites dentatus	16	0	0	0	0
PB_2005_10	Dicot. sp. indet.	188	0	0	0	0
PB_2005_100	Dicot. sp. indet.	32	0	0	0	0
PB_2005_102	Juglandaceae sp.	32	0	0	0	0
PB_2005_104	Lauraceae sp.	14 189	0	0	0	0
PB_2005_106	Dicot. sp. indet.	168	0	0	0	0
PB_2005_107	Dicot. sp. indet.	188	0	0	0	0
PB_2005_108	Juglandaceae sp.	32	0	0	0	0
PB_2005_109	Dicot. sp. indet.	188	0	0	0	0
PB_2005_11	Anacardiaceae sp.	14 32 188	0	0	0	0
PB_2005_110	Dicot. sp. indet.	16 62	0	0	0	0
PB_2005_112	Dicot. sp. indet.	144	0	0	0	0
PB_2005_113	Dicot. sp. indet.	12 36	0	0	0	0
PB_2005_115	Dicot. sp. indet.	62 194	0	0	0	0
PB_2005_118	Dicot. sp. indet.	186 194	0	0	0	0
PB_2005_12	Juglandaceae sp.	202	0	0	0	0
PB_2005_120	Dicot. sp. indet.	15 189	0	0	0	0
PB_2005_121	Fabaceae sp.	163	0	0	0	0
PB_2005_124	Dicot. sp. indet.	62	0	0	0	0
PB_2005_125	Juglandaceae sp.	32	0	0	0	0
PB_2005_126	Dicot. sp. indet.	32	0	0	0	0
PB_2005_127	Dicot. sp., salicoid	189	0	0	0	0
PB_2005_128	Fabaceae sp.	12 34	0	0	0	0
PB_2005_13	Dicot. sp. indet.	192	0	0	0	0
PB_2005_130	Moraceae sp.	62	0	0	0	0
PB_2005_131	Juglandaceae sp.	62	0	0	0	0
PB_2005_133	Dicot. sp. indet.	32	0	0	0	0
PB_2005_134	Rosaceae sp.	62	0	0	0	0
PB_2005_135	Dicot. sp. indet.	12 62	0	0	0	0
PB_2005_136	Juglandaceae Formenkreis 1	1 3 62	1	0	1	0
PB_2005_137	Lauraceae sp.	3 12 62	0	0	1	0
PB_2005_138	Lauraceae sp.	188	0	0	0	0
PB_2005_139	Dicot. sp. indet.	188	0	0	0	0
PB_2005_140	Dicot. sp. indet.	1 191	1	0	0	0
PB_2005_142	Dicot. sp. indet.	144	0	0	0	0
PB_2005_143	Juglandaceae sp.	36	0	0	0	0
PB_2005_144	Dicot. sp. indet.	14	0	0	0	0
PB_2005_145	Dicot. sp. indet.	14	0	0	0	0
PB_2005_146	Dicot. sp. indet.	14	0	0	0	0
PB_2005_147	Dicot. sp. indet.	12	0	0	0	0
PB_2005_148	Dicot. sp. indet.	12	0	0	0	0
PB_2005_149	Juglandaceae sp.	14	0	0	0	0
PB_2005_15	Moraceae sp.	8 15 208	0	0	0	0
PB_2005_150	Juglandaceae sp.	14	0	0	0	0
PB_2005_151	Juglandaceae sp.	16	0	0	0	0
PB_2005_152	Dicot. sp. indet.	173	0	0	0	0
PB_2005_153	Dicot. sp. indet.	12	0	0	0	0
PB_2005_154	Lauraceae sp.	13	0	0	0	0
PB_2005_155	Lauraceae sp.	12 34	0	0	0	0
PB_2005_156	Juglandaceae sp.	1 3 12 80	1	0	1	0
PB_2005_157	Dicot. sp., salicoid	14	0	0	0	0
PB_2005_158	Dicot. sp., salicoid	5 13 14	0	0	0	0
PB_2005_159	Dicot. sp., ulmoid	143	0	0	0	0
PB_2005_16	Dicot. sp. indet.	171	0	0	0	0
PB_2005_160	Dicot. sp., ulmoid	4 13	0	0	0	1
PB_2005_161	Juglandaceae sp.	12	0	0	0	0
PB_2005_162	Juglandaceae sp.	14	0	0	0	0
PB_2005_163	Juglandaceae sp.	83	0	0	0	0
PB_2005_164	Juglandaceae sp.	12	0	0	0	0
PB_2005_165	Juglandaceae sp.	12	0	0	0	0
PB_2005_166	Juglandaceae sp.	3	0	0	1	0
PB_2005_167	Juglandaceae sp.	14	0	0	0	0
PB_2005_168	Juglandaceae sp.	12	0	0	0	0
PB_2005_169	Juglandaceae sp.	37	0	0	0	0
PB_2005_17	Dicot. sp. indet.	208	0	0	0	0
PB_2005_170	Juglandaceae sp.	2	0	1	0	0
PB_2005_171	Dicot. sp. indet.	34	0	0	0	0
PB_2005_172	Juglandaceae sp.	3	0	0	1	0
PB_2005_173	Juglandaceae sp.	12	0	0	0	0
PB_2005_174	Juglandaceae sp.	12	0	0	0	0
PB_2005_175	Juglandaceae sp.	2	0	1	0	0
PB_2005_176	Juglandaceae sp.	13	0	0	0	0
PB_2005_177	Dicot. sp. indet.	7	0	0	0	0
PB_2005_178	Juglandaceae sp.	14	0	0	0	0
PB_2005_179	Dicot. sp. indet.	144	0	0	0	0
PB_2005_18	Dicot. sp. indet.	15 189	0	0	0	0
PB_2005_180	Dicot. sp. indet.	3	0	0	1	0
PB_2005_181	Dicot. sp. indet.	201	0	0	0	0
PB_2005_182	Lauraceae sp.	12	0	0	0	0
PB_2005_183	Lauraceae sp.	2	0	1	0	0
PB_2005_184	Dicot. sp., salicoid	9	0	0	0	0
PB_2005_185	Dicot. sp., salicoid	12	0	0	0	0
PB_2005_186	Dicot. sp., salicoid	14 34	0	0	0	0
PB_2005_187	Dicot. sp., salicoid	12	0	0	0	0
PB_2005_188	Dicot. sp. indet.	189	0	0	0	0
PB_2005_189	Dicot. sp. indet.	12	0	0	0	0
PB_2005_19	Juglandaceae sp.	163	0	0	0	0
PB_2005_190	Dicot. sp. indet.	2	0	1	0	0
PB_2005_191	Dicot. sp. indet.	2	0	1	0	0
PB_2005_192	Dicot. sp. indet.	144	0	0	0	0
PB_2005_193	Dicot. sp. indet.	92	0	0	0	0
PB_2005_194	Juglandaceae sp.	12	0	0	0	0
PB_2005_195	Dicot. sp. indet.	201	0	0	0	0
PB_2005_196	Dicot. sp. indet.	14	0	0	0	0
PB_2005_197	Dicot. sp., salicoid	12	0	0	0	0
PB_2005_198	Dicot. sp., salicoid	12	0	0	0	0
PB_2005_199	Dicot. sp., salicoid	12	0	0	0	0

Summary data for the Eckfeld maar locality.

PB_2005_20	Dicot. sp. indet.	187				
PB_2005_200	Dicot. sp., salicoid	145				
PB_2005_201	Dicot. sp., salicoid	12				
PB_2005_202	Dicot. sp., salicoid	145				
PB_2005_203	Dicot. sp., salicoid	12				
PB_2005_204	Dicot. sp. indet.	145				
PB_2005_205	Dicot. sp., salicoid	12				
PB_2005_206	Dicot. sp. indet.	144				
PB_2005_207	Dicot. sp. indet.	145				
PB_2005_208	Dicot. sp. indet.	14				
PB_2005_209	Dicot. sp. indet.	186				
PB_2005_21	Dicot. sp. indet.	12	187			
PB_2005_210	Dicot. sp. indet.	12				
PB_2005_211	Dicot. sp. indet.	144				
PB_2005_212	Dicot. sp. indet.	12				
PB_2005_213	Dicot. sp. indet.	2				
PB_2005_214	Dicot. sp. indet.	12				
PB_2005_215	Dicot. sp. indet.	143				
PB_2005_216	Dicot. sp. indet.	12	29			
PB_2005_217	Dicot. sp. indet.	12				
PB_2005_218	Dicot. sp. indet.	11				
PB_2005_219	Dicot. sp. indet.	3				
PB_2005_22	Lauraceae sp.	187				
PB_2005_220	Lauraceae sp.	2				
PB_2005_221	Dicot. sp. indet.	13				
PB_2005_222	Dicot. sp. indet.	12				
PB_2005_223	Dicot. sp. indet.	4				
PB_2005_224	Juglandaceae sp.	12	34			
PB_2005_225	Dicot. sp. indet.	2				
PB_2005_226	Dicot. sp. indet.	2				
PB_2005_227	Dicot. sp. indet.	190				
PB_2005_228	Dicot. sp. indet.	62				
PB_2005_229	Dicot. sp. indet.	2				
PB_2005_23	Dicot. sp. indet.	187				
PB_2005_230	Dicot. sp. indet.	13				
PB_2005_231	Dicot. sp. indet.	14				
PB_2005_232	Dicot. sp. indet.	12	187			
PB_2005_233	Dicot. sp. indet.	3				
PB_2005_234	Dicot. sp. indet.	170				
PB_2005_235	Dicot. sp. indet.	12				
PB_2005_236	Dicot. sp. indet.	12				
PB_2005_237	Dicot. sp. indet.	2				
PB_2005_238	Dicot. sp. indet.	12				
PB_2005_239	Dicot. sp. indet.	12				
PB_2005_24	Dicot. sp. indet.	187				
PB_2005_240	Lauraceae sp.	12				
PB_2005_241	Lauraceae sp.	12				
PB_2005_242	Dicot. sp. indet.	16				
PB_2005_243	Lauraceae sp.	144				
PB_2005_244	Dicot. sp. indet.	80				
PB_2005_245	Dicot. sp. indet.	14				
PB_2005_246	Dicot. sp. indet.	12				
PB_2005_247	Dicot. sp. indet.	6				
PB_2005_248	Dicot. sp. indet.	12				
PB_2005_249	Lauraceae sp.	1	33			
PB_2005_25	Dicot. sp. indet.	80				
PB_2005_250	Dicot. sp. indet.	14				
PB_2005_251	Dicot. sp. indet.	12	203			
PB_2005_252	Dicot. sp. indet.	14				
PB_2005_253	Dicot. sp. indet.	15				
PB_2005_254	Cercidiphyllum sp.	2				
PB_2005_255	Cercidiphyllum sp.	2				
PB_2005_256	Dicot. sp. indet.	12				
PB_2005_257	Dicot. sp. indet.	12				
PB_2005_258	Dicot. sp. indet.	3				
PB_2005_259	Dicot. sp. indet.	30				
PB_2005_26	Dicot. sp. indet.	208				
PB_2005_260	Lauraceae sp.	14				
PB_2005_261	Dicot. sp. indet.	2				
PB_2005_262	Dicot. sp. indet.	12				
PB_2005_263	Dicot. sp. indet.	187				
PB_2005_264	Dicot. sp. indet.	81				
PB_2005_265	Pungiphyllum waltheri	14				
PB_2005_266	Dicot. sp. indet.	14				
PB_2005_267	Dicot. sp. indet.	12				
PB_2005_268	Dicot. sp. indet.	12				
PB_2005_269	Ficus sp.	2	12			
PB_2005_27	Juglandaceae sp.	208				
PB_2005_270	Juglandaceae sp.	12				
PB_2005_271	Rosaceae sp.	12				
PB_2005_272	Juglandaceae sp.	144				
PB_2005_273	Dicot. sp. indet.	32				
PB_2005_274	Juglandaceae sp.	2				
PB_2005_275	Dicot. sp. indet.	4	90			
PB_2005_276	Dicot. sp. indet.	81				
PB_2005_277	Juglandaceae sp.	15				
PB_2005_278	Juglandaceae sp.	14				
PB_2005_279	Juglandaceae sp.	6				
PB_2005_28	Laurophyllum sp.	200				
PB_2005_280	Pungiphyllum waltheri	33				
PB_2005_281	Pungiphyllum waltheri	33				
PB_2005_282	cf. Ternstroemites dentatus	12				
PB_2005_283	Dicot. sp. indet.	2				
PB_2005_284	Juglandaceae sp.	36				
PB_2005_285	Dicot. sp. indet.	2				
PB_2005_286	Dicot. sp. indet.	12				
PB_2005_289	Dicot. sp. indet.	2				
PB_2005_29	Dicot. sp. indet.	30	32			
PB_2005_290	Juglandaceae sp.	14				
PB_2005_291	Dicot. sp. indet.	6				
PB_2005_292	Dicot. sp. indet.	145				
PB_2005_293	Dicot. sp. indet.	1	3			
PB_2005_294	Dicot. sp. indet.	12				
PB_2005_295	Lauraceae sp.	12				
PB_2005_296	Ulmaceae sp.	1				
PB_2005_297	Juglandaceae sp.	1				
PB_2005_298	Juglandaceae sp.	3				
PB_2005_299	Dicot. sp. indet.	13				
PB_2005_3	Dicot. sp. indet.	208				
PB_2005_30	Lauraceae sp.	32				
PB_2005_300	Pungiphyllum waltheri	12				
PB_2005_301	Juglandaceae sp.	14	2			
PB_2005_302	Fabaceae sp.	12	30			
PB_2005_303	Juglandaceae sp.	142				
PB_2005_304	Dicot. sp. indet.	12				
PB_2005_305	Dicot. sp. indet.	38				
PB_2005_306	Juglandaceae sp.	30				
PB_2005_307	Juglandaceae sp.	12				
PB_2005_308	Lauraceae sp.	36				
PB_2005_309	Dicot. sp. indet.	2				
PB_2005_31	Anacardiaceae sp.	62				
PB_2005_310	Dicot. sp. indet.	12				
PB_2005_311	Dicot. sp. indet.	2				
PB_2005_312	cf. Ternstroemites dentatus	36				
PB_2005_313	Vitaceae sp.	12				
PB_2005_314	Rutaceae sp.	90				
PB_2005_315	Juglandaceae sp.	9				
PB_2005_316	Dicot. sp. indet.	12				
PB_2005_317	Juglandaceae sp.	12				
PB_2005_318	Dicot. sp. indet.	90				
PB_2005_319	Dicot. sp. indet.	12				
PB_2005_32	Juglandaceae sp.	12	190			
PB_2005_320	Dicot. sp. indet.	38				
PB_2005_321	Dicot. sp. indet.	12				
PB_2005_322	Dicot. sp. indet.	5				
PB_2005_323	"Apocynophyllum sp."	2				
PB_2005_324	Carpinus sp.	3	32			
PB_2005_325	Dicot. sp. indet.	90				
PB_2005_326	Lauraceae sp.	169				
PB_2005_327	Dicot. sp. indet.	12				
PB_2005_328	Lauraceae sp.	14				
PB_2005_329	Ulmaceae sp.	202				
PB_2005_33	Dicot. sp., salicoid	1	2	29	3	
PB_2005_330	Dicot. sp. indet.	2				
PB_2005_331	Dicot. sp. indet.	188				
PB_2005_332	Dicot. sp. indet.	30				
PB_2005_333	Dicot. sp. indet.	12				
PB_2005_334	Dicot. sp. indet.	3				
PB_2005_335	Lauraceae sp.	30				
PB_2005_336	Juglandaceae sp.	2				
PB_2005_337	Dicot. sp., "hallensis"	171				
PB_2005_338	Juglandaceae sp.	12				
PB_2005_339	Dicot. sp. indet.	1	3	15		
PB_2005_34	Juglandaceae sp.	12				
PB_2005_340	Buxaceae sp.	15				
PB_2005_341	Fabaceae sp.	1				
PB_2005_342	Dicot. sp. indet.	3				
PB_2005_343	Dicot. sp. indet.	186				
PB_2005_344	Dicot. sp. indet.	3				
PB_2005_345	Dicot. sp. indet.	198				
PB_2005_346	Dicot. sp. indet.	12				
PB_2005_347	Ulmaceae sp.	1				
PB_2005_348	Lauraceae sp.	3	12	14		
PB_2005_349	Lauraceae sp.	2	17			
PB_2005_35	Laurophyllum sp.	12	13			
PB_2005_350	"Apocynophyllum sp."	2				
PB_2005_351	Dicot. sp. indet.	81				
PB_2005_352	Dicot. sp. indet.	32				
PB_2005_353	"Apocynophyllum sp."	14				
PB_2005_354	Fabaceae sp.	12				
PB_2005_355	Dicot. sp. indet.	12				
PB_2005_356	Dicot. sp. indet.	3				
PB_2005_357	Juglandaceae sp.	14				
PB_2005_358	Juglandaceae sp.	14				
PB_2005_359	Dicot. sp. indet.	12				
PB_2005_36	Dicot. sp. indet.	104				
PB_2005_360	Dicot. sp. indet.	14				
PB_2005_361	Dicot. sp. indet.	14				
PB_2005_362	Dicot. sp. indet.	14				
PB_2005_363	Juglandaceae sp.	1				
PB_2005_364	Juglandaceae sp.	1				
PB_2005_365	Dicot. sp. indet.	2				
PB_2005_366	"Apocynophyllum sp."	2				
PB_2005_367	Lauraceae sp.	12				
PB_2005_368	Dicot. sp. indet.	3				
PB_2005_369	Juglandaceae sp.	3				
PB_2005_37	Juglandaceae sp.	13				
PB_2005_370	Lauraceae sp.	12				
PB_2005_371	Dicot. sp. indet.	16				
PB_2005_372	Dicot. sp. indet.	2				
PB_2005_373	Dicot. sp. indet.	145				
PB_2005_374	Juglandaceae sp.	1				
PB_2005_376	Dicot. sp. indet.	2				
PB_2005_377	Dicot. sp. indet.	197				
PB_2005_378	Juglandaceae sp.	2				
PB_2005_379	Dicot. sp. indet.	14				
PB_2005_38	Dicot. sp. indet.	12	32			
PB_2005_380	Dicot. sp. indet.	12				
PB_2005_381	Dicot. sp. indet.	1				
PB_2005_382	Dicot. sp. indet.	163				
PB_2005_383	Dicot. sp. indet.	32				
PB_2005_384	Juglandaceae sp.	1				
PB_2005_385	Dicot. sp. indet.	2				
PB_2005_386	Juglandaceae sp.	17				
PB_2005_387	Ulmaceae sp.	9				
PB_2005_388	Dicot. sp. indet.	32				
PB_2005_389	Dicot. sp., "hallensis"	168				
PB_2005_39	Juglandaceae sp.	26				
PB_2005_390	Lauraceae sp.	2				
PB_2005_391	Juglandaceae sp.	80				
PB_2005_392	Rutaceae sp.	145				
PB_2005_393	"Apocynophyllum sp."	1	12	103		
PB_2005_394	"Apocynophyllum sp."	1				
PB_2005_395	"Apocynophyllum sp."	3				
PB_2005_396	"Apocynophyllum sp."	2	12	63		
PB_2005_397	"Apocynophyllum sp."	12				
PB_2005_399	Rutaceae sp.	1				
PB_2005_4	Lauraceae sp.	5	15	62		
PB_2005_40	Juglandaceae sp.	12				
PB_2005_400	Fabaceae sp.	12				
PB_2005_401	Lauraceae sp.	12				
PB_2005_402	Lauraceae sp.	2				
PB_2005_403	"Apocynophyllum sp."	12				
PB_2005_404	Dicot. sp. indet.	144				
PB_2005_405	Dicot. sp. indet.	144				
PB_2005_406	Dicot. sp. indet.	144				
PB_2005_407	Dicot. sp. indet.	144				
PB_2005_408	Dicot. sp. indet.	144				
PB_2005_409	Dicot. sp. indet.	144				
PB_2005_41	Rosaceae sp.	14				
PB_2005_410	Dicot. sp. indet.	144				
PB_2005_411	Dicot. sp. indet.	191				
PB_2005_412	Dicot. sp. indet.	2				
PB_2005_413	Lauraceae sp.	4				
PB_2005_414	Dicot. sp. indet.	14				
PB_2005_415	Juglandaceae sp.	192				
PB_2005_416	Dicot. sp. indet.	187				
PB_2005_417	Fabaceae sp.	14				
PB_2005_418	Dicot. sp. indet.	41				
PB_2005_419	Juglandaceae sp.	80				
PB_2005_42	Dicot. sp. indet.	17				
PB_2005_420	Dicot. sp. indet.	14				
PB_2005_421	Dicot. sp. indet.	12				
PB_2005_422	Juglandaceae sp.	12				
PB_2005_423	Dicot. sp. indet.	1				
PB_2005_424	Dicot. sp., "hallensis"	12	168			
PB_2005_425	Juglandaceae sp.	12	13			
PB_2005_426	Dicot. sp. indet.	12	54			
PB_2005_427	Rosaceae sp.	12				
PB_2005_428	Dicot. sp. indet.	12				
PB_2005_429	Dicot. sp. indet.	145				
PB_2005_43	Juglandaceae sp.	14				
PB_2005_430	Dicot. sp. indet.	12				
PB_2005_431	Dicot. sp. indet.	145				
PB_2005_433	Dicot. sp. indet.	144				
PB_2005_434	Dicot. sp. indet.	32				
PB_2005_435	Juglandaceae sp.	12				
PB_2005_436	Fabaceae sp.	168				
PB_2005_437	Dicot. sp. indet.	32				
PB_2005_438	Dicot. sp. indet.	12				
PB_2005_439	Dicot. sp. indet.	144				
PB_2005_44	Dicot. sp. indet.	12	16			
PB_2005_441	Dicot. sp. indet.	43				
PB_2005_442	Dicot. sp. indet.	14				
PB_2005_443	Lauraceae sp.	4				
PB_2005_444	Dicot. sp. indet.	12				
PB_2005_445	Juglandaceae sp.	14				
PB_2005_446	Dicot. sp. indet.	12				
PB_2005_447	Pungiphyllum waltheri	14				
PB_2005_448	Lauraceae sp.	38				
PB_2005_449	Juglandaceae sp.	12				
PB_2005_45	Juglandaceae sp.	12	16	90		
PB_2005_450	Dicot. sp., salicoid	197				
PB_2005_451	Lauraceae sp.	169				
PB_2005_452	Lauraceae sp.	169				
PB_2005_453	Dicot. sp. indet.	12	192			
PB_2005_454	Dicot. sp. indet.	36				
PB_2005_455	Dicot. sp. indet.	12	14			
PB_2005_457	Juglandaceae sp.	12				
PB_2005_459	Juglandaceae sp.	2	12			
PB_2005_46	Dicot. sp. indet.	143				
PB_2005_460	Dicot. sp. indet.	5	188			
PB_2005_461	Dicot. sp. indet.	12				
PB_2005_462	Fabaceae sp.	12				
PB_2005_463	Juglandaceae sp.	32				
PB_2005_464	Dicot. sp. indet.	2				
PB_2005_465	Fabaceae sp.	12				
PB_2005_466	Dicot. sp. indet.	14				
PB_2005_467	cf. Ternstroemites dentatus	29				
PB_2005_468	Dicot. sp. indet.	12				
PB_2005_469	Dicot. sp. indet.	145				
PB_2005_47	Dicot. sp. indet.	36				
PB_2005_470	Juglandaceae sp.	12				
PB_2005_471	Juglandaceae sp.	12				
PB_2005_472	Juglandaceae sp.	1				
PB_2005_473	cf. Ternstroemites dentatus	12				
PB_2005_474	Juglandaceae sp.	12	13			
PB_2005_475	Juglandaceae sp.	80				
PB_2005_476	Dicot. sp. indet.	12				
PB_2005_478	Ulmaceae sp.	12				
PB_2005_479	Juglandaceae sp.	12				
PB_2005_48	Lauraceae sp.	5	12	14		
PB_2005_480	Dicot. sp. indet.	2				
PB_2005_481	Dicot. sp. indet.	12				
PB_2005_483	Dicot. sp. indet.	8				
PB_2005_484	Dicot. sp. indet.	2	12			
PB_2005_485	"Apocynophyllum sp."	3				
PB_2005_486	Dicot. sp. indet.	189				
PB_2005_487	Juglandaceae sp.	80				
PB_2005_488	"Apocynophyllum sp."	12				
PB_2005_489	Dicot. sp., vitioid	111				
PB_2005_49	Juglandaceae sp.	1	3	38		
PB_2005_5	Lauraceae sp.	171				
PB_2005_50	Dicot. sp. indet.	30				
PB_2005_51	Juglandaceae sp.	14	32			
PB_2005_52	Dicot. sp. indet.	32				
PB_2005_53	Lauraceae sp.	11	12	113		
PB_2005_54	Laurophyllum sp.	120				
PB_2005_55	Dicot. sp. indet.	13	80	85		
PB_2005_56	Dicot. sp. indet.	3	187			
PB_2005_57	Dicot. sp. indet.	27	12	3	2	92
PB_2005_58	cf. Ternstroemites dentatus	15				
PB_2005_59	Dicot. sp. indet.	32				
PB_2005_60	Juglandaceae sp.	16				
PB_2005_61	Juglandaceae sp.	16				
PB_2005_63	Dicot. sp. indet.	14				
PB_2005_64	Ulmaceae sp.	2				
PB_2005_65	Juglandaceae sp.	78				
PB_2005_66	Dicot. sp. indet.	12				
PB_2005_67	Fabaceae sp.	81				
PB_2005_68	Dicot. sp. indet.	12				
PB_2005_69	Fabaceae sp.	32				
PB_2005_7	Lauraceae sp.	15	104			
PB_2005_70	Lauraceae sp.	186				
PB_2005_71	Dicot. sp. indet.	1	3	15		
PB_2005_72	Dicot. sp. indet.	1	3	15		
PB_2005_73	Dicot. sp. indet.	7				
PB_2005_74	Dicot. sp. indet.	2				
PB_2005_75	Dicot. sp. indet.	1	14	188		
PB_2005_76	Juglandaceae sp.	14				
PB_2005_77	Dicot. sp. indet.	1				
PB_2005_78	cf. Ternstroemites dentatus	15				
PB_2005_79	Dicot. sp. indet.	3	5	26		
PB_2005_8	Dicot. sp. indet.	208				
PB_2005_80	Dicot. sp. indet.	5	16	29		
PB_2005_81	Juglandaceae sp.	12				
PB_2005_82	Juglandaceae sp.	81				
PB_2005_83	Dicot. sp. indet.	14				
PB_2005_84	Juglandaceae sp.	2	143			
PB_2005_85	Dicot. sp. indet.	12				
PB_2005_86	Lauraceae sp.	12				
PB_2005_87	Dicot. sp. indet.	81				
PB_2005_88	Juglandaceae sp.	2				
PB_2005_89	Juglandaceae sp.	1	3	38		
PB_2005_9	Juglandaceae sp.	189				
PB_2005_90	Dicot. sp. indet.	16				
PB_2005_91	Dicot. sp. indet.	12				
PB_2005_92	Juglandaceae sp.	16				
PB_2005_93	Juglandaceae sp.	2	12			
PB_2005_94	Dicot. sp. indet.	189				
PB_2005_95	Juglandaceae sp.	34				
PB_2005_96	Dicot. sp. indet.	5	12	14		
PB_2005_97	Juglandaceae sp.	163				
PB_2005_98	Juglandaceae sp.	32				
PB_2005_99	Dicot. sp. indet.	190				
PE_1990_527	Dicot. sp. indet.	81				

Summary data for the Eckfeld maar locality.

DT 181	DT 182	DT 183	DT 184	DT 185	DT 186	DT 187	DT 188	DT 189	DT 190	DT 191	DT 192	DT 193	DT 194	DT 195	DT 196	DT 197	DT 198	DT 199	DT 200	DT 201	DT 202	DT 203	DT 204	DT 205	DT 206	DT 207	DT 208	DT 209	DT 210	DT 211	DT 212	DT 213	DT 214	DT 215	DT 216	DT 217	DT 218	DT 219	DT 220	DT 221	DT 222	DT 223
0	0	0	0	0	0	0	0	0	0	0	0	0	0	0	0	0	0	0	0	0	0	0	0	0	0	0	0	0	0	0	0	0	0	0	0	0	0	0	0	0	0	0
0	0	0	0	0	0	0	0	1	0	0	0	0	0	0	0	0	0	0	0	0	0	0	0	0	0	0	0	0	0	0	0	0	0	0	0	0	0	0	0	0	0	0
0	0	0	0	0	0	0	0	0	0	0	0	0	0	0	0	0	0	0	0	0	0	0	0	0	0	0	0	0	0	0	0	0	0	0	0	0	0	0	0	0	0	0
0	0	0	0	0	0	0	0	0	0	0	0	0	0	0	0	0	0	0	0	0	0	0	0	0	0	0	0	0	0	0	0	0	0	0	0	0	0	0	0	0	0	0
0	0	0	0	0	0	0	0	0	0	0	0	0	0	0	0	0	0	0	0	0	0	0	0	0	1	0	0	0	0	0	0	0	0	0	0	0	0	0	0	0	0	0
0	0	0	0	0	0	0	0	0	0	0	0	0	0	0	0	0	0	0	0	0	0	0	0	0	0	0	0	0	0	0	0	0	0	0	0	0	0	0	0	0	0	0
0	0	0	0	0	0	0	0	0	0	0	0	0	0	0	0	0	0	0	0	0	0	0	0	0	0	0	0	0	0	0	0	0	0	0	0	0	0	0	0	0	0	0
0	0	0	0	0	0	0	0	0	0	0	0	0	0	0	0	0	0	0	0	0	0	0	0	0	0	0	0	0	0	0	0	0	0	0	0	0	0	0	0	0	0	0
0	0	0	0	0	0	0	0	0	0	0	0	0	0	0	0	0	0	0	0	0	0	0	0	0	0	0	0	0	0	0	0	0	0	0	0	0	0	0	0	0	0	0
0	0	0	0	0	0	0	0	0	0	0	0	0	0	0	0	0	0	0	0	0	0	0	0	0	0	0	0	0	0	0	0	0	0	0	0	0	0	0	0	0	0	0
0	0	0	0	0	0	0	0	1	0	0	0	0	0	0	0	0	0	0	0	0	0	0	0	0	0	0	0	0	0	0	0	0	0	0	0	0	0	0	0	0	0	0
0	0	0	0	0	0	0	0	0	1	0	0	0	0	0	0	0	0	0	0	0	0	0	0	0	0	0	0	0	0	0	0	0	0	0	0	0	0	0	0	0	0	0
0	0	0	0	0	1	0	0	0	0	0	0	0	0	0	0	0	0	0	0	0	0	0	0	0	0	0	0	0	0	0	0	0	0	0	0	0	0	0	0	0	0	0
0	0	0	0	0	0	0	0	0	0	0	0	0	0	0	0	0	0	0	0	0	0	0	0	0	1	0	0	0	0	0	0	0	0	0	0	0	0	0	0	0	0	0
0	0	0	0	0	0	0	0	0	0	0	0	0	0	0	0	0	0	0	0	0	0	0	0	0	0	0	0	0	0	0	0	0	0	0	0	0	0	0	0	0	0	0
0	0	0	0	0	0	0	0	0	0	0	0	0	0	0	0	0	0	0	0	0	0	0	0	0	0	0	0	0	0	0	0	0	0	0	0	0	0	0	0	0	0	0
0	0	0	0	0	0	0	0	0	0	0	0	0	0	0	0	0	0	0	0	0	0	0	0	0	0	0	0	0	0	0	0	0	0	0	0	0	0	0	0	0	0	0
0	0	0	0	0	0	0	0	0	0	0	0	0	0	0	0	0	0	0	0	0	0	0	0	0	0	0	0	0	0	0	0	0	0	0	0	0	0	0	0	0	0	0
0	0	0	0	0	0	0	0	0	0	0	0	0	0	0	0	0	0	0	0	0	0	0	0	0	0	0	0	0	0	0	0	0	0	0	0	0	0	0	0	0	0	0
0	0	0	0	0	0	0	0	1	0	0	0	0	0	0	0	0	0	0	0	0	0	0	0	0	0	0	0	0	0	0	0	0	0	0	0	0	0	0	0	0	0	0
0	0	0	0	0	0	0	0	0	0	0	0	0	0	0	0	0	0	0	0	0	0	0	0	0	0	0	0	0	0	0	0	0	0	0	0	0	0	0	0	0	0	0
0	0	0	0	0	0	0	0	1	0	0	0	0	0	0	0	0	0	0	0	0	0	0	0	0	0	0	0	0	0	0	0	0	0	0	0	0	0	0	0	0	0	0
0	0	0	0	0	0	0	0	0	0	0	0	0	0	0	0	0	0	0	0	0	0	0	0	0	0	0	0	0	0	0	0	0	0	0	0	0	0	0	0	0	0	0
0	0	0	0	0	0	0	0	0	0	0	0	0	0	0	0	0	0	0	0	0	0	0	0	0	0	0	0	0	0	0	0	0	0	0	0	0	0	0	0	0	0	0
0	0	0	0	0	0	0	0	0	0	0	0	0	0	0	0	0	0	0	0	0	0	0	0	0	0	0	0	0	0	0	0	0	0	0	0	0	0	0	0	0	0	0
0	0	0	0	0	0	0	0	0	0	0	0	0	0	0	0	0	0	0	0	0	0	0	0	0	0	0	0	0	0	0	0	0	0	0	0	0	0	0	0	0	0	0
0	0	0	0	0	0	0	0	0	0	0	0	0	0	0	0	0	0	0	0	0	0	0	0	0	0	0	0	0	0	0	0	0	0	0	0	0	0	0	0	0	0	0
0	0	0	0	0	0	0	0	0	0	0	0	0	0	0	0	0	0	0	0	0	0	0	0	0	0	0	0	0	0	0	0	0	0	0	0	0	0	0	0	0	0	0
0	0	0	0	0	0	0	0	0	0	0	0	0	0	0	0	0	0	0	0	0	0	0	0	0	0	0	0	0	0	0	0	0	0	0	0	0	0	0	0	0	0	0
0	0	0	0	0	0	0	0	0	0	0	0	0	0	0	0	0	0	0	0	0	0	0	0	0	0	0	0	0	0	0	0	0	0	0	0	0	0	0	0	0	0	0
0	0	0	0	0	0	0	0	0	0	0	0	0	0	0	0	0	0	0	0	0	0	0	0	0	0	0	0	0	0	0	0	0	0	0	0	0	0	0	0	0	0	0
0	0	0	0	0	0	0	0	0	0	0	0	0	0	0	0	0	0	0	0	0	0	0	0	0	0	0	0	0	0	0	0	0	0	0	0	0	0	0	0	0	0	0

Summary data for the Eckfeld maar locality.
