# Supplementary material for: Testing for the Effects and Consequences of Mid Paleogene Climate Change on Insect Herbivory
Source: PLoS One. 2012 Jul 18;7(7):e40744. doi: 10.1371/journal.pone.0040744 (PMC3399891; doi:10.1371/journal.pone.0040744)
Supplement: File S3 — New damage types (DTs). (PDF) [file pone.0040744.s003.pdf]

Testing for the Effects and Consequences of Mid Paleogene Climate Change on Insect Herbivory

Torsten Wappler[1*], Conrad C. Labandeira[2,3], Jes Rust[1], Herbert Frankenhäuser[4], Volker Wilde[5]

1 Steinmann Institute, University of Bonn, 53115 Bonn, Germany; **2** Department of Paleobiology, National Museum of Natural History, Smithsonian Institution, Washington, DC 20013, USA; **3** Department of Entomology and BEES Program, University of Maryland, College Park, MD 20742, USA; **4** Mainz Natural History Museum / State Collection for Natural History of Rhineland-Palatine, Mainz, Germany; **5** Senckenberg Forschungsinstitut und Naturmuseum, Paläobotanik, Frankfurt am Main, Germany.

***** Author for correspondence.

ELECTRONIC SUPPLEMENTARY MATERIAL

FILE S3

New damage types (DTs) recorded from Messel and Eckfeld and types that are new but common at both localities organized by functional feeding groups.

Galling

Galling

DT 144 Galling

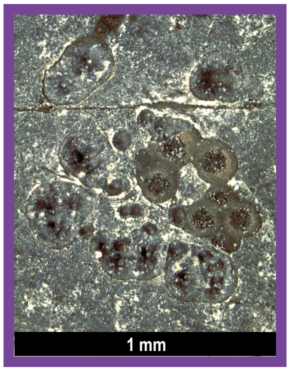

Multiloculate; dark inner core, surrounding smooth surface and circular margins; often fused.

DT 145 Galling

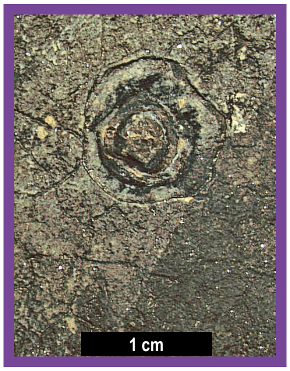

Spheroidal; large central chamber surrounded by thick wall and second tissue layer; outermost layer thin.

DT 146 Galling

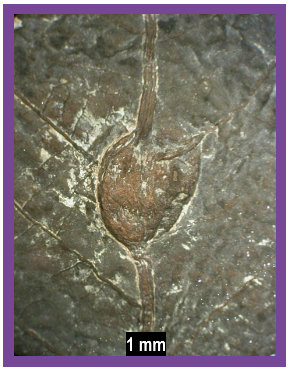

Large, unilocular, hemispherical, with smooth surface and thickened base; on midrib.

DT 147 Galling

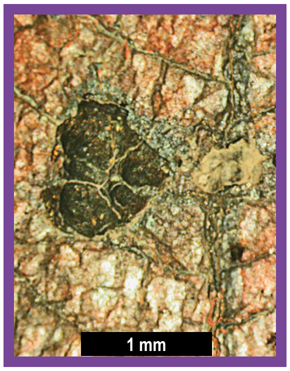

Small, irregular shape, thickened; with internal partitions representing continuation of tertiary venation.

DT 149 Galling

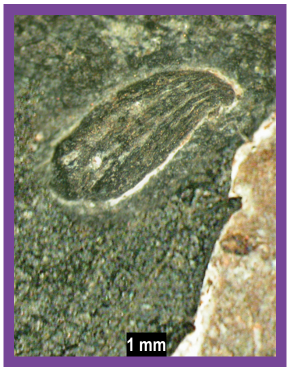

Cylindrical to ovoidal, interveinal; with prominently ribbed exterior and short, pedunculate base.

DT 150 Galling

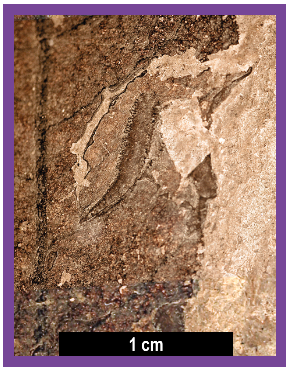

Elongate, attached to 2° veins; thick wall with transverse structure, smooth surface and acute tip.

DT 169 Galling

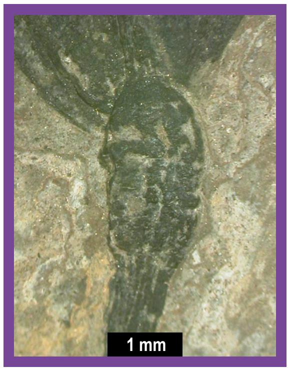

Large globose, fleshy gall with a slit-like aperature; occurring on petiole.

DT 186 Galling

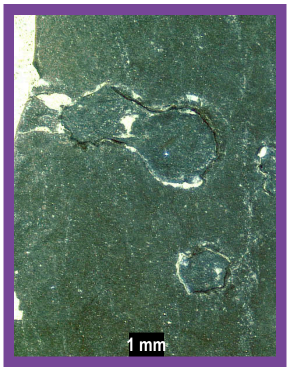

Deciduous, hemispheroidal galls up to 5 mm diameter with thickened outer layer and anchored to deep foliar tissues by a constricted but broad pedicel,

DT 188 Galling

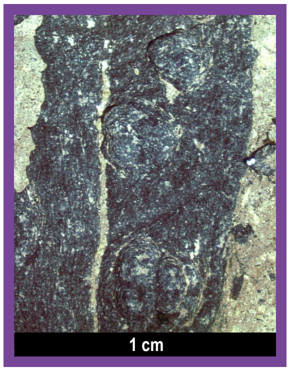

Large, compound, foliar, smooth-surfaced galls; spheroidal-lobate in shape with up to 8 individual locules; occurring on leaf midblade area between midrib

DT 189 Galling

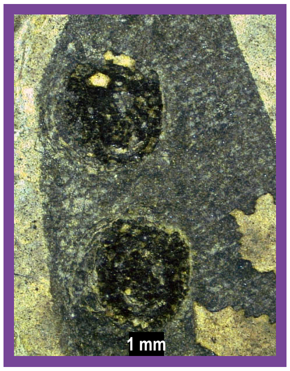

Large compound galls subdivided by tertiary venation into distinct dark-hued single-chamber subunits; enveloped by an outer indurated layer of confluent tissue.

DT 190 Galling

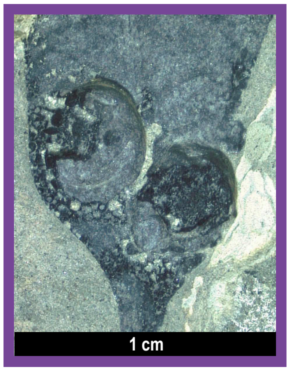

Circular to somewhat ellipsoidal to even narrowly ellipsoidal scales with a central, concentric dark region and lighter-hued concentric rings towards the margin; often

DT 194 Galling

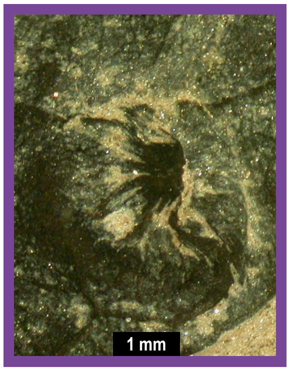

Bascal attachment scar of a large, probably deciduous pedanculate gall, occurring in thickened foliar tissue, characterized by radiatory ridges forming a central tubular

DT 197 Galling

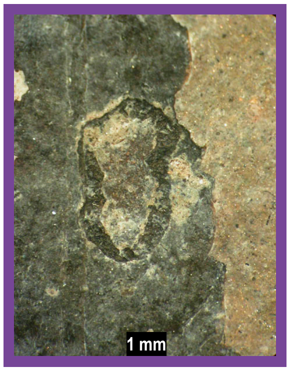

Ovoidal, elliptical, to somewhat irregularly margined bister galls with a variable broad, thickened margin of transverse lineations and a lighter hued, finer-textured

DT 199 Galling

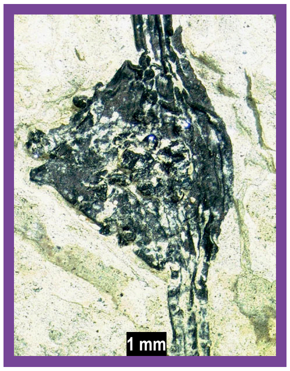

Petiolar or twig galls ovoidal to spheroidal in shape beavin a distinctive pointed, lipped ostcole, a central chamber containing dark, rounded, maccuated

DT 204 Galling

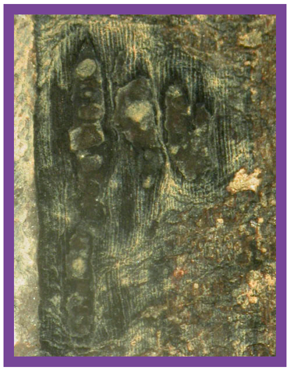

A linear series of up to 15 circular to elongate galls occurring adjacent to one another and bounded laterally by parallel major veins, each with a dark, thick

DT 205 Galling

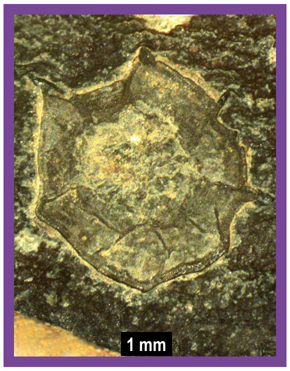

Distinctive, deciduous, short, pedunculate, spinose gall of an overall elliptical shape and a central chamber with particulate material; approximately 15 short

DT 206 Galling

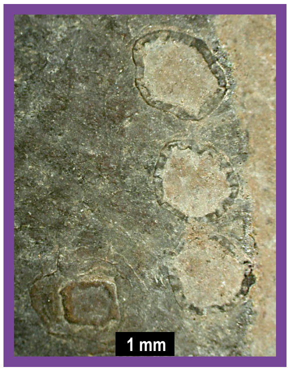

Several small, circular, single chambered galls aligned end-to-end in a linear sequence parallel to the primary plant-host venation.

DT 209 Galling

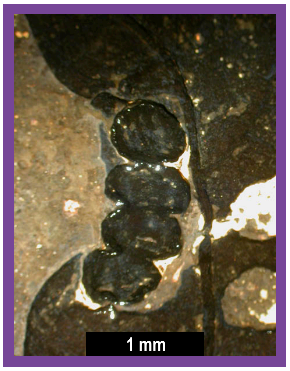

A linear series of 3 to 4 equisized circular to broadly ellipsoidal opaque galls, each with a dark outer cover and a single, cupacious chamber, occurring on

DT 213 Galling

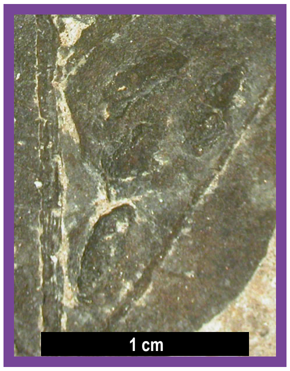

Deciduous, elongate elliptical nonspinose galls attached sessilely to leaf blade with a smooth-surfaced outer indurated layer and a single ellipsoidal inner

Hole Feeding

Hole Feeding

DT 148 Hole Feeding

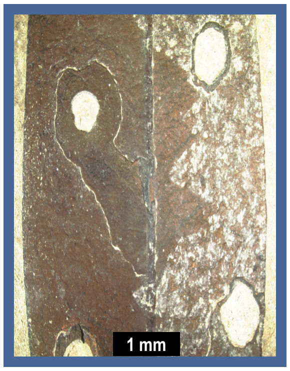

Circular holes with a broad flange of reaction tissue, often extending to enveloping polylobate area.

DT 212 Hole Feeding

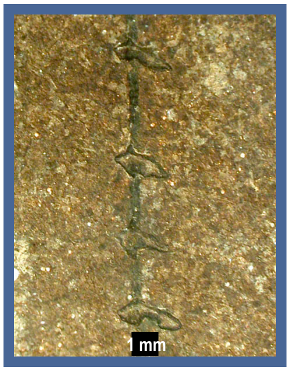

A dumbbell-shaped mandible bite mark on either side of a midrib or other primary vein, with bite enlarged toward leaf margin and joined over the vein, forming a

Margin Feeding

Margin Feeding

DT 142 Margin Feeding

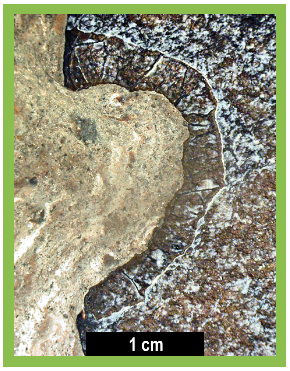

Cuspate margin feeding with a very broad rim of transverse lineations.

DT 143 Margin Feeding

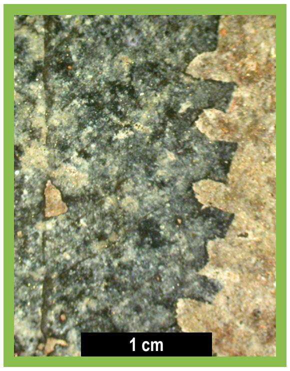

Three or more serial, cuspate margin excisions, separated by small leaf-margin segments.

DT 198 Margin Feeding

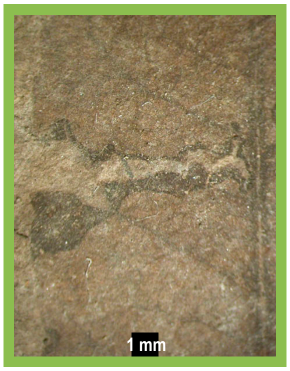

Highly trenched, narrow margin feeding with extensive necrotic flaps or adjacted necrotic tissue and projecting veinal stringers, frequentliy extending to the midrib

DT 200 Margin Feeding

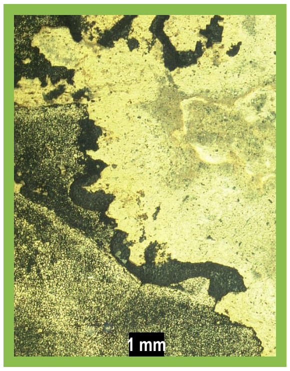

Cuspate to nearly-trenched margin feeding with an opaque, thickened rim of reaction tissue organized into inverted cuspules and projecting flaps.

Mining

Mining

DT 170 Mining

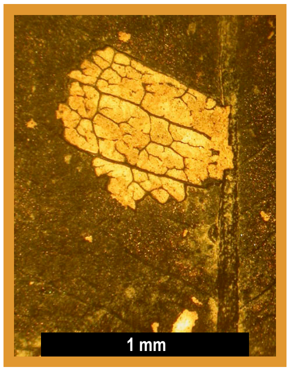

Circular to ellipsoidal (rectangular) mined areas, small veins of the leaf have been left intact over the mined areas, apparently consumption of parenchyma by

DT 171 Mining

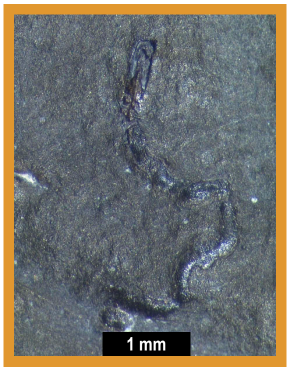

Small meandering, frass-filled mine with rimmed, circular, terminal chamber of tiny coprolites.

DT 173 Mining

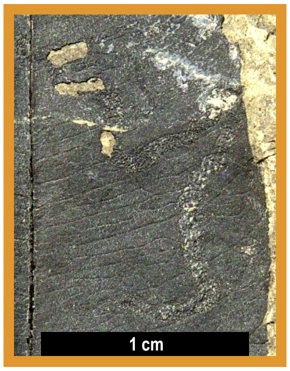

Long serpentine mine; enlarged oviposition site; frass trail intestiniform, of rounded angulate loops; terminal chamber large.

DT 176 Mining

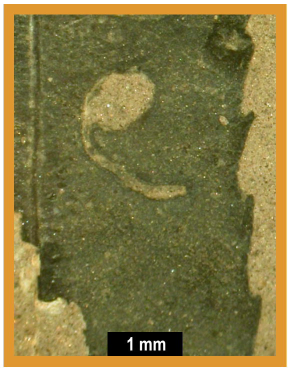

Small (<10 mm) mine with a short, linear to curved early phase, and a much expanded ovoidal to circular terminal phase. (117)

DT 182 Mining

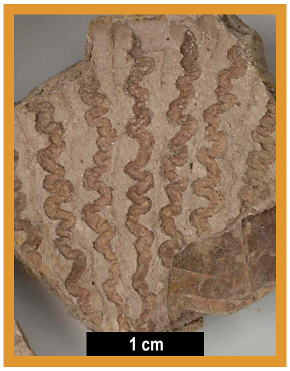

Multiple (up to 8) sinusoidal mines (cu 1.5 mm diameter and at least 30 mm long) originating from a single oviposition event and following a parallel, co-ordinated

DT 185 Mining

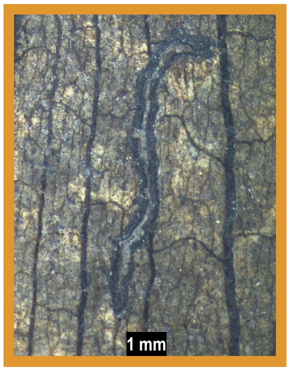

Curvilinear to broadly serpentine leaf mine with an exceptionally thickened margin an dapparently devoid of identifiable internal contents such as coprolites, and

DT 187 Mining

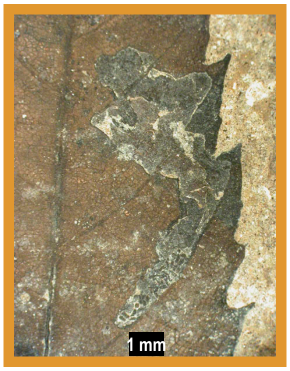

Medium-sized (ca. 15 mm x 10 mm) ovoidal blotch mines with a pronounced outer sinusoidal margin containing submillimeter sized ellipsoidal coprolites

DT 193 Mining

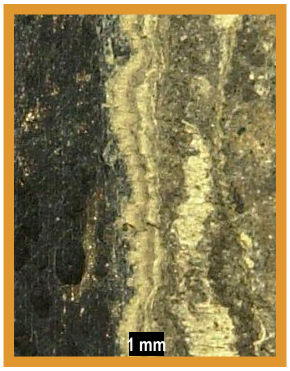

Delicate, graule leaf mine at edge of a parallel-veined leaf characterized by comparitively wide affected area (ca. 2 mm) with a distinct reaction rim and a very

DT 195 Mining

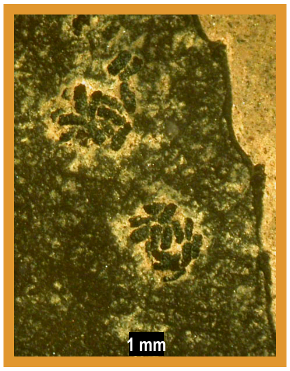

Circular to broadly ellipsoidal to elongate mine-like circular excavations of inner tissue dominated by an inner cluster of 10-20 elongate parallel-sided

DT 208 Mining

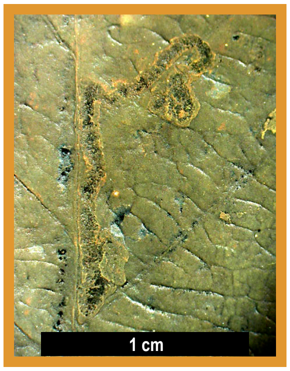

Mine consisting of an initial enlarged region containing the oviposition site and first few small larval instals followed by a significantly narrower, curvilinear

DT 210 Mining

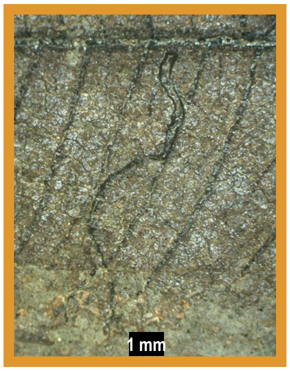

Small, initially hariline wide mine, enlarging in width to ca. 1.0 mm, containing circular to ellipsoidal frass placed along the medial axis at regular intervals in the last

Piercing & Sucking

Piercing & Sucking

DT 157 Piercing & Sucking

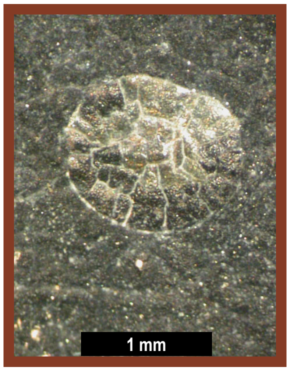

Circular scale marks with radial striae along margin and central disrupted region.

DT 168 Piercing & Sucking

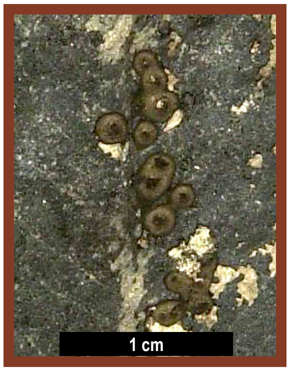

Small (1mm diam.) covering scales including earlier waxy instars; numerous concentric lineations.

DT 183 Piercing & Sucking

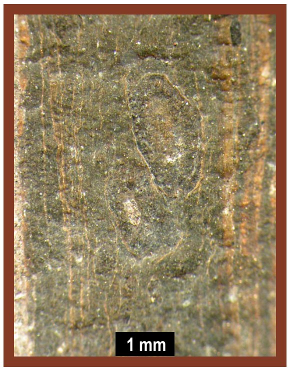

Broad ellipsoidal to circular (0.3 -0.5 mm wide x 0.6-0.9 mm long) raised regions nestled brething two boundary adjacent, parallel veins; with distinct outer,

DT 191 Piercing & Sucking

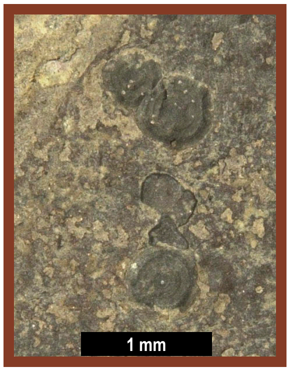

Circular to variably rounded scales with numerous, often perfectly concentric very fine rings (up to 60), definted by 4-12 thicker, dark concentric bands, ca.

Skeletonization

Skeletonization

DT 192 Skeletonization

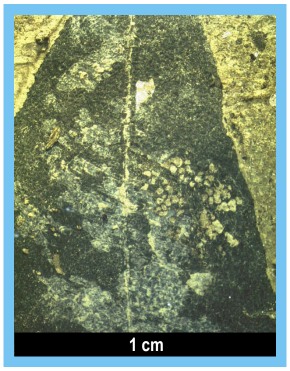

Broad, curvilinear skeletonized area in costal leaf region between two adjacent, major, secondary veins exhibiting totally consumed leaf cells surrounded by resistant,

Surface Feeding

Surface Feeding

DT 196 Surface Feeding

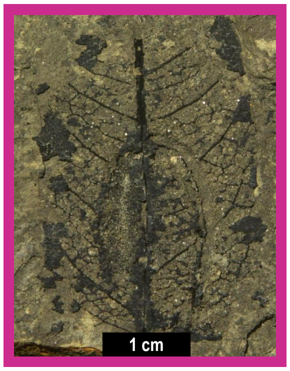

Approximately symetrically removed surface tissues adjacent to both sides of a midrib or other primary vein, do defined by a curvilinear marginward reaction

DT 201 Surface Feeding

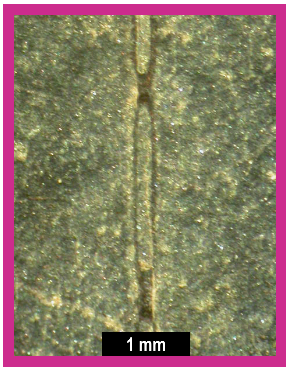

Removal of tissues along the surface of a primary vein, resulting in a concatenzated series of elongate to very elongate scrapings with circular endings

DT 202 Surface Feeding

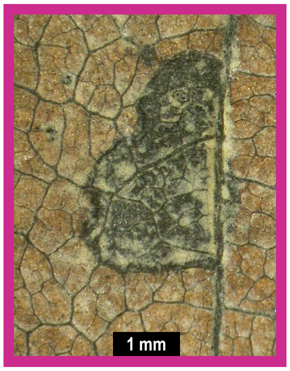

Removal of surface tissue along one side of a leaf bounded by a straight midrib axially and variously sucpate or curvilinear margin toward the leaf margin,

DT 203 Surface Feeding

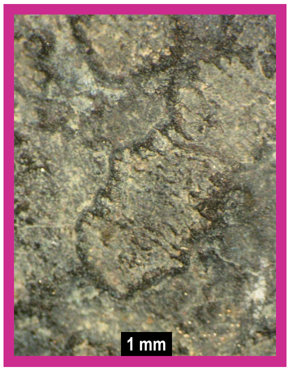

Elongate pattern of surface tissue removal across mid-ranked parallel veins with a prominent reaction rim of dark wedge-like or wispy extensions into the inner

DT 207 Surface Feeding

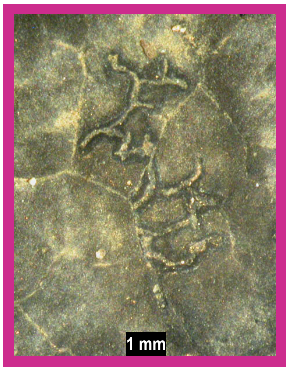

Linear swaths of removed surface tissues, ca. 0.5-1.5 mm wide, combined in radiating criss-crossing branching or veinule-following rectangular patterns,
